# Unveiling the Latent Reactivity of Cp* Ligands (C_5_Me_5_^–^) toward Carbon Nucleophiles on an Iridium Complex

**DOI:** 10.1021/acs.inorgchem.2c04381

**Published:** 2023-04-03

**Authors:** Alejandra Pita-Milleiro, Macarena G. Alférez, Juan J. Moreno, María F. Espada, Celia Maya, Jesús Campos

**Affiliations:** † Instituto de Investigaciones Químicas (IIQ), Departamento de Química Inorgánica and Centro de Innovación en Química Avanzada (ORFEO−CINQA), Universidad de Sevilla and Consejo Superior de Investigaciones Científicas (CSIC), Avenida Américo Vespucio 49, 41092 Sevilla, Spain; ‡ University of Sevilla, 41092 Sevilla, Spain

## Abstract

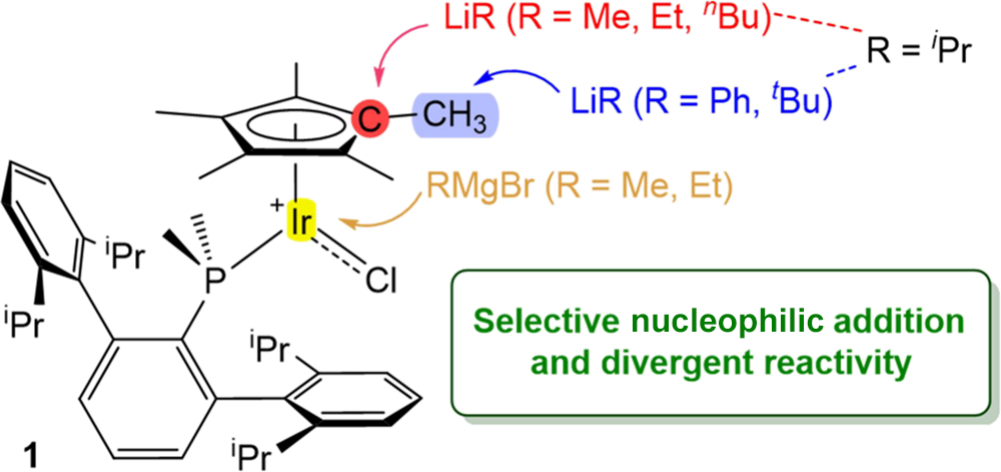

The divergent reactivity of the cationic iridium complex [(η^5^-C_5_Me_5_)IrCl(PMe_2_Ar^Dipp2^)]^+^ (Ar^Dipp2^ = C_6_H_3_–2,6-(C_6_H_3_–2,6-^*i*^Pr_2_)_2_) toward organolithium and Grignard reagents is described. The noninnocent behavior of the Cp* ligand, a robust spectator in the majority of stoichiometric and catalytic reactions, was manifested by its unforeseen electrophilic character toward organolithium reagents LiMe, LiEt, and Li^*n*^Bu. In these unconventional transformations, the metal center is only indirectly involved by means of the Ir(III)/Ir(I) redox cycle. In the presence of less nucleophilic organolithium reagents, the Cp* ligand also exhibits noninnocent behavior undergoing facile deprotonation, which is also concomitant with the reduction of the metal center. In turn, the weaker alkylating agents EtMgBr and MeMgBr effectively achieve the alkylation of the metal center. These reactive iridium(III) alkyls partake in subsequent reactions: while the ethyl complex undergoes β-H elimination, the methyl derivative releases methane by a remote C–H bond activation. Computational studies, including the quantum theory of atoms in molecules (QTAIM), support that the preferential activation of the non-benzylic C–H bonds takes place via sigma-bond metathesis.

## Abstract 2

The divergent reactivity of the cationic iridium complex [(η^5^-C_5_Me_5_)IrCl(PMe_2_Ar^Dipp2^)]^+^ (Ar^Dipp2^ = C_6_H_3_−2,6-(C_6_H_3_−2,6-^*i*^Pr_2_)_2_) toward organolithium and Grignard reagents is described.

## Introduction

Since the serendipitous discovery of ferrocene in 1951,^1,2^ cyclopentadienyl ligands, [C_5_R_5_]^−^, have become indisputably one of the most important ligands in organometallic chemistry and homogeneous catalysis.^3^ In fact, their coordination complexes extend to virtually every metal in the periodic table.^4−6^ Their versatility is evidenced as well by their variable hapticity (from η^1^ to η^5^)^7,8^ and synthetic flexibility. Beyond the foremost and simplest [C_5_R_5_]^−^ ligands, many versions have been developed, including mono and polyfunctionalized derivatives where R accounts for simple alkyl or aryl groups,^9^ or even bulky substituent to access extremely congested cyclopentadienyl ligands,^10−16^ heteroatom-containing fragments for cooperative reactivity with the metal,^17,18^ bridging anchors to access ansa-metallocenes,^19−22^ or chiral moieties to mediate asymmetric catalysis.^23^ However, the permethylated [C_5_Me_5_]^−^ ligand (Cp*) is likely the one that has enjoyed the widest popularity.

**Figure 1:**
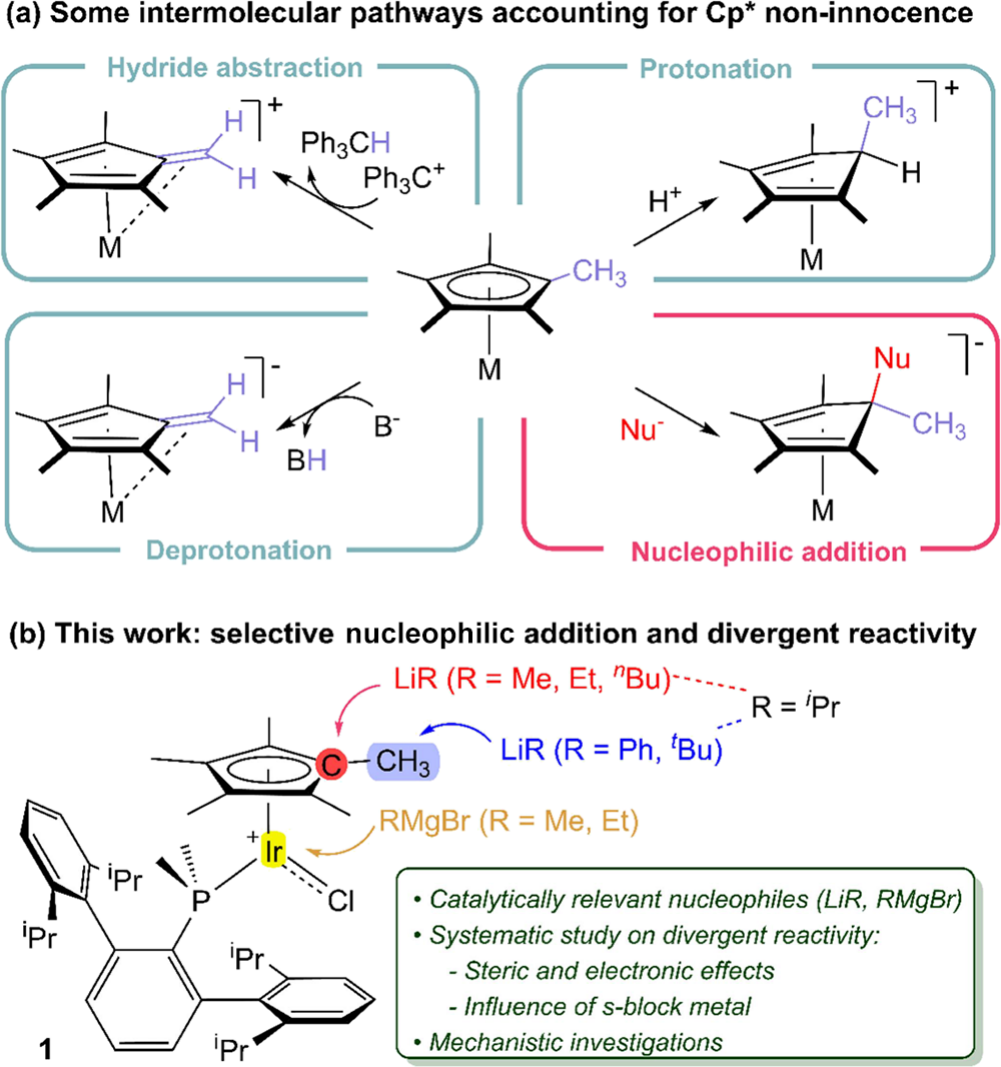
(a) Most common intermolecular pathways for the activation of the Cp* ligand in transition metal complexes; (b) Systematic study revealing divergent reactivity of complex 1 upon reaction with highly polarized carbon nucleophiles.

A crucial driving force for the widespread use of cyclopentadienyl ligands is their robust spectator behavior, which is particularly strong in the case of Cp*. However, even for the later ligand, there are increasing examples of its noninnocent character (Figure 1). The methyl groups of Cp* can partake in several transformations, including, but not limited to, deprotonation by an external base or a bifunctional ligand,^24−37^ hydride abstraction which tends to proceed through single-electron processes,^38−40^ C–H oxidative addition to an adjacent transition metal in bimetallic structures,^41−48^ or direct and reversible methyl-to-metal hydride migration, which was soon identified in early transition metals^49−52^ and recently unlocked by our group as a viable process for late transition metals.^53^ In addition, the protonation of the internal ring has been exploited in proton-couple-electron-transfer (PCET) catalysis capitalizing on the reversible migration of the proton between the ring and the metal.^54−60^ Moreover, several radical routes have been identified for Cp*-containing species resulting as well in ligand functionalization.^61^

In contrast, the reactivity of the internal carbon centers of Cp* toward nucleophiles has only been observed in a limited number of cases,^62^ being more frequent on the less electron-rich and sterically hindered [C_5_H_5_]^−^ upon addition of common highly polar reagents, typically organolithium and organomagnesium compounds.^63−73^ These transformations are of high relevance for a variety of catalytic processes involving cyclopentadienyl catalysts.^74−82^ For instance, cyclopentadienyl nickel and iron complexes are very active Kumada cross-coupling catalysts with organomagnesium reagents^83−88^ or for the polymerization of the latter.^89^ Organolithium and organomagnesium species are also used as initiators for olefin polymerization or diene isomerization with related catalysts.^90,91^ Besides, the use of organolithium and organomagnesium reagents in the presence of Cp*M complexes of both early^92−100^ and late-transition metals^101−109^ have been reported in many occasions, but the direct reactivity of the Cp* ligand has been overlooked in all cases. Cyclopentadienyl ligands, in particular Cp*, continue to be extensively employed in fundamental organometallic chemistry and homogeneous catalysis. On these bases, understanding these unforeseen reactions is crucial to avoid catalyst deactivation^110^ or undesired catalytic outcomes,^111−113^ and to further extend the utility of this platform beyond current capabilities, while gaining insight into the formation of active species from Cp*-bearing precatalysts in the presence of bases.^114−116^

With this goal, we have selected our recently published terphenyl phosphine iridium compound **1** [(η^5^-C_5_Me_5_)Ir(Cl)(PMe_2_Ar^Dipp2^)][BAr_F_] (Ar^Dipp2^ = C_6_H_3_–2,6-(C_6_H_3_–2,6-^*i*^Pr_2_)_2_)^28,117^ to carry out a systematic study of its reactivity toward highly polarized organolithium and organomagnesium reagents. This platform is particularly attractive for these endeavors because (i) it presents a vacant coordination site at the electrophilic Ir(III) center and a chloride ligand susceptible of participating in salt metathesis, yet both the ring and methyl groups of the Cp* can react preferentially toward nucleophiles and/or bases; (ii) the proven noninnocence of the Cp* ligand in this complex, encompassing deprotonation, reversible C–C bond formation and C–H bond breaking;^28^ (iii) its great stability toward cyclometallation;^28^ (iv) the possibility of accessing a bulkier analogue of Bergman’s complex [(η^5^-C_5_Me_5_)Ir(Me)(PMe_3_)(ClCH_2_Cl)]^+^;^116,118^ and (v) the in general prominent position of Cp*Ir complexes in the field of C–H bond activation.^118−124^

## Results and Discussion

**Scheme 1:**
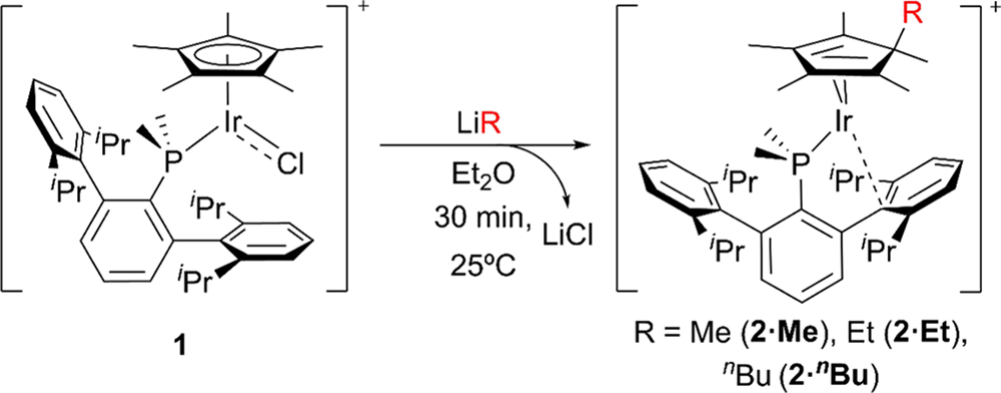
Syntheses of Complexes 2·Me, 2·Et, and 2·^*n*^Bu from 1 and LiMe, LiEt, and Li^*n*^Bu, Respectively

To start this systematic study, we first examined the equimolar reaction of complex **1** with the common nucleophile LiMe. As stated above, and considering the reduced size of the methyl anion, we anticipated the methyl group to either fill the vacancy of this unsaturated Ir(III) complex or replace the chloride to access a Bergman-type complex^118^ [(η^5^-C_5_Me_5_)Ir(Me)(PR_3_)(ClCH_2_Cl)]^+^. To our surprise, the only discernible product, which we fully characterized, is a cationic Ir(I) complex (**2·Me**) featuring a new methyl group bonded to one of the internal carbon atoms of the former Cp* ligand, as shown in Scheme 1. Analogous reactivity was found with lithium alkyls LiEt and Li^*n*^Bu (Scheme 1), whose equimolar addition to the iridium precursor **1** led, respectively, to compounds **2·Et** and **2·^*n*^Bu**, in which a new hydrocarbyl fragment is installed in the exo-face of the parent Cp* ligand.

The room temperature ^1^H NMR spectrum of complex **2·Me** features broad resonances, suggestive of a dynamic solution process. This fluxional behavior arises from the rotation of the C_5_Me_6_ fragment, presumably through a tetrahedral coordination environment,^124,125^ and not from the exchange of the flanking Dipp rings of the phosphine ligand, according to exchange spectroscopy (EXSY) experiments (see Figure S4). However, at −20 °C, complex **2·Me** exhibits a rigid solution structure providing sharp, well-resolved resonances. The absence of symmetry present in **2·Me** results in a complex ^1^H NMR spectrum—six singlets, each with relative intensity corresponding to 3 H, are recorded in the 1.84–0.32 ppm range for the Me groups of the newly formed C_5_Me_6_ diene ligand. Likewise, the four Dipp iso-propyl substituents are inequivalent and originate corresponding multiplets centered at 2.64, 2.32, 2.15, and 2.00 ppm (see Section 2.1 of the SI and Figure S2) for the methine CHMe_2_ protons. As a means to compensate unsaturation, complex **2·Me** features a secondary π-arene interaction^125,126^ with the metal center revealed by the low-frequency shift of one of the ipso carbon atoms of the flanking aryl rings (120.4 ppm, cf. the 135.7 ppm value for the corresponding carbon of the noncoordinated Dipp ring) and further supported by topological analysis and Energy Decomposition Analysis - Natural Orbital for Chemical Valence (EDA-NOCV)^129^ (see Sections 5.16.2 and 5.17.1 of the SI). One of the ortho carbon atoms of this ring seems to also participate in the bonding, resulting in η^2^-coordination of the arene, as its chemical shift (132.5 ppm) is significantly shifted to lower frequencies compared to its counterparts (141.5 ppm for the other ortho carbon within the same ring, and 146.5 and 146.9 ppm for the ones belonging to the nonbound Dipp). Interestingly, the ^13^C{^1^H} NMR spectrum of complex **2·Me** also exhibits a clear difference in the chemical shift of the two pairs of carbons involved in the two formal double bonds of the C_5_Me_6_ unit (122.5 and 114.6 vs 78.3 and 61.9 ppm). This experimental evidence together with the longer C–C distance for the formal double bond trans to the phosphane (C34–C33: 1.431(6) vs C35–C36: 1.338(6) Å) and corresponding closer distance to the metal center (C34–Ir1: 2.118(5) and C33–Ir1: 2.163(4) vs C35–Ir1: 2.265(5) and C36–Ir1: 2.437(4) Å) support our hypothesis that the formal coordinated diene is closer in nature to a double bond and a metalacyclopropane. This can be explained by the stronger trans influence of the phosphane, also observed in a closely related system.^126−128^ EDA-NOCV studies further sustain this idea, revealing a major contribution of the carbon atoms with the longer C–C bond distance to the principal orbital interactions (see Section 5.17.2 of the SI). These spectroscopic features are similar to those found for compounds **2·Et** and **2·^*n*^Bu**, whose full characterization is included in the Supporting Information.

**Figure 2:**
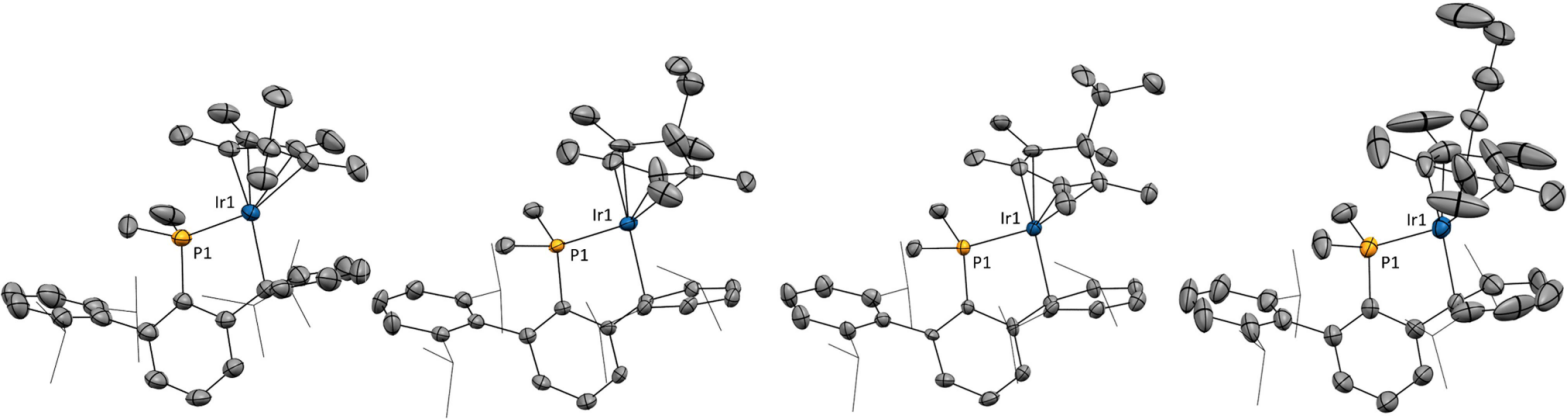
ORTEP diagrams of the cation of complexes **2·Me**, **2·Et**, **2·^*i*^Pr**, and **2·^*n*^Bu**. Hydrogen atoms are excluded for clarity and thermal ellipsoids are set at 50% probability. Wireframe is used to represent the *iso*-propyl groups.

The molecular formulation of the new compounds was corroborated by X-ray diffraction studies, confirming the exo attack on the Cp* and revealing a preferred η^1^-arene coordination in the solid state, rather than η^2^-binding as inferred from spectroscopic analysis. Thus, in complex **2·Me**, the Ir–C_arene_ bonding is characterized by an Ir–C_ipso_ bond distance of 2.249(4) Å, and by significantly longer, and therefore weaker, Ir–C_ortho_ interactions of length 2.544(5) and 2.686(4) Å (Figure 2). Similar geometric parameters are found in compounds **2·Et** and **2·^*n*^Bu**, with notably shorter Ir–C_ipso_ (2.231(5), **2·Et**; 2.253(5) Å, **2·^*n*^Bu**) distances compared to Ir–C_ortho_ (2.549(5), **2·Et;** 2.593(6) Å, **2·^*n*^Bu**) interactions.

**Figure 3:**
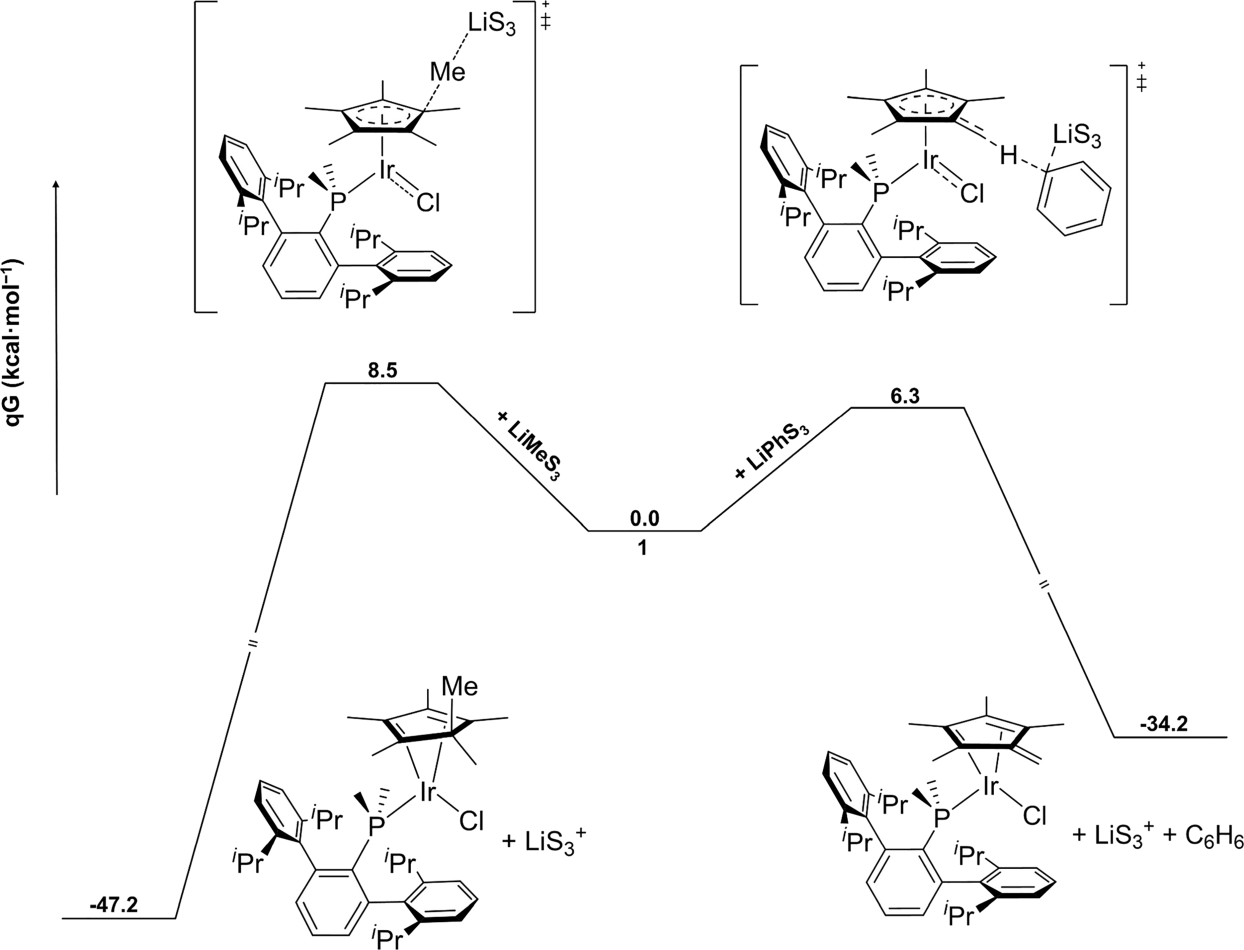
Free energy profiles of LiMe attacking one of the internal carbon atoms of the Cp* moiety (left) and LiPh abstracting a proton from one of the methyl groups of the Cp* moiety (right). S = Me_2_O.

We carried out Density Functional Theory studies to gain insight into the mechanism of the reactions depicted in Scheme 1. For convenience, we focused on the relatively simpler LiMe. Our attempts to rationalize this reactivity through classical foregoing routes involving reductive coupling processes between the Cp* ligand and an Ir—Me functionality failed to provide energy barriers in agreement with experimental observations (see Figures S25 and S26). This led us to explore a more unconventional reaction pathway in which the metal center does not directly participate, and, instead, the direct attack of the LiMe molecule to the exo face of the Cp* moiety takes place. The transition state of the C–C bond formation step requires surmounting a barrier of only 8.0 kcal/mol and yields a neutral Ir(I) complex at −39.2 kcal/mol relative to the reactants. Subsequent chloride release assisted by the solvated lithium atom gave complex **2·Me** through an accessible barrier (see Figures 3 and S24).

**Scheme 2:**
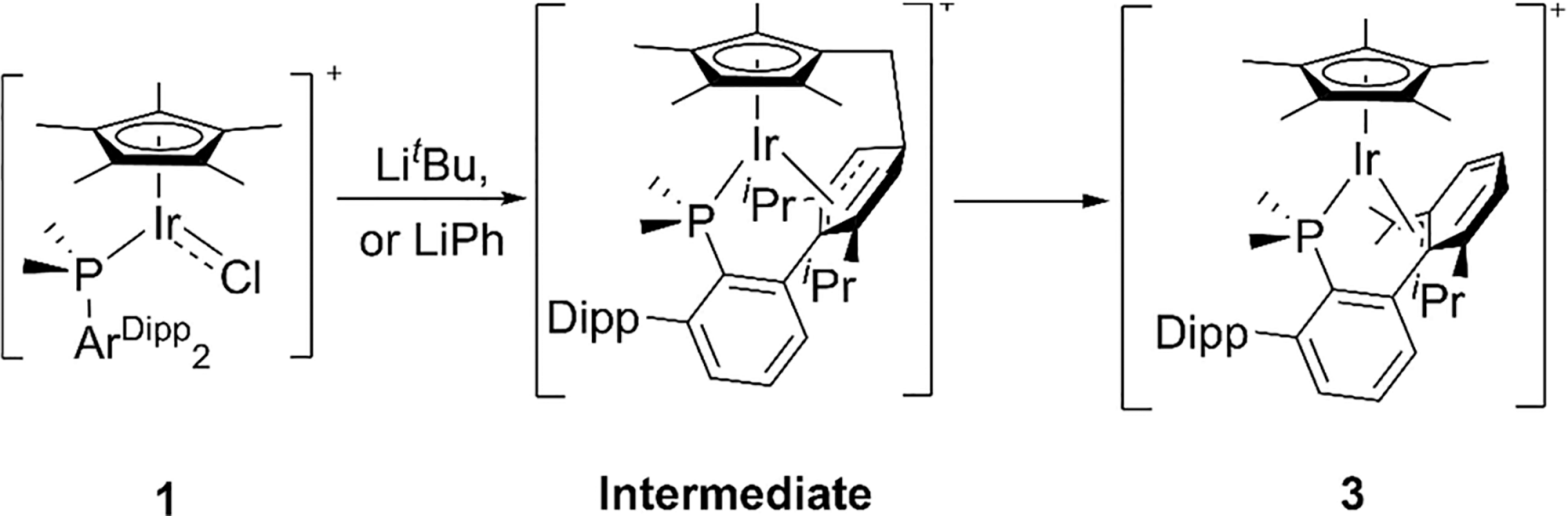
Obtention of Complex 3, Final Product of the Reaction between Complex 1 and Li^*t*^Bu or LiPh Conditions: alkyl lithiums were added at −78 °C; solution was left to reach room temperature.

In contrast, the less nucleophilic lithium alkyls LiPh and Li^*t*^Bu acted instead as Brønsted-Lowry bases deprotonating one of the methyl groups of the Cp* moiety. As shown in Scheme 2, this event triggers a complex rearrangement involving reversible C–C bond formation that leads to a pseudoallylic structure, complex 3, previously reported by our group by the reaction with the much milder base NEt_3_.^28^ It is remarkable that the unexpected electrophilicity of the internal carbon atoms of the C_5_Me_5_ ring outcompetes the mild although well-known Brønsted-Lowry acidity of the C–H bonds, even with bases around 40 p*K*_a_ units stronger than NEt_3_.

To rationalize such striking divergence in reactivity, DFT studies were performed to calculate the energy profiles for LiPh acting as a nucleophile or as a base. In agreement with the experimental observations, a lower energy barrier for the deprotonation step (6.3 kcal/mol, Figure 3) was obtained in comparison to the attack to an internal carbon atom of the Cp* (8.3 kcal/mol, see Figure S22). In both cases, square-planar Ir(I) species appear to be key intermediates. For completion, the energy barrier for the proton abstraction by the LiMe molecule was also calculated obtaining a TS at 11.5 kcal/mol, and thus, higher than the one belonging to the methylation pathway (see Figure S23).

Bearing in mind the contrasting reactivity of alkyl lithium reagents, in particular Li^*n*^Bu and Li^*t*^Bu, we wondered about the outcomes of an intermediate situation in terms of steric and electronic properties of the carbon nucleophile. Thus, we examined the reactivity of **1** with one equivalent of Li^*i*^Pr. Not surprisingly, iso-propyl lithium finds its place between the two aforementioned cases, as the reaction between complex **1** and Li^*i*^Pr yields a mixture of complex **2·^*i*^Pr** and complex **3** in a ca. 1:7 ratio, along with other minor unidentified species. Although complex **2·^*i*^Pr** could not be isolated in pure form, we could monitor its formation, along with that of **3**, by ^31^P{^1^H} NMR spectroscopy (Figure S8) and characterize it crystallographically (Figure 2). The formation of a mixture is consistent with the close DFT-calculated barriers for the deprotonation and nucleophilic attack pathways (Figure S34).

**Figure 4:**
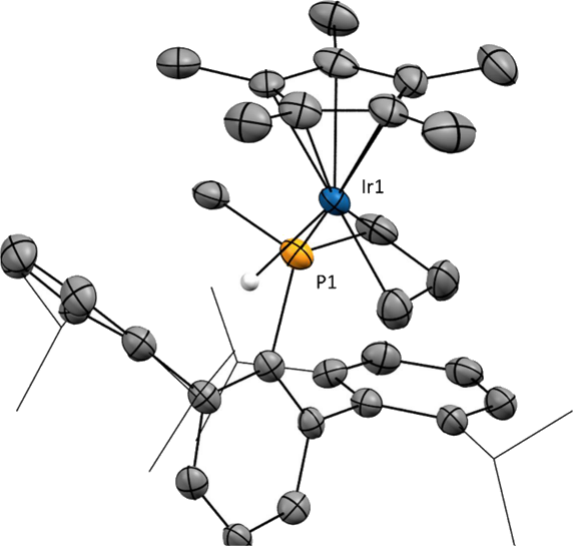
ORTEP diagram of the cation of complex **4**. All hydrogen atoms but those of the hydride are excluded for clarity and thermal ellipsoids are set at 50% probability. Wireframe is used to represent the *iso*-propyl groups.

**Scheme 3:**
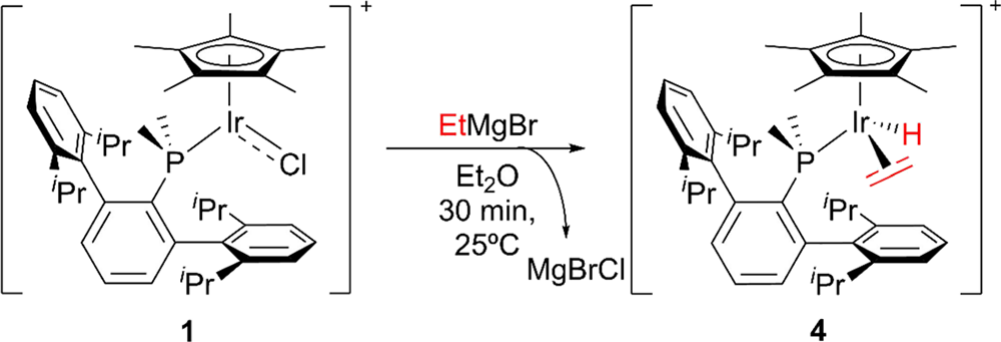
Synthesis of Complex 4 from 1 and EtMgBr

As introduced earlier, organolithium reagents have been widely used in the chemistry of Cp*-containing complexes. Nonetheless, the weaker alkylating Grignard reagents have been even more commonly used and their implications in catalysis are broader.^90,91^ Therefore, we explored the reactivity of complex **1** toward less-polarized Grignard reagents. The addition of equimolar amounts of EtMgBr to diethyl ether solutions of the cationic chloride complex 1 resulted in an instantaneous color change from dark to orange due to the formation of a new species, complex **4** (Scheme 3). In stark contrast to the reactivity exhibited by organolithium reagents, the integrity of the Cp* ligand remains intact when milder Grignard reagents are used, which represents a remarkable divergent reactivity associated to common chemicals that are on many occasions used indistinctly. The coordinatively saturated complex **4** features a ^31^P{^1^H} NMR resonance at −27.0 ppm, therefore showing a large δ shift relative to that of complex **1** (6.6 ppm) and closer to free PMe_2_Ar^Dipp2^ (−41.3 ppm), supporting the absence of the aforementioned Ir–C_arene_ π-interactions.^126,129,130^ A distinctive low-frequency doublet in the ^1^H NMR spectrum (δ −14.9 ppm, ^2^*J*_HP_ = 30.2 Hz) indicates the presence of an iridium hydride, while a coordinated ethylene molecule gives rise to two resonances at 2.18 and 1.88 ppm. X-ray diffraction studies confirmed the proposed formulation and revealed a C–C bond length of 1.426(1) Å (Figure 4) for the ethylene ligand, as expected, longer than that of noncoordinated ethylene (1.3305 Å).^129^ A reasonable proposal for the mechanism of the reaction leading to complex **4** is the substitution of the chloride ligand by an ethyl group with concomitant precipitation of LiCl, followed by β-hydride elimination. Further insight into this proposed mechanism was obtained by DFT studies (see Figure S21). These revealed a low barrier (3.1 kcal/mol) for the formation of an agostic interaction^131−133^ between a C–H bond of the CH_3_ end of the ethyl group and the Ir atom, followed by almost barrierless β-hydride elimination (Δ*G*^‡^ = 0.1 kcal/mol, relative to the agostic complex). These low barriers are congruent with experimental observations, as attempts to spectroscopically detect the Ir–Et intermediate were unsuccessful even at low temperatures.

**Figure 5:**
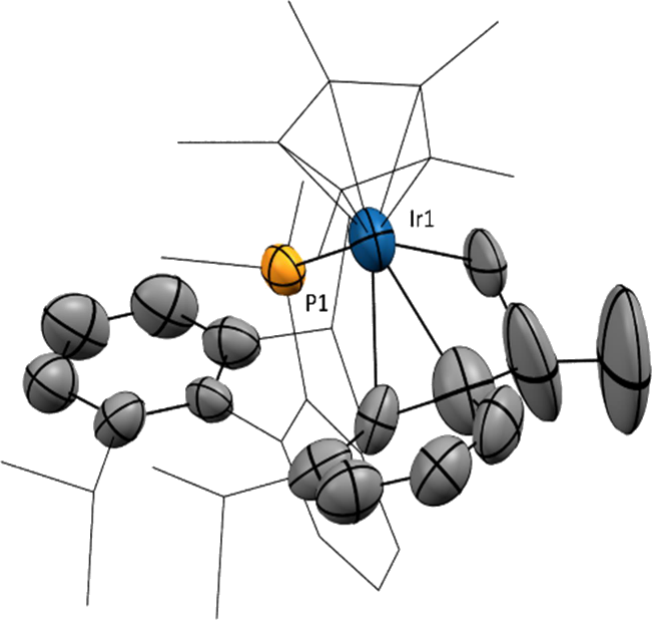
ORTEP diagram of the cation of complex **5**. Hydrogen atoms are excluded for clarity and thermal ellipsoids are set at 50% probability. Wireframe is used to represent the Cp* ligand, the central aryl group of the phosphine, and the *iso*-propyl groups.

**Scheme 4:**
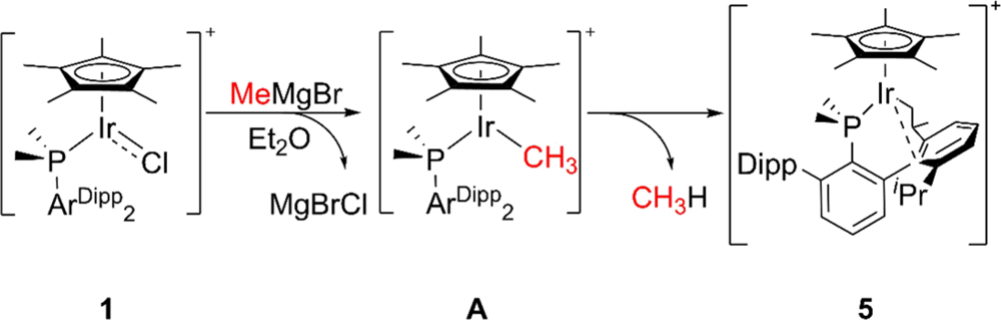
Synthesis of Complex 5 from 1 and MeMgBr through Proposed Intermediate Complex A

The results described above promised the obtention of the analogue of Bergman’s complex reacting MeMgBr with complex **1** due to the lack of hydrogen atoms in the β position in the expected Ir–Me complex. Once more, the use of a magnesium reagent circumvented the direct nucleophilic attack to the Cp* ring, which remained unaltered, yet the observed product of this reaction was complex **5**, derived from the remote and selective activation of a non-benzylic C(sp^3^)–H bond of one iso-propyl group of a lateral terphenyl ring (Scheme 4). At variance with the analogous ethyl reagent, the methyl fragment does not remain at the structure of **5** and instead evolves as methane, which could be observed by careful NMR monitoring (^1^H NMR at 0.23 ppm). This reactivity also contrasts with the cyclometallation selectivity previously shown by this system, where the benzylic methine C–H bond is more amenable to activation.^28^ Complex **5** was fully characterized by multinuclear NMR spectroscopy. Three distinctive ^1^H multiplets, at 3.35, 0.73, and 0.22 ppm, each with relative intensity corresponding to 1 H, were assigned to the CH and the diastereotopic protons of the CH_2_ of the Ir–CH_2_CHCH_3_ moiety, respectively. The molecular structure was authenticated by X-ray diffraction studies, which also indicate that the metal center achieves coordinative saturation by means of an η^2^-arene interaction with the flanking arene (Figure 5). Other geometrical parameters are similar to previous complexes and do not require further discussion.

The mechanism of the reaction depicted in Scheme 4 was also studied through the DFT methodology. The direct attack of MeMgCl to the iridium center yielding a neutral Ir(I) complex was found to be inaccessible (Figure S28), which led us to explore an alternative mechanism. The formation of the Bergman’s type Ir–CH_3_ complex^134^ (**A** in Scheme 4) commences through the magnesium-assisted chloride release (Δ*G*^‡^ = 21.0 kcal/mol), yielding a dicationic Ir(III) complex at 11.4 kcal/mol. This readily reacts with the generated (MeMgCl_2_) moiety, alkylating the metal center with concomitant release of MgCl_2_ (Δ*G*^‡^ = 19.7 kcal/mol) and leading to intermediate **A** at −17.8 kcal/mol relative to the reactants (Figure S27). For comparison, the reaction pathway of MeMgCl attacking one of the internal carbon atoms of the Cp* was also calculated. This route involves a higher-in-energy TS (28.1 kcal/mol), which explains the selectivity of the reaction between **1** and MeMgCl (Figure S27).

**Figure 6:**
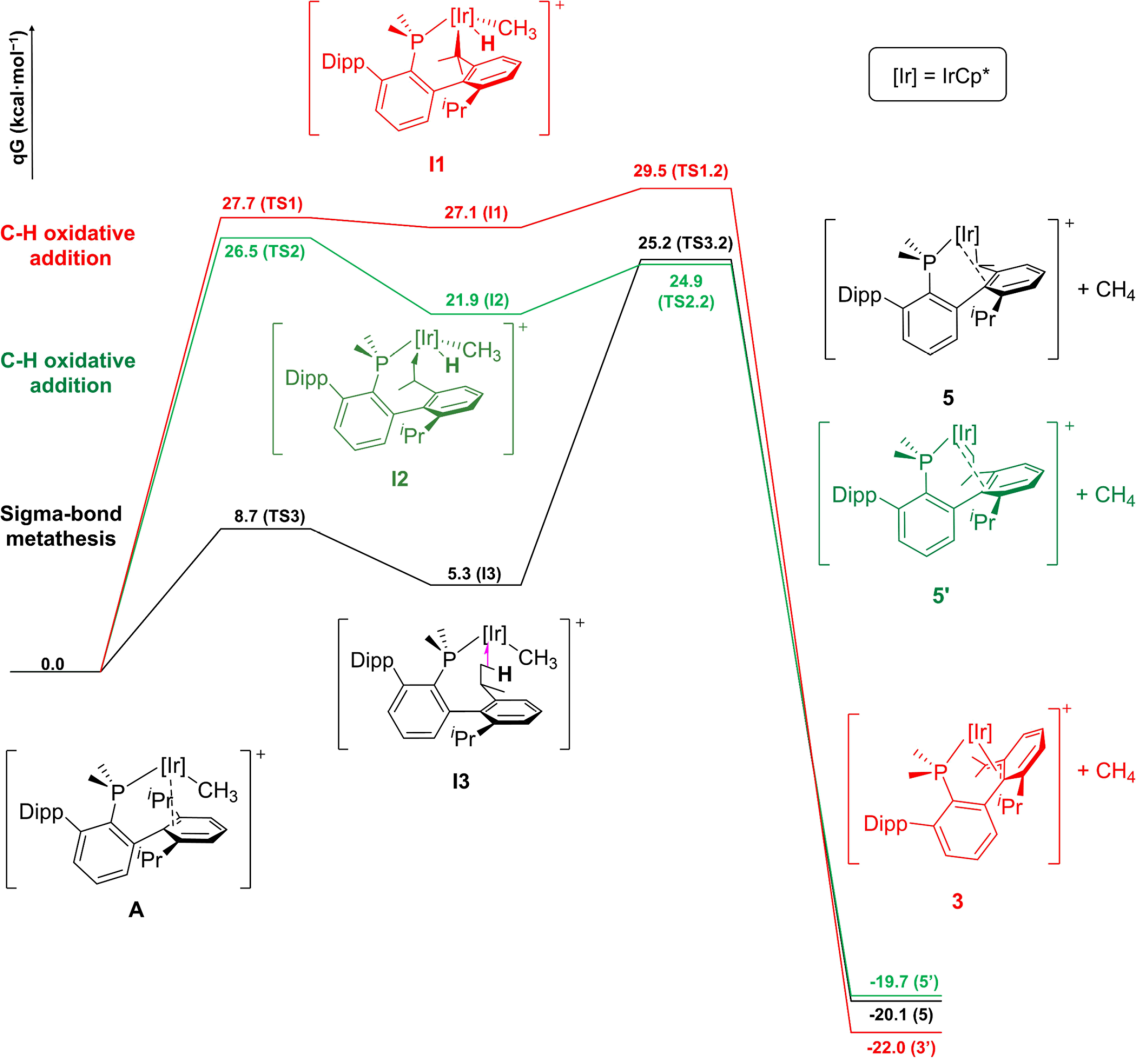
Three different reaction pathways for the elimination of methane from the proposed Ir(III) intermediate **A**. Zero energy corresponds to that of the optimized Ir(III) methylated complex. **5′** is a diastereoisomer of **5**.

We evaluated three different pathways for the release of methane from intermediate **A**, comprising the activation of either one of the two methyl termini of an iso-propyl group or the methine CH. Despite benzylic C–H bonds being usually more prone to metalate, the connectivity of **5** points in a different direction, as supported by our computational studies (Figure 6). As observed experimentally, the activation of the methyl groups is kinetically favored relative to the benzylic methine, despite the latter yielding the most stable product. Notably, the activation of the distinct methyl groups follows different mechanisms: in one case, formation of an agostic interaction^133^ leads to sigma bond metathesis (Figure S28 and Table S3). In contrast, the activation of the other methyl group, as well as for the methine CH, involved the formation of Ir(V) hydride complexes as intermediates.

When a pure solution of complex **5** was able to evolve at room temperature for 5 days, its ^31^P{^1^H} NMR spectrum revealed the emergence of two new peaks at 9.4 and 8.2 ppm. After heating this solution at 80 °C for 5 h, conversion to the species resonating at 9.4 ppm, identified as the thermodynamically more stable complex **3**, was complete. As the reaction between **1** and MeMgBr was originally carried out in a closed *J. Young* tube, one possible isomerization mechanism would be the reaction of **5** with CH_4_ leading to obtaining the most stable compound **3**. However, DFT calculations showed that the energy barrier would be too high (Δ*G*^‡^ = 45.3 kcal/mol) (Figure 6), and experimentally, we found that isomerization also took place in the absence of CH_4_. Although the complex resonating at 8.2 ppm could not be isolated, its multinuclear NMR pattern perfectly fits with its assignment as a diastereoisomer of **5** (compound **5′** in Figure 6) resulting from the activation of the alternative methyl group (Figures S18–S20). DFT calculations for the isomerization process are currently ongoing.

## Conclusions

In conclusion, we demonstrate that the noninnocent character of the widespread Cp* ligand in the presence of strongly polarized alkylating reagents is highly dependent on the nature of the carbon nucleophile. Thus, we identify up to three dissimilar reaction outcomes for the same Ir(III) precursor depending on the substrate employed. First, the Cp* displays an uncommon but clear electrophilic character toward unhindered lithium alkyls—LiMe, LiEt, Li^*n*^Bu—undergoing alkylation of one of the internal carbon atoms of the Cp* ring through a direct nucleophilic attack to its *exo* face, leading to the formal reduction of the metal toward Ir(I) complexes. In contrast, less nucleophilic lithium reagents such as LiPh and Li^*t*^Bu act as Brønsted bases, effecting the deprotonation of a methyl group of the Cp* ring. This event triggers a rearrangement that leads to the formation of a previously reported pseudoallylic structure. Interestingly, the use of Li^*i*^Pr, with intermediate steric and electronic properties, leads to a mixture of the aforesaid structures. In stark contrast, the use of weaker alkylating magnesium agents, which tend to exhibit similar chemistry to organolithium compounds in the context of transition metal alkylations, enables the selective alkylation of the metal center, while the Cp* ligand remains intact. Moreover, a series of subsequent C–H bond activation events have been disclosed for the resulting iridium complexes.

Overall, the foregoing results represent a clear illustration of both the different reactivity of some of the most common reagents in organometallic chemistry, Grignard and organolithium reagents, in many cases exchangeable, and the noninnocent behavior of the Cp* ligand, which continues to be one of the most utilized ligands in organometallic chemistry. Gaining a deep understanding of reactions where Cp* ligand is not as a mere spectator, but an active contributor, is of crucial importance for the discovery of novel transformations and the development of future catalytic processes that rely on the use of this and related ligand frameworks. Indeed, the results of this study advise taking a fresh look at the comprehensive body of work on cyclopentadienyl-based transition metal catalysts that operate in the presence of strongly polarized organometallic reagents, nucleophiles, and bases.

## Supporting Information Available

Experimental procedures; spectra; as well as XRD and computational studies (PDF)
Computational Studies coordinates (XYZ)

## Supplementary Material

ic2c04381_si_001.pdf

ic2c04381_si_002.xyz
