# Supplementary material for: Unveiling the Latent Reactivity of Cp* Ligands (C5Me5–) toward Carbon Nucleophiles on an Iridium Complex
Source: Inorg Chem. 2023 Apr 3;62(15):5961–71. doi: 10.1021/acs.inorgchem.2c04381 (PMC10114086; doi:10.1021/acs.inorgchem.2c04381)
Supplement: Supplementary file 1 — ic2c04381_si_001.pdf [file ic2c04381_si_001.pdf]

Unveiling the latent reactivity of the Cp* ligand ($C_5Me_5^-$) towards carbon nucleophiles on an iridium complex

Alejandra Pita-Milleiro, Macarena G. Alférez, Juan J. Moreno,* María F. Espada, Celia Maya, Jesús Campos*

Instituto de Investigaciones Químicas (IIQ), Departamento de Química Inorgánica and Centro de Innovación en Química Avanzada (ORFEO-CINQA), Universidad de Sevilla and Consejo Superior de Investigaciones Científicas (CSIC), Avenida Américo Vespucio 49, 41092 Sevilla, Spain.

Supporting Information

1. General Considerations 3

2. Preparation of Complexes 3

   2.1 Preparation of complexes 2·Me, 2·Et, 2·^{i}Pr and 2·^{n}Bu 3

   2.2 Preparation of complexes 4 and 5.. 8

3. NMR Spectra of Reported Complexes 10

4. X-Ray Structural Characterization of complexes 31

5. Computational Details 34

   5.1. β-elimination.. 35

   5.2. Comparison: LiPh as a base *versus* as a nucleophile 36

   5.3. Comparison. LiMe attacking the Ir v. attacking one of the internal C of Cp* v. acting as base 37

   5.4. Energy profile for the chloride release to give complex 2·Me. 38

   5.5. Energy profile for reductive coupling (route to obtain complex 2·Me).......... 39

   5.6. Reductive coupling in an associative process to obtain complex 2·Me 40

5.7. Comparison. MeMgCl attacking the Ir v. attacking one of the internal C of Cp* 41

5.8. Alternative route to MeMgCl attacking the metal 42

5.9. Alternatives to the pathway depicted in Fig. 6 43

5.10. Energy profile accounting for the solvation of LiMe 43

5.11. Energy profile accounting for the solvation of MeMgCl 44

5.12. Energy profile accounting for the solvation of Li^+ 45

5.13. Energy profile accounting for the solvation of LiPh 46

5.14. Energy profile accounting for the solvation of Li^{i}Pr 47

5.15. Comparison. Li^{i}Pr attacking one of the internal C of Cp* v. acting as a base48

5.16. Topological Studies 49

5.16.1. Topological analysis of the TS3.2 of Fig. 6 50

5.16.2. Topological analysis of complex 2·Me 51

5.17. EDA-NOCV 53

5.17.1. EDA-NOCV of complex 2·Me: C_5Me_6Ir / $PMe_2Ar^{Dipp}{}_2$ 53

5.17.2. EDA-NOCV of complex 2·Me: C_5Me_6 / $IrPMe_2Ar^{Dipp}{}_2$ 54

6. References 56

1. General Considerations

All manipulations were carried out using standard Schlenk techniques, under high purity nitrogen. All solvents were dried and distilled under nitrogen prior to use. *n*-Pentane (C_5H_{12}) and *n*-hexane (C_6H_{14}) were distilled over sodium. Diethyl ether was distilled over sodium/benzophenone. CH_2Cl_2 and CD_2Cl_2 were dried over CaH_2. THF-d^8 was dried over 4 Å molecular sieves. Complex **1**,[1] and $PMe_2Ar^{Dipp}{}_2$[2] were prepared according to literature methods. Solution NMR spectra were recorded on Bruker DRX-400 and DRX-500 spectrometers. Spectra were referenced to external SiMe4 (δ: 0 ppm) using the residual proton solvent peaks as internal standards (^{1}H NMR experiments), or the characteristic resonances of the solvent nuclei (^{13}C NMR experiments), while ^{31}P was referenced to H_3PO_4. Spectral assignments were made by routine one- and two-dimensional NMR experiments (^{1}H, $^{1}H\{^{31}P\}$, $^{13}C\{^{1}H\}$, $^{31}P\{^{1}H\}$, COSY, HSQC and HMBC) where appropriate. For elemental analyses a LECO TruSpec CHN elementary analyzer was utilized.

Note: in all NMR characterizations, the label *Dipp'* represents the aryl ring featuring an interaction with the Ir center to distinguish it from the other *Dipp* unit.

2. Preparation of Complexes

2.1 Preparation of complexes 2·Me, 2·Et, 2·^{i}Pr and 2·^{n}Bu

To a Et_2O solution (10 mL) of complex **1** (100.0 mg, 0.060 mmol) was added 1.1 equivalents of commercial solution of LiR (R=Me, Et, ^{i}Pr, or ^{n}Bu) at room temperature. There was an instantaneous color change to red. The solution was filtered, the solvent was evaporated under reduced pressure and the residue was washed with pentane (6 mL). Single crystals were grown from a saturated CH_2Cl_2-hexane solution at −32 °C.

2·Me

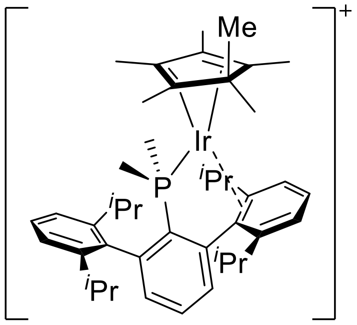

Yield: 56 mg (55 %).

Anal. Calcd. for $C_{74}H_{73}BF_{24}IrP$: C, 54.12; H, 4.42. **Found:** C, 54.21; H, 4.42.

^{1}H NMR (400 MHz, CD_2Cl_2, −20 ºC) δ: 7.71 (m, 8H, *o*-Ar), 7.55 (s, 4H, *p*-Ar), 7.50 (td, 1H, $^3J_{HH}$ = 7.6 Hz, $^5J_{HP}$ = 2.1 Hz, *p*-C_6H_3), 7.43 (m, 2H, *p*-Dipp, *m*-Dipp’), 7.36 (d, 1H, $^3J_{HH}$ = 7.6 Hz, *m*-Dipp’), 7.30 (t, 1H, $^3J_{HH}$ = 7.6 Hz, *p*-Dipp’), 7.28 (d, 1H, $^3J_{HH}$ = 7.7 Hz, *m*-Dipp), 7.23 (d, 1H, $^3J_{HH}$ = 7.8 Hz, *m*-Dipp), 7.20 (ddd, 1H, $^3J_{HH}$ = 7.5 Hz, $^4J_{HP}$ = 2.8 Hz, $^4J_{HH}$ = 0.9 Hz, *m*-C_6H_3), 6.99 (ddd, 1H, $^3J_{HH}$ = 7.7 Hz, $^4J_{HP}$ = 2.0 Hz, $^4J_{HH}$ = 0.9 Hz, *m’*-C_6H_3), 2.64 (sept, 1H, $^3J_{HH}$ = 6.8 Hz, $(CHMe_2)_{Dipp'}$), 2.32 (sept, 1H, $^3J_{HH}$ = 6.7 Hz, $(CHMe_2)_{Dipp}$), 2.15 (sept, 1H, $^3J_{HH}$ = 6.7 Hz, $(CHMe_2)$Dipp), 2.00 (sept, 1H, $^3J_{HH}$ = 6.8 Hz, $(CHMe_2)_{Dipp'}$), 1.86 (d, 3H, $^4J_{HP}$ = 1.1 Hz, C_5Me_6), 1.54 (s, 3H, C_5Me_6), 1.48 (d, 3H, $^2J_{HP}$ = 10.1 Hz, P*Me*Me), 1.35 (d, 3H, $^3J_{HH}$ = 7.0 Hz, $Me_{Dipp'}$), 1.30 (d, 3H, $^3J_{HH}$ = 7.1 Hz, $Me_{Dipp'}$), 1.24 (d, 3H, $^2J_{HP}$ = 10.3 Hz, PMe*Me*), 1.23 (d, 3H, $^3J_{HH}$ = 6.7 Hz, Me_{Dipp}), 1.19 (d, 3H, $^3J_{HH}$ = 6.7 Hz, Me_{Dipp}), 1.13 (s, 3H, C_5Me_6), 1.00 (d, 3H, $^3J_{HH}$ = 6.6 Hz, Me_{Dipp}), 0.90 (m, 6H, Me_{Dipp}, $Me_{Dipp'}$), 0.77 (s, 3H, C_5Me_6), 0.73 (s, 3H, C_5Me_6), 0.70 (d, 3H, $^3J_{HH}$ = 6.8 Hz, $Me_{Dipp'}$), 0.32 (d, 3H, $^4J_{HP}$ = 3.4 Hz, C_5Me_6).

^{13}C{^{1}H} NMR (100 MHz, CD_2Cl_2, −20 ºC) δ: 161.7 (q, $^1J_{CB}$ = 50 Hz, *ipso*-Ar), 146.9 (*o*-Dipp), 146.5 (*o*-Dipp), 146.2 (d, $^2J_{CP}$ = 27 Hz, *o*-C_6H_3), 144.2 (*o*-C_6H_3), 141.5 (*o*-Dipp’), 137.6 (d, $^1J_{CP}$ = 53 Hz, *ipso*-C_6H_3), 135.7 (*ipso*-Dipp), 134.7 (*o*-Ar), 133.8 (d, $^3J_{CP}$ = 5 Hz, *m*-C_6H_3), 133.6 (*m*-Dipp’), 132.5 (m, *m*-Dipp’, *o*-Dipp’), 131.7 (d, $^3J_{CP}$ = 13 Hz, *m*-C_6H_3), 131.1 (*p*-C_6H_3), 129.7 (*p*-Dipp), 128.7 (q, $^2J_{CF}$ = 32 Hz, *m*-Ar), 127.8 (*p*-Dipp’), 124.4 (q, $^1J_{CF}$ = 272 Hz, CF_3), 123.2 (overlapped, *m*-Dipp), 122.9 (*m*-Dipp), 122.5 (d, $^2J_{CP}$ = 15 Hz, *C*$_5$Me$_6$), 120.4 (overlapped, *ipso*-Dipp’), 117.5 (m, *p*-Ar), 114.6 (*C*$_5$Me$_6$), 78.3 (*C*$_5$Me$_6$), 61.9 (*C*$_5$Me$_6$), 56.7 (*C*$_5$Me$_6$), 34.2 ($(CHMe_2)_{Dipp'}$, $(CHMe_2)_{Dipp'}$), 31.4 ($(CHMe_2)_{Dipp}$), 31.2 ($(CHMe_2)_{Dipp}$), 29.7 (C$_5$*Me*$_6$), 26.1 (Me_{Dipp}), 25.7 (Me_{Dipp}), 24.7 (Me_{Dipp}), 24.4 ($Me_{Dipp'}$), 24.2 ($Me_{Dipp'}$), 24.1 ($Me_{Dipp'}$), 21.4 (C$_5$*Me*$_6$), 21.2 (Me_{Dipp}), 21.1 (Me_{Dipp}), 15.7 (d, $^1J_{CP}$ = 39 Hz, PMe*Me*), 15.1 (d, $^1J_{CP} \approx$ 39 Hz, P*Me*Me), 13.4 (C$_5$*Me*$_6$), 12.3 (C$_5$*Me*$_6$), 11.8 (C$_5$*Me*$_6$), 8.3 (C$_5$*Me*$_6$).

^{31}P{^{1}H} NMR (120 MHz, CD_2Cl_2, −20 ºC) δ: 5.1.

2·Et

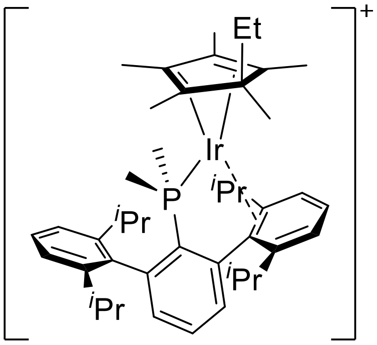

Yield: 49 mg (47 %).

Anal. Calcd. for $C_{76}H_{75}BF_{24}IrP$: C, 54.39; H, 4.50. **Found:** C, 54.31; H, 4.38.

^{1}H NMR (400 MHz, CD_2Cl_2, 25ºC) δ: 7.75 (m, 8H, *o*-Ar), 7.59 (s, 4H, *p*-Ar), 7.53 (td, 1H, $^{3}J_{HH}$ = 7.6 Hz, $^{5}J_{HP}$ = 2.1 Hz, *p*-C_6H_3), 7.47 (t, 1H, $^{3}J_{HH}$ = 7.8 Hz, *p*-Dipp), 7.43 (d, 2H, $^{3}J_{HH}$ = 7.6 Hz, *m*-Dipp'), 7.31 (m, 3H, *p*-Dipp', *m*-Dipp, *m*-Dipp), 7.26 (dd, 1H, $^{3}J_{HH}$ = 7.5 Hz, $^{4}J_{HP}$ = 2.8 Hz, $^{4}J_{HH}$ = 0.9 Hz, *m*-C_6H_3), 7.01 (dd, 1H, $^{3}J_{HH}$ = 7.7 Hz, $^{4}J_{HP}$ = 2.0 Hz, *m*'-C_6H_3), 2.69 (m, 1H, $(CHMe_2)_{Dipp'}$), 2.39 (m, 1H, $(CHMe_2)_{Dipp}$), 2.26 (m, 1H, $(CHMe_2)_{Dipp}$), 2.07 (m, 1H, $(CHMe_2)_{Dipp'}$), 1.90 (br s, 3H, C_5Me_5Et,), 1.58 (m, 6H, C_5Me_5Et, P*Me*Me), 1.41 (d, 6H, $^{3}J_{HH}$ = 6.8 Hz, Me_{Dipp}, Me_{Dipp}), 1.28 (m, 11H, $Me_{Dipp'}$, $Me_{Dipp'}$, PMe*Me*, CH_2CH_3), 0.99 (m, 6H, Me_{Dipp}, Me_{Dipp}), 0.88 (m, 6H, $Me_{Dipp'}$, $Me_{Dipp'}$), 0.79 (s, 3H, C_5Me_5Et), 0.71 (m, 3H, C_5Me_5Et), 0.32 (br s, 3H, C_5Me_5Et), 0.17 (t, 3H, $^{1}J_{HH}$ = 7.3 Hz, CH_2CH_3).

^{13}C{^{1}H} NMR (100 MHz, CD_2Cl_2, 25ºC) δ: 161.8 (q, $^{1}J_{CB}$ = 50 Hz, *ipso*-Ar), 147.3 (*o*-Dipp, *o*-Dipp), 146.8 (d, $^{2}J_{CP}$ = 27 Hz, *o*-C_6H_3), 144.5 (*o*-C_6H_3), 142.9 (*o*-Dipp'), 138.0 (d, $^{1}J_{CP}$ = 53 Hz, *ipso*-C_6H_3), 135.7 (*ipso*-Dipp), 134.8 (*o*-Ar), 134.0 (d, $^{3}J_{CP}$ = 5 Hz, *m*-C_6H_3), 133.4 (*m*-Dipp'), 132.5 (m, *m*-Dipp', *o*-Dipp'), 131.9 (d, $^{3}J_{CP}$ = 13 Hz, *m*-C_6H_3), 130.9 (*p*-C_6H_3), 129.8 (*p*-Dipp), 128.9 (q, $^{2}J_{CF}$ = 32 Hz, *m*-Ar), 127.8 (*p*-Dipp'), 124.1 (m, $^{1}J_{CF}$ = 272 Hz, CF_3), 123.3 (*overlapped*, *m*-Dipp), 123.1 (*m*-Dipp), 121.6 (d, $^{2}J_{CP}$ = 15 Hz, C_5Me_5Et), 120.5 (*overlapped*, *ipso*-Dipp'), 117.5 (m, *p*-Ar), 115.8 (C_5Me_5Et), 80.0 (C_5Me_5Et), 61.4 (C_5Me_5Et), 59.4 (C_5Me_5Et), 34.2 ($(CHMe_2)_{Dipp'}$, $(CHMe_2)_{Dipp'}$), 31.3 ($(CHMe_2)_{Dipp}$), 30.9 ($(CHMe_2)_{Dipp}$), 29.7 (C_5Me_5Et), 26.4 (Me_{Dipp}, $Me_{Dipp'}$), 24.6 (Me_{Dipp}, $Me_{Dipp'}$), 23.1 (Me_{Dipp}, $Me_{Dipp'}$), 21.6 (Me_{Dipp}, $Me_{Dipp'}$), 17.8 ($C_5Me_5CH_2CH_3$), 16.1 (d, $^{1}J_{CP}$ = 39 Hz, PMe_2), 13.3 (C_5Me_5Et), 11.8 (C_5Me_5Et), 10.6 (C_5Me_5Et), 9.4 ($C_5Me_5CH_2CH_3$), 8.4 (C_5Me_5Et).

^{31}P{^{1}H} NMR (162 MHz, CD_2Cl_2 , 25ºC) δ: 4.8.

2·^{i}Pr

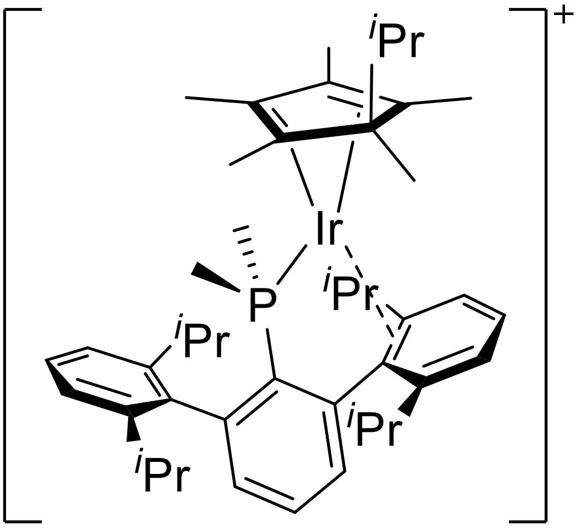

^{31}P{^{1}H} NMR (162 MHz, CD_2Cl_2, 25°C) δ: −0.6.

2·^{n}Bu

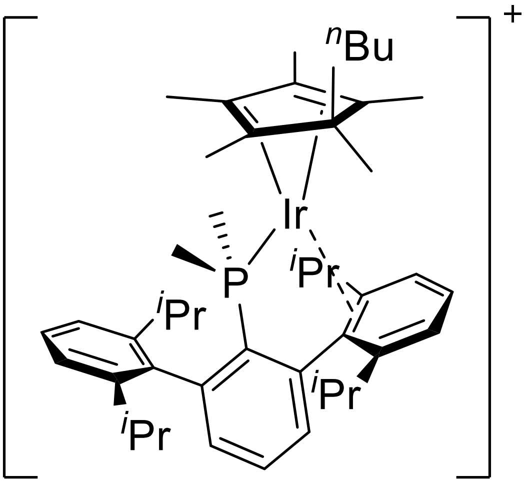

Yield: 28 mg (26 %).

Anal. Calcd. for $C_{78}H_{79}BF_{24}IrP$: C, 54.90; H, 4.67. **Found:** C, 54.81; H, 4.72.

^{1}H NMR (500 MHz, CD_2Cl_2, −20 ºC) δ: 7.72 (m, 8H, *o*-Ar), 7.56 (s, 4H, *p*-Ar), 7.52 (td, $^{3}J_{HH}$ = 7.7 Hz, $^{5}J_{HP}$ = 1.9 Hz, 1H, *p*-C_6H_3), 7.43 (m, 2H, *p*-Dipp, *m*-Dip p'), 7.29 (m, 2H, *p*-Dipp', *m*-Dipp'), 7.22 (m, 3H, *m*-C_6H_3, *m*-Dipp, *m*-Dipp), 6.99 (d, 1H, $^{3}J_{HH}$ = 7.7 Hz , *m*'-C_6H_3), 2.70 (m, 1H, $(CHMe_2)_{Dipp'}$), 2.35 (m, 1H, $(CHMe_2)_{Dipp}$), 2.16 (m, 1H, $(CHMe_2)_{Dipp}$), 1.99 (m, 1H, $(CHMe_2)_{Dipp'}$), 1.86 (s, $C_5Me_5{}^{n}$Bu), 1.54 (s, $C_5Me_5{}^{n}$Bu), 1.50 (d, $^{2}J_{HP}$ = 15 Hz, 3H, P*Me*Me), 1.36 (d, 3H, $^{3}J_{HH}$ = 7.0 Hz, $Me_{Dipp'}$), 1.33 (d, 3H, $^{3}J_{HH}$ = 7.2 Hz, $Me_{Dipp'}$), 1.24 (m, 11H, PMe*Me*, Me_{Dipp},

$C_5Me_5{}^n$Bu, CH_{2nBu},CH_{2nBu}) 1.20 (d, 3H, $^3J_{HH}$ = 6.7 Hz, Me_{Dipp}), 1.06 (m, 2H, CH_{2nBu},CH_{2nBu}), 1.01 (d, $^3J_{HH}$ = 6.7 Hz, 3H, Me_{Dipp}), 0.93 (d, $^3J_{HH}$ = 6.7 Hz, 3H, Me_{Dipp}), 0.90 (d, $^3J_{HH}$ = 6.7 Hz, 3H, $Me_{Dipp'}$), 0.71 (m, 9H, $Me_{Dipp'}$, $C_5Me_5{}^n$Bu, CH_{3nBu}), 0.32 (m, 2H, CH_{2nBu}), 0.25 (s, 3H, $C_5Me_5{}^n$Bu).

$^{13}C\{^1H\}$ NMR (125 MHz, CD_2Cl_2, −20 ºC) δ: 161.8 (q, $^1J_{CB}$ = 50 Hz, *ipso*-Ar), 147.0 (*o*-Dipp), 146.6 (*o*-Dipp), 146.4 (d, $^2J_{CP}$ = 27 Hz, *o*-C_6H_3), 144.3 (*o*-C_6H_3), 142.5 (*o*-Dipp'), 137.8 (d, $^2J_{CP}$ = 53 Hz, *ipso*-C_6H_3), 135.8 (*ipso*-Dipp), 134.8 (*o*-Ar), 133.9 (d, $^3J_{CP}$ = 5 Hz, *m*-C_6H_3), 133.8 (*m*-Dipp'), 132.5 (m, *m*-Dipp', *o*-Dipp'), 131.9 (d, $^3J_{CP}$ = 13 Hz, *m*-C_6H_3), 131.6 (*o*-Dipp), 131.1 (*p*-C_6H_3), 129.8 (*p*-Dipp), 128.8 (q, $^2J_{CF}$ = 32 Hz, *m*-Ar), 127.7 (*p*-Dipp'), 124.1 (m, CF_3), 123.0 (*m*-Dipp) , 121.6 (d, $^2J_{CP}$ = 15 Hz, $C_5Me_5{}^n$Bu), 120.2 (d, $^2J_{CP}$ = 4 Hz, *ipso*-Dipp'), 117.6 (m, *p*-Ar), 115.7 (d, $^2J_{CP}$ = 4 Hz, $C_5Me_5{}^n$Bu), 79.6 ($C_5Me_5{}^n$Bu), 61.0 ($C_5Me_5{}^n$Bu), 60.7 ($C_5Me_5{}^n$Bu), 37.5 (CH_2), 34.3 (($CHMe_2)_{Dipp'}$, ($CHMe_2)_{Dipp'}$), 31.5 (($CHMe_2)_{Dipp}$), 31.3 (($CHMe_2)_{Dipp}$), 27.4 (CH_2), 26.2 (Me_{Dipp}), 25.7 (Me_{Dipp}), 24.7 (Me_{Dipp}), 24.3 ($C_5Me_5{}^n$Bu), 24.1 (Me_{Dipp}, Me_{Dipp}), 23.0 (Me_{Dipp}), 22.6 (CH_2), 22.3 (CH_3), 22.1 (Me_{Dipp}), 21.2 (Me_{Dipp}), 21.0 ($C_5Me_5{}^n$Bu), 15.8 (d, $^1J_{CP}$ = 39 Hz, PMe*Me*), 15.3 (d, $^1J_{CP}$ = 38 Hz, P*Me*Me), 12.1 ($C_5Me_5{}^n$Bu), 11.0 ($C_5Me_5{}^n$Bu), 8.6 ($C_5Me_5{}^n$Bu).

$^{31}P\{^1H\}$ NMR (202 MHz, CD_2Cl_2, 25ºC) δ: 4.7.

2.2 Preparation of complexes 4 and 5

To a Et_2O solution (10 mL) of complex **1** (100.0 mg, 0.060 mmol) was added 1.1 equivalents of RMgBr (R=Me or Et) at room temperature. The resulting red solution was stirred for 20 h to ensure completion and then filtered. The solvent was evaporated under reduced pressure and the residue was washed with pentane (6 mL). Single crystals were grown from a saturated CH_2Cl_2-hexane solution at −32ºC.

4

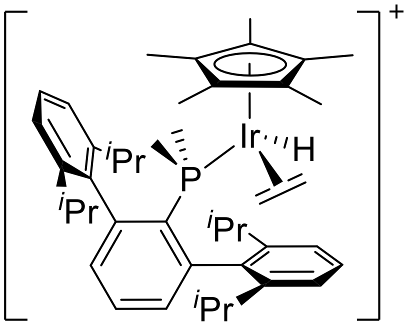

Yield: 31 mg (31 %).

Anal. Calcd. for $C_{76}H_{75}BF_{24}IrP$: C, 54.39; H, 4.50. **Found:** C, 54.40; H, 4.46.

^{1}H NMR (400 MHz, CD_2Cl_2, 25 ºC) δ: 7.71 (s, 8H, *o*-Ar), 7.56 (s, 4H, *p*-Ar), 7.50 (td, 1H, $^3J_{HH}$ = 7.6 Hz, $^5J_{HP}$ = 2.1 Hz, *p*-C_6H_3), 7.46 (t, 2H, $^3J_{HH}$ = 7.8 Hz, *p*-Dipp), 7.28 (m, 6H, *m*-Dipp, *m*-C_6H_3), 2.55 (m, 4H, (C*H*Me_2)$_{Dipp}$), 2.19 (d, 2H, $^2J_{HP}$ = 6.7 Hz, C*H*HC*H*H), 1.88 (d, 2H, $^2J_{HP}$ = 6.7 Hz, CH*H*CH*H*), 1.78 (d, 3H, $^2J_{HP}$ = 11.1 Hz, P*Me*Me), 1.62 (s, 15H, C_5Me_5), 1.37 (m, 12H, Me_{Dipp}), 1.30 (d, 3H, $^2J_{HP}$ = 11.1 Hz, PMe*Me*), 0.97 (m, 12H, Me_{Dipp}), −14.9 (d, 1H, $^2J_{HP}$ = 30.2 Hz, IrH).

^{13}C{^{1}H} NMR (100 MHz, CD_2Cl_2, 25 ºC) δ: 162.1 (q, $^1J_{CB}$ = 50 Hz, i*pso*-Ar), 147.5 (*o*-Dipp), 144.9 (d, $^2J_{CP}$ = 10 Hz, *o*-C_6H_3), 137.1 (d, $^3J_{CP}$ = 5 Hz, *ipso*-Dipp), 134.8 (*o*-Ar), 133.2 (d, $^3J_{CP}$ = 9 Hz, *m*-C_6H_3), 132.7 (d, $^1J_{CP}$ = 57 Hz, *ipso*-C_6H_3), 130.6 (*p*-Dipp), 129.9 (d, $^4J_{CP}$ = 3 Hz, *p*-C_6H_3), 129.3 (q, $^2J_{CF}$ = 31 Hz, m-Ar), 125.0 (q, $^1J_{CF}$ = 272 Hz, CF_3), 117.5 (m, *p*-Ar), 95.7 (d, $^2J_{CP}$ = 3 Hz, *C*$_5Me_5$), 33.8 (CH_2CH_2), 31.5 ((*C*HMe_2)$_{Dipp}$), 25.9 (v br, Me_{Dipp}), 22.5 (d, $^1J_{CP}$ = 39 Hz, P*Me*Me), 21.8 (br, Me_{Dipp}), 20.6 (d, $^1J_{CP}$ = 39 Hz, PMe*Me*), 9.2 (C_5*Me*$_5$).

^{31}P{^{1}H} NMR (162 MHz, CD_2Cl_2, 25 ºC) δ: −27.0.

5

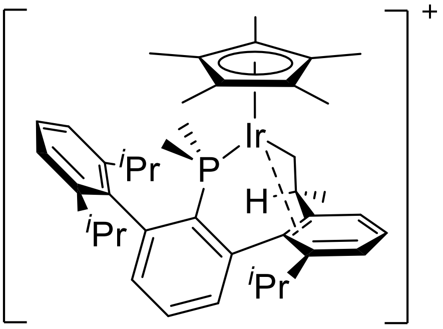

Yiel: 59 mg (60 %).

Anal. Calcd. for $C_{74}H_{69}BF_{24}IrP$: C, 53.92; H, 4.22. **Found:** C, 53.80; H, 4.19.

^{1}H NMR (500 MHz, CD_2Cl_2, 25 ºC) δ: 7.72 (s, 8H, *o*-Ar), 7.56 (s, 4H, *p*-Ar), 7.53 (m, 1H, *p*-Dipp'), 7.48 (t, $^{3}J_{HH}$ = 7.8 Hz, 1H, *p*-Dipp), 7.43 (td, $^{3}J_{HH}$ = 7.7 Hz, $^{5}J_{HP}$ = 2.3 Hz, 1H, *p*-C_6H_3), 7.34 (dd, $^{3}J_{HH}$ = 7.8 Hz, $^{4}J_{HH}$ = 1.0 Hz, 1H, *m*-Dipp), 7.29 (dd, $^{3}J_{HH}$ = 7.8 Hz, $^{4}J_{HH}$ = 1.0 Hz, 1H, *m*-Dipp), 7.19 (ddd, $^{3}J_{HH}$ = 7.7 Hz, $^{4}J_{HP}$ = 3.3 Hz, $^{4}J_{HH}$ = 1.1 Hz, 1H, *m*-C_6H_3), 7.14 (d, $^{3}J_{HH}$ = 8.5 Hz, 1H, *m*-Dipp'), 6.99 (m, 1H, *m*-Dipp'), 6.49 (ddd, $^{3}J_{HH}$ = 7.7 Hz, $^{4}J_{HP}$ = 2.5 Hz, $^{4}J_{HH}$ = 1.1 Hz, 1H, *m'*-C_6H_3),3.30 (m, 1H, C*H*CH_2Ir), 2.50 (m, 1H, (C*H*Me_2)$_{Dipp}$), 2.43 (m, 1H, (C*H*Me_2)$_{Dipp'}$), 2.33 (m, 1H, (C*H*Me_2)$_{Dipp'}$), 1.92 (d, $^{2}J_{HP}$ = 10.5 Hz, 3H, P*Me*Me), 1.36 (d, $^{3}J_{HH}$ = 6.7 Hz, 3H, Me_{Dipp}), 1.31 (d, $^{4}J_{HP}$ = 1.8 Hz, 15H, C_5Me_5), 1.24 (d, $^{3}J_{HH}$ = 6.7 Hz, 3H, Me_{Dipp}), 1.21 (m, 6H, $Me_{Dipp''}$, PMe*Me*), 1.07 (d, $^{3}J_{HH}$ = 6.7 Hz, 3H, Me_{Dipp}) 1.05 (d, $^{3}J_{HH}$ = 6.7 Hz, 3H, Me_{Dipp}), 1.02 (d, $^{3}J_{HH}$ = 6.7 Hz, 3H, $Me_{Dipp'}$) 1.00 (d, $^{3}J_{HH}$ = 6.7 Hz, 3H, $Me_{Dipp'}$), 0.69 (m, 1H, C*H*HIr), 0.18 (m, 1H, CH*H*Ir).

^{13}C{^{1}H} NMR (125 MHz, CD_2Cl_2, 25 ºC) δ: 162.1 (q, $^{1}J_{CB}$ = 50 Hz, i*pso*-Ar), 157.0 (*o*-Dipp), 147.9 (d, $^{2}J_{CP}$ = 27 Hz, *o*-C_6H_3), 147.7 (*o*-Dipp'), 146.9 (*o'*-Dipp'), 144.8 (d, $^{2}J_{CP}$ = 2 Hz, *o'*-C_6H_3), 138.3 (d, $^{2}J_{CP}$ = 59 Hz, *ipso*-C_6H_3), 136.0 (d, $^{3}J_{CP}$ = 2 Hz, *ipso*-Dipp), 135.2 (*o*-Ar), 133.5 (d, $^{3}J_{CP}$ = 6 Hz, *m*-C_6H_3), 132.1 (d, $^{4}J_{CP}$ = 2 Hz, *p*-C_6H_3), 130.4 (*p*-Dipp'), 130.3 (*p*-Dipp), 129.9 (d,$^{3}J_{CP}$ = 13 Hz, *m*-C_6H_3), 129.3 (q, $^{2}J_{CF}$ = 32 Hz, *m*-Ar), 127.2 (m, CF_3) 124.5 (*m*-Dipp'), 123.6 (*m*-Dipp, *m*-Dipp) , 120.1 (*m*-Dipp'), 117.9 (m, *p*-Ar), 102.7 (d, $^{2}J_{CP}$ = 4 Hz, *ipso*-Dipp'), 97.4 (d, $^{3}J_{CP}$ = 3 Hz, *C*$_5$Me$_5$), 85.6 (*o'*-Dipp), 37.7 ((*C*HCH_2Me)$_{Dipp}$, 32.9 (*C*HMe_2)$_{Dipp}$), 31.7 ((*C*HMe_2)$_{Dipp'}$), 31.4 ((*C*HMe_2)$_{Dipp'}$), 26.4 ($Me_{Dipp'}$), 26.3 ($Me_{Dipp'}$), 25.6 (Me_{Dipp}), 24.3 (Me_{Dipp}), 21.9 ($Me_{Dipp'}$), 21.7 ($Me_{Dipp'}$), 20.1 (CHCH_2*Me*)$_{Dipp}$), 17.6 (d, $^{1}J_{CP}$ = 36 Hz, PMe), 11.7 (d, $^{1}J_{CP}$ = 45 Hz, PMe), 8.6 (C_5*Me*$_5$), 10.98 (C_5*Me*$_5$^{n}Bu), 8.62 (C_5*Me*$_5$^{n}Bu), −23.4 (d, $^{2}J_{CP}$ = 8 Hz, CH*C*H_2Me)$_{Dipp}$).

^{31}P{^{1}H} NMR (202 MHz, CD_2Cl_2, 25 ºC) δ: 6.3

3. NMR Spectra of Reported Complexes

2·Me

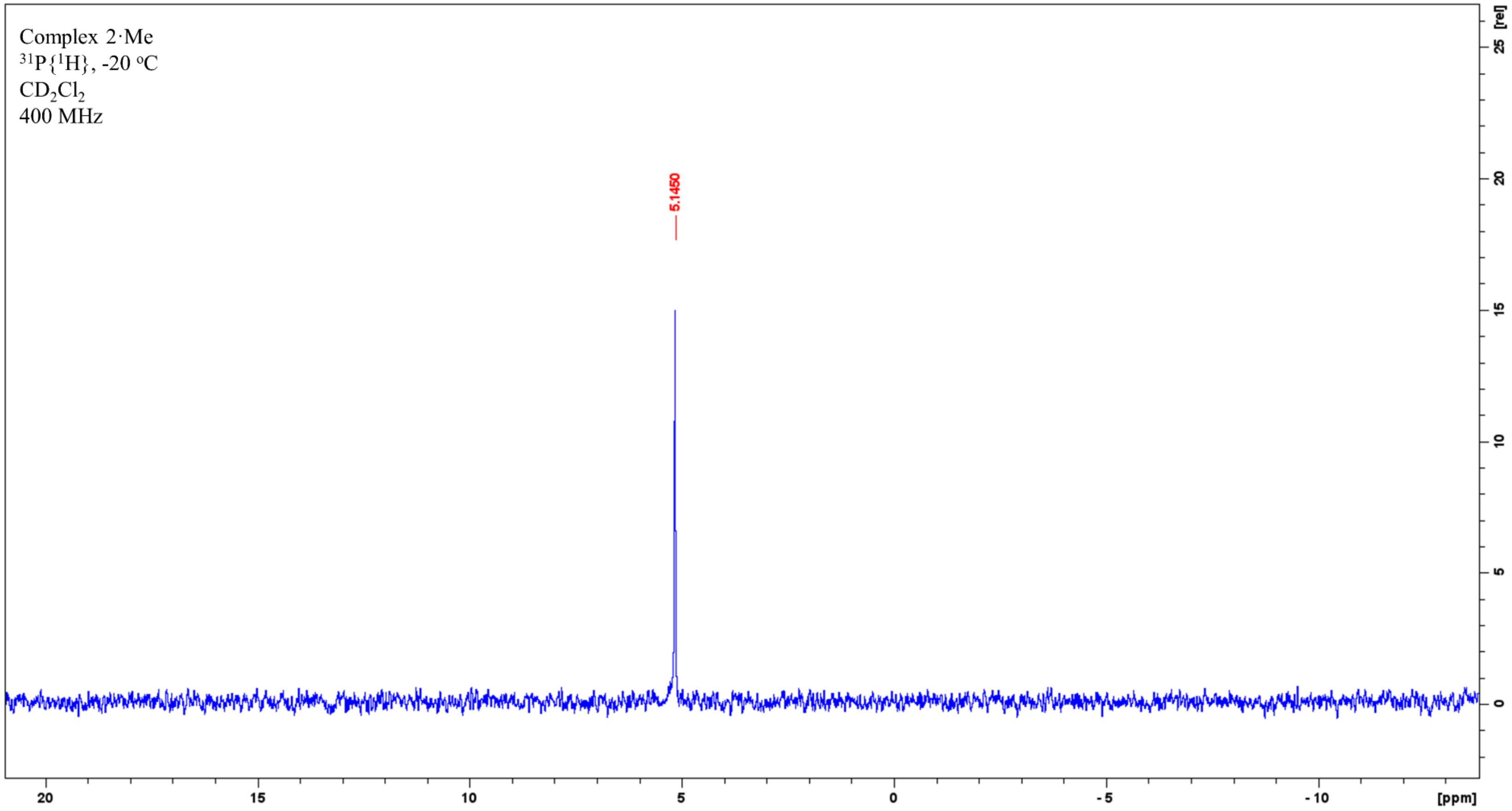

Figure S1. *$^{31}P\{^{1}H\}$ NMR (162 MHz, CD_2Cl_2, −20 °C). Complex **2·Me**.*

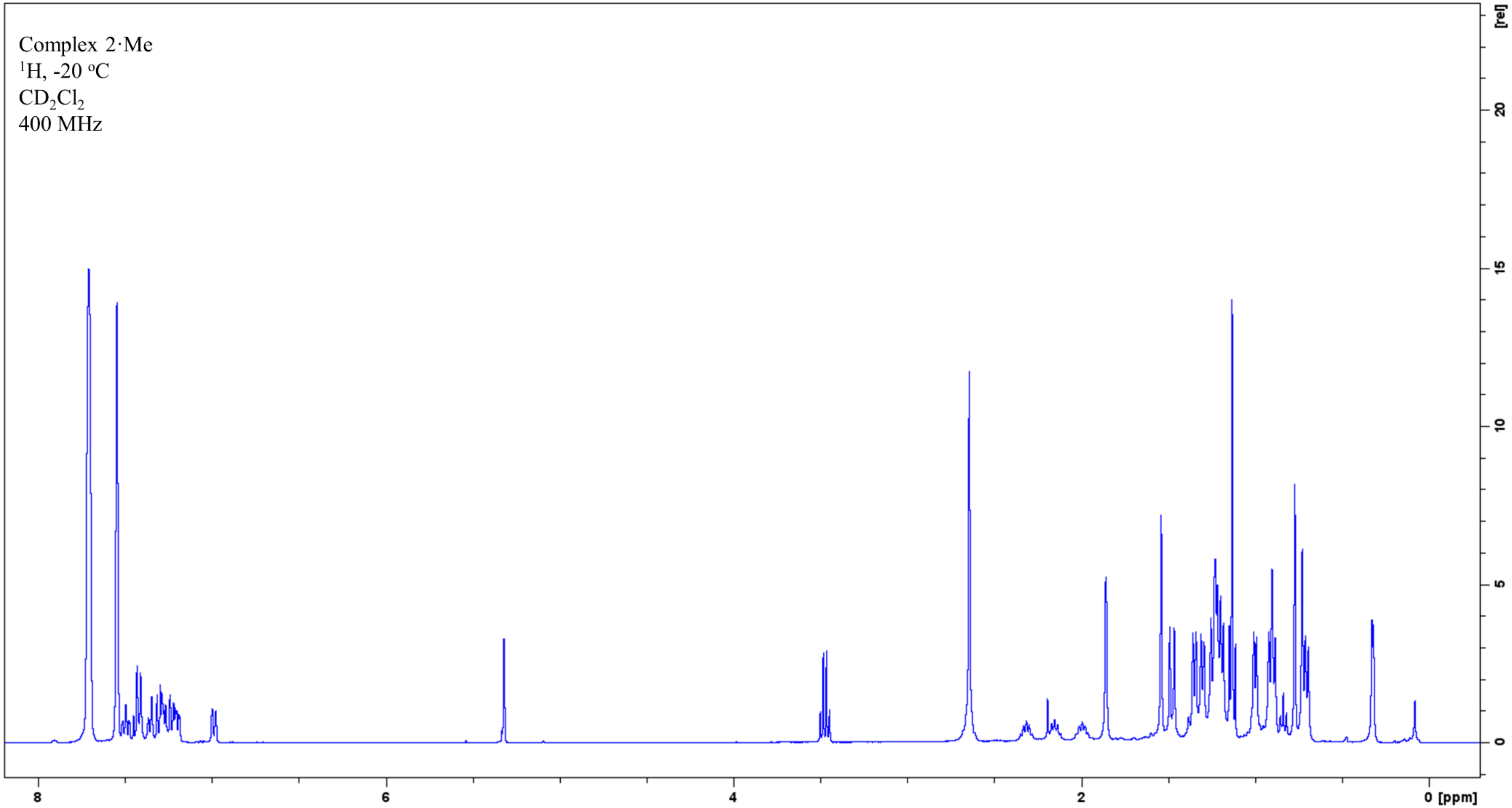

Figure S2. *1H NMR (400 MHz, CD_2Cl_2, −20 °C). Complex* ***2·Me****.*

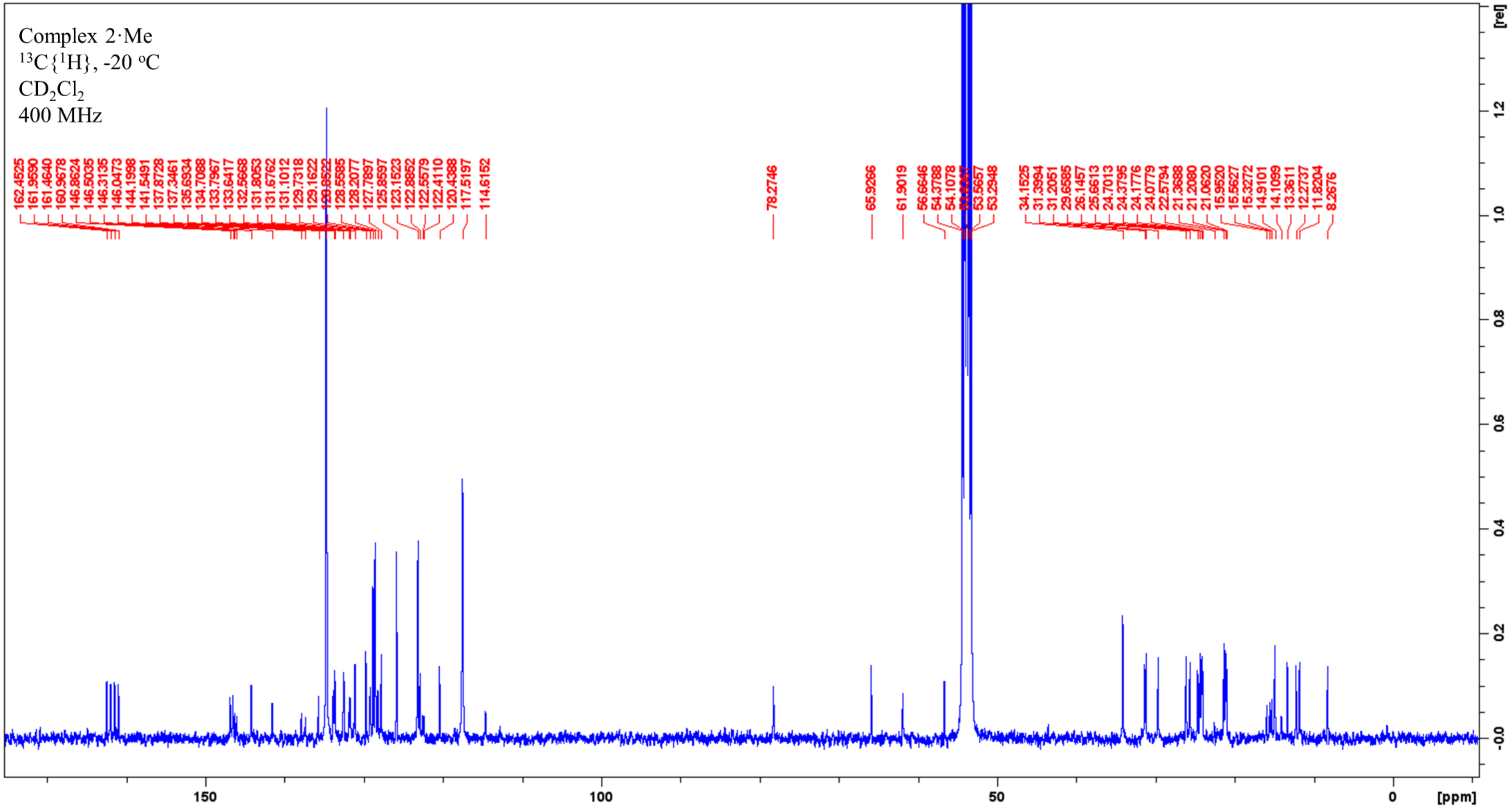

Figure S3. *$^{13}C\{^{1}H\}$ NMR (100 MHz, CD_2Cl_2, −20 °C). Complex **2·Me**.*

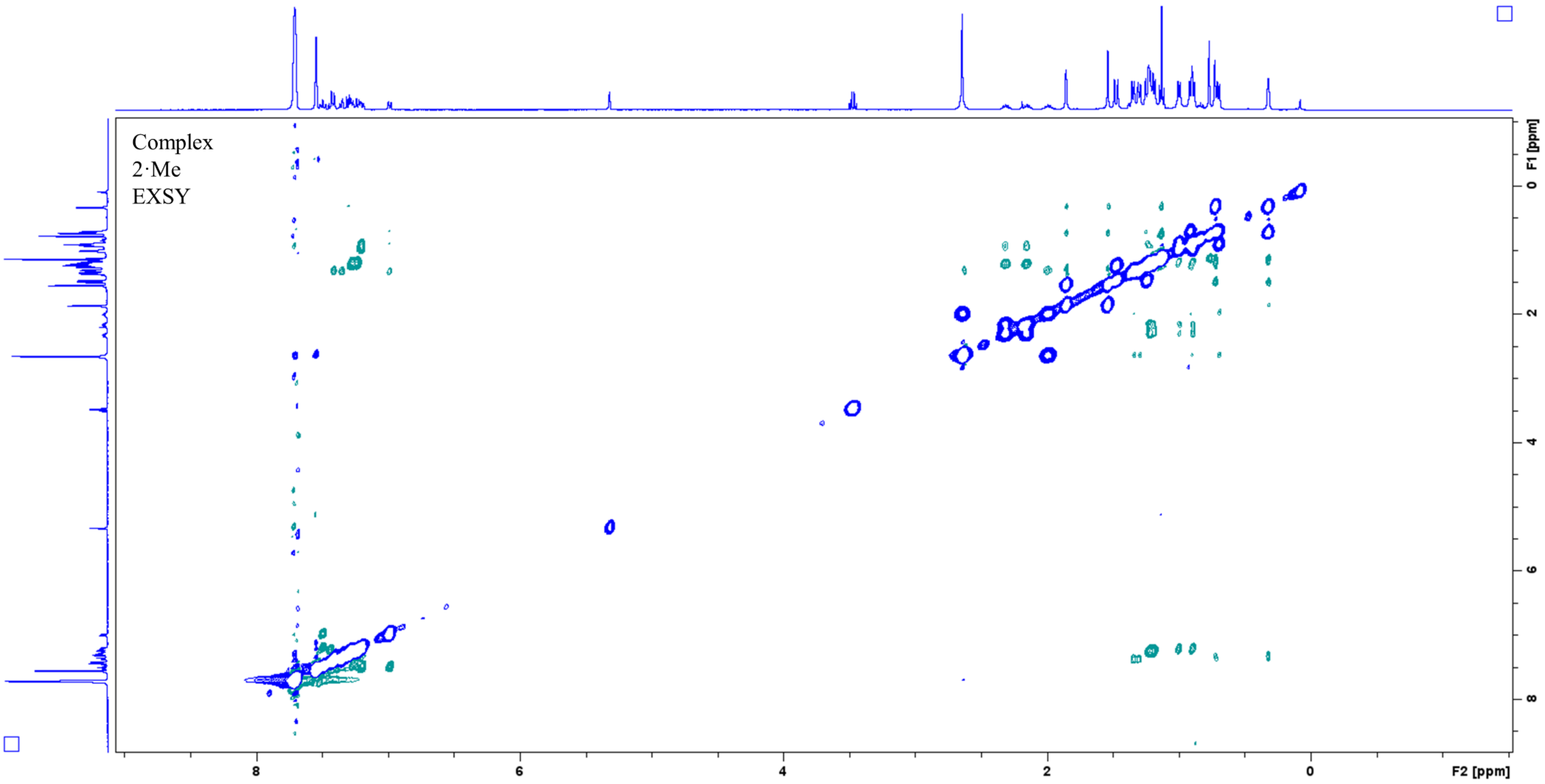

Figure S4. *EXSY, complex* ***2·Me***

2·Et

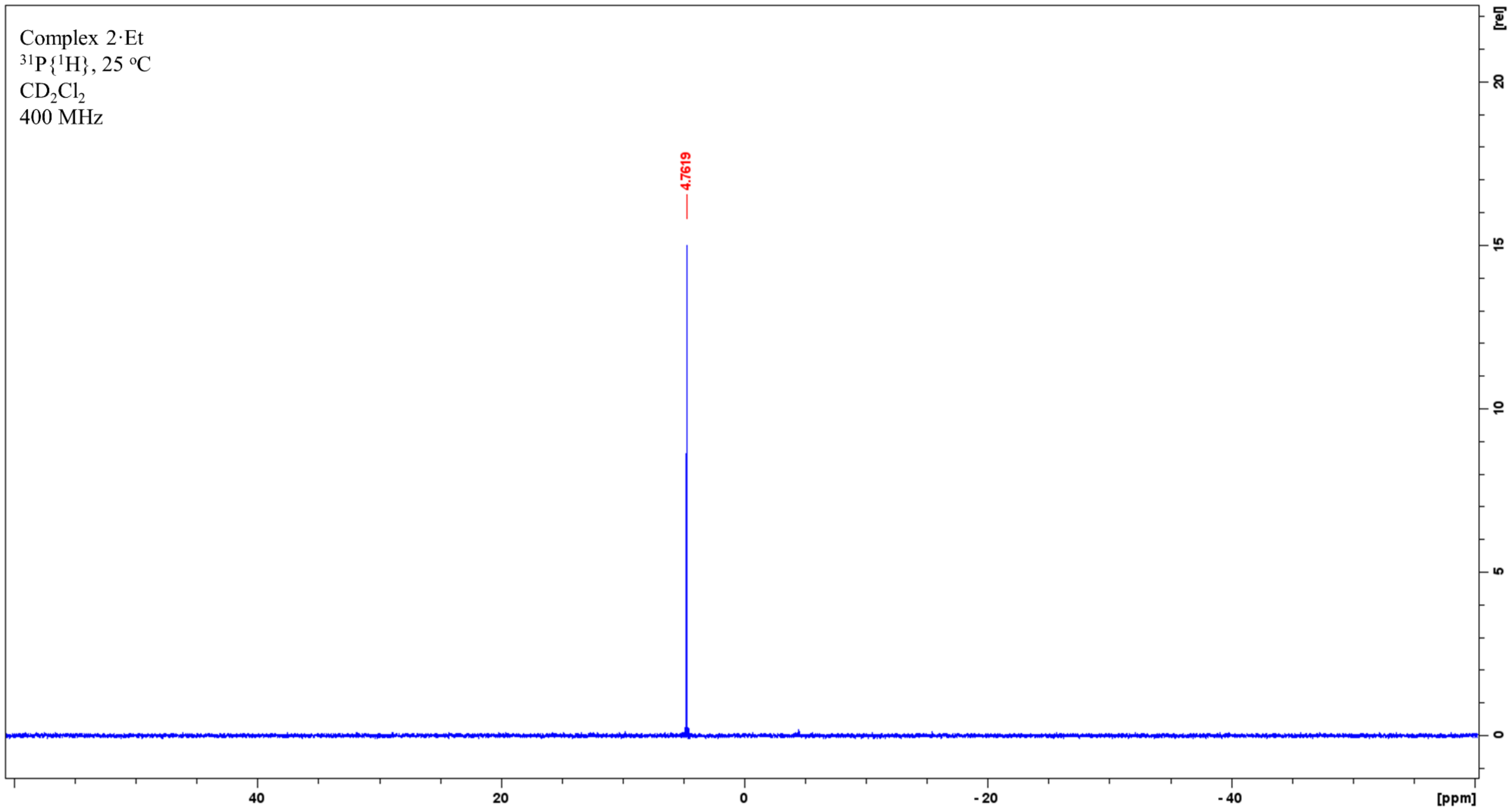

Figure S5. *$^{31}P\{^1H\}$ NMR (162 MHz, CD_2Cl_2, 25 °C). Complex **2·Et**.*

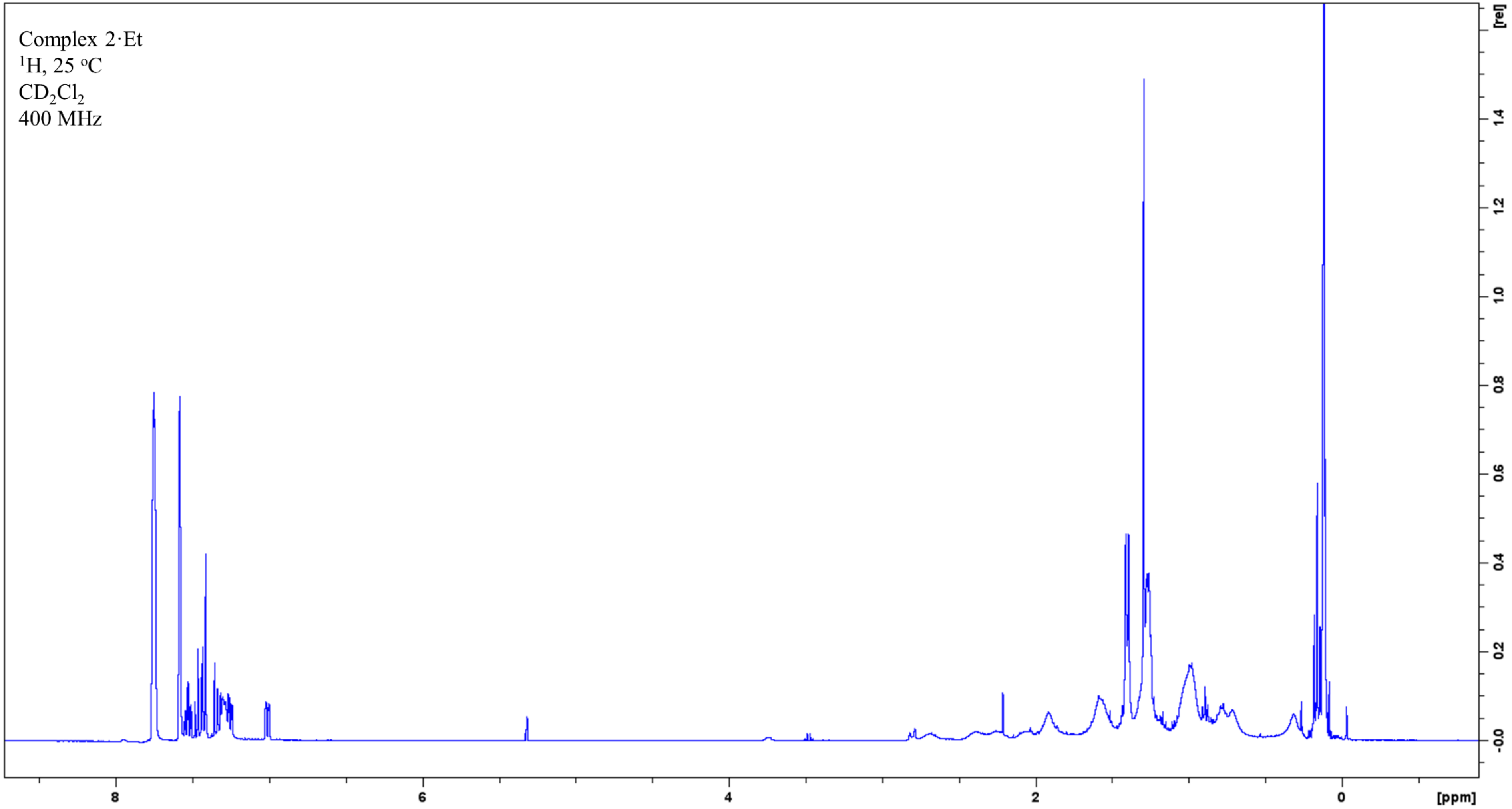

Figure S6. *^{1}H NMR (400 MHz, CD_2Cl_2, 25 °C). Complex* ***2·Et***.

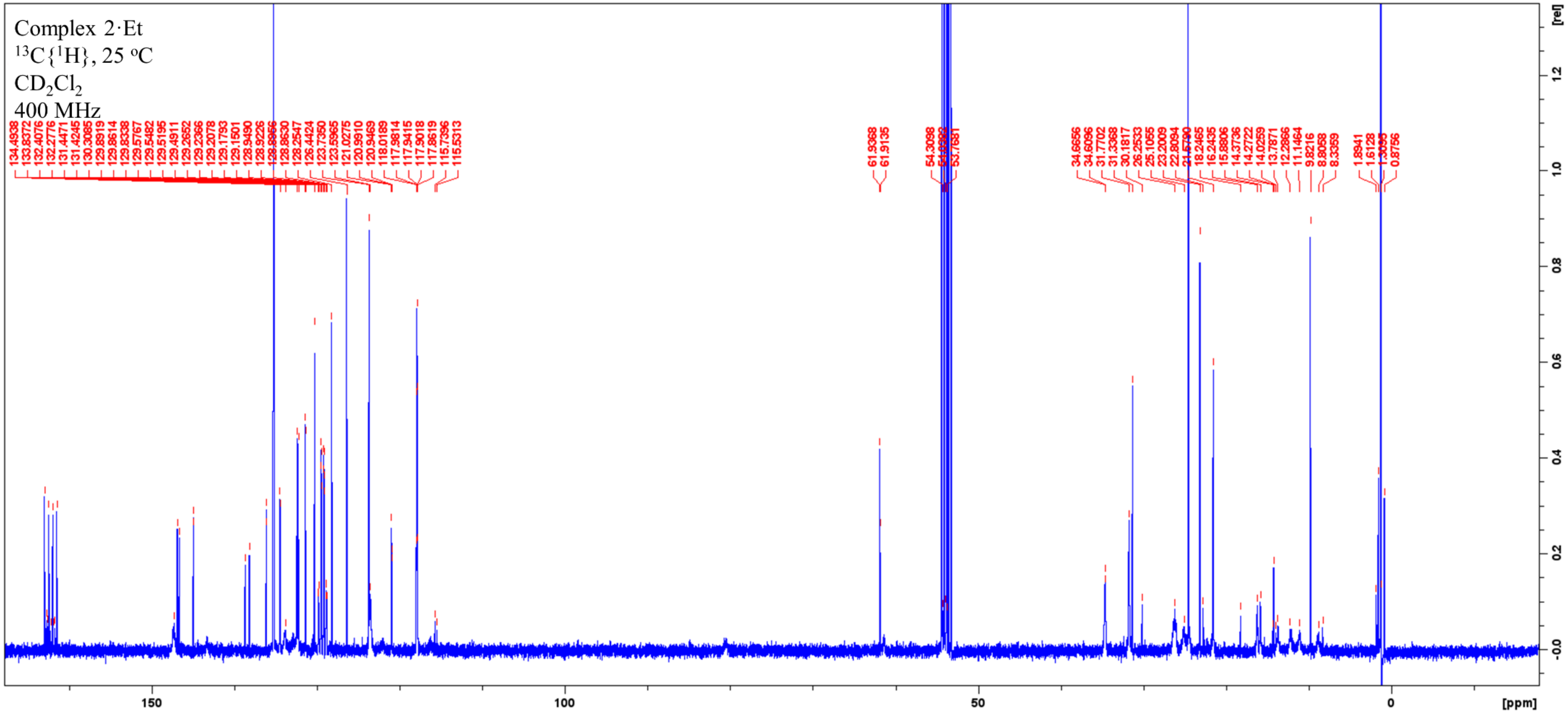

Figure S7. *$^{13}C\{^{1}H\}$ NMR (100 MHz, CD_2Cl_2, 25 °C). Complex* ***2·Et***.

2·^{i}Pr

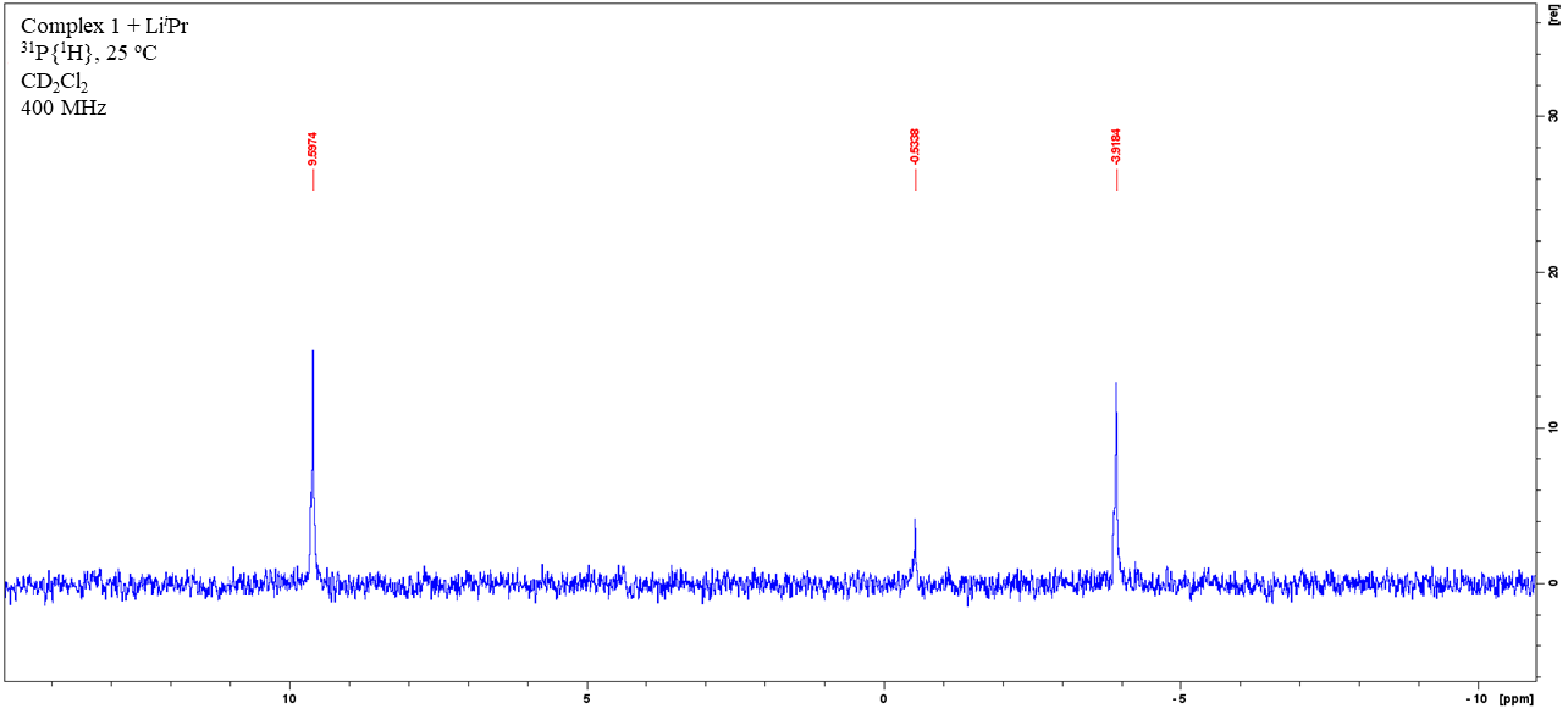

Figure S8. *$^{31}P\{^{1}H\}$ NMR (162 MHz, CD_2Cl_2, −20 °C) for the reaction between complex* ***1*** *and Li^{i}Pr. The signal resonating at −0.5 ppm corresponds to complex* ***2·^{i}Pr****, the one at −3.9 ppm to the intermediate and the final product of the isomerisation, complex* ***3****, resonates at 9.6 ppm.*

2·ⁿBu

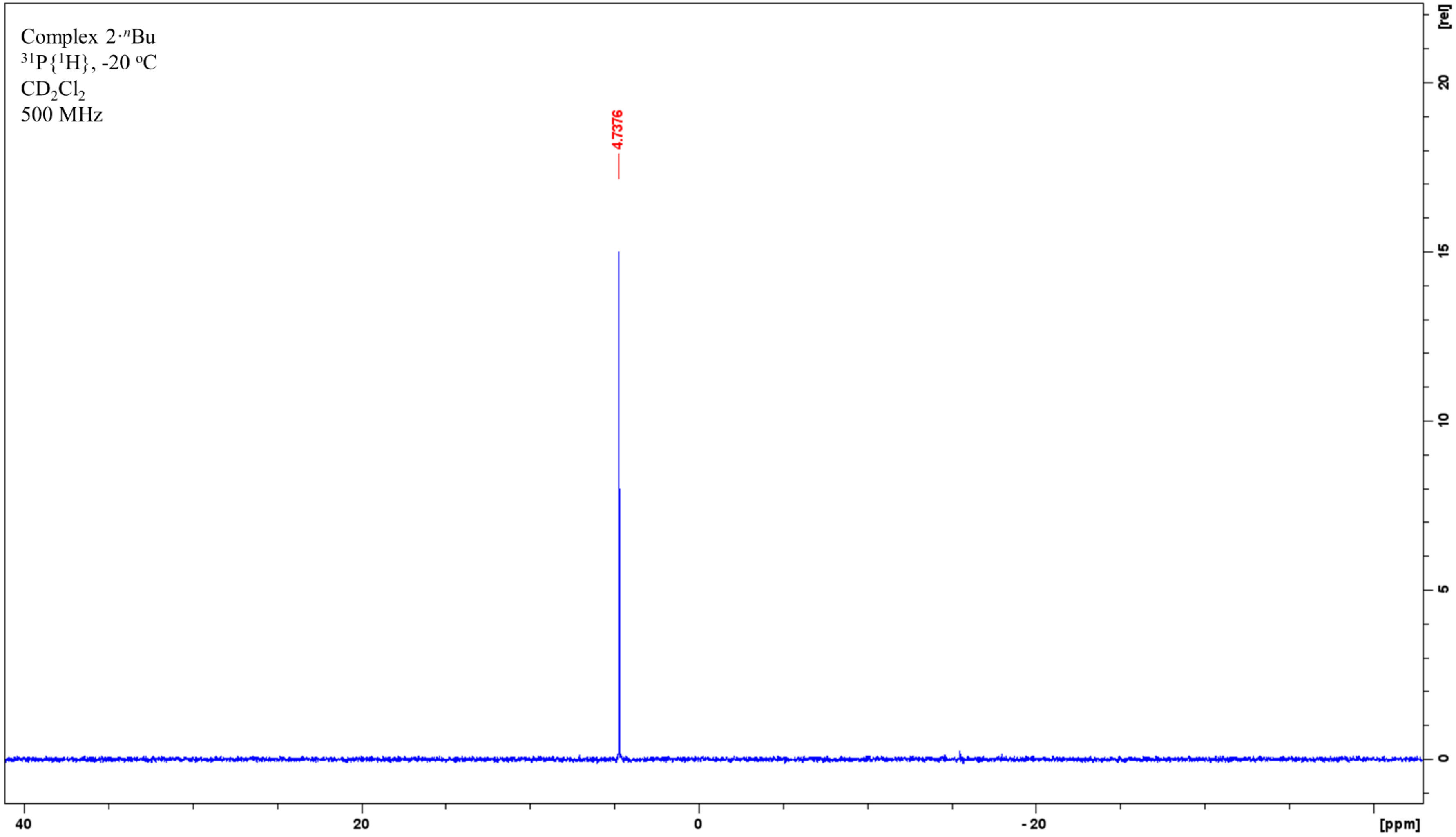

Figure S9. . *$^{31}P\{^1H\}$ NMR (202 MHz, CD_2Cl_2, −20 °C). Complex **2·^{n}Bu**.*

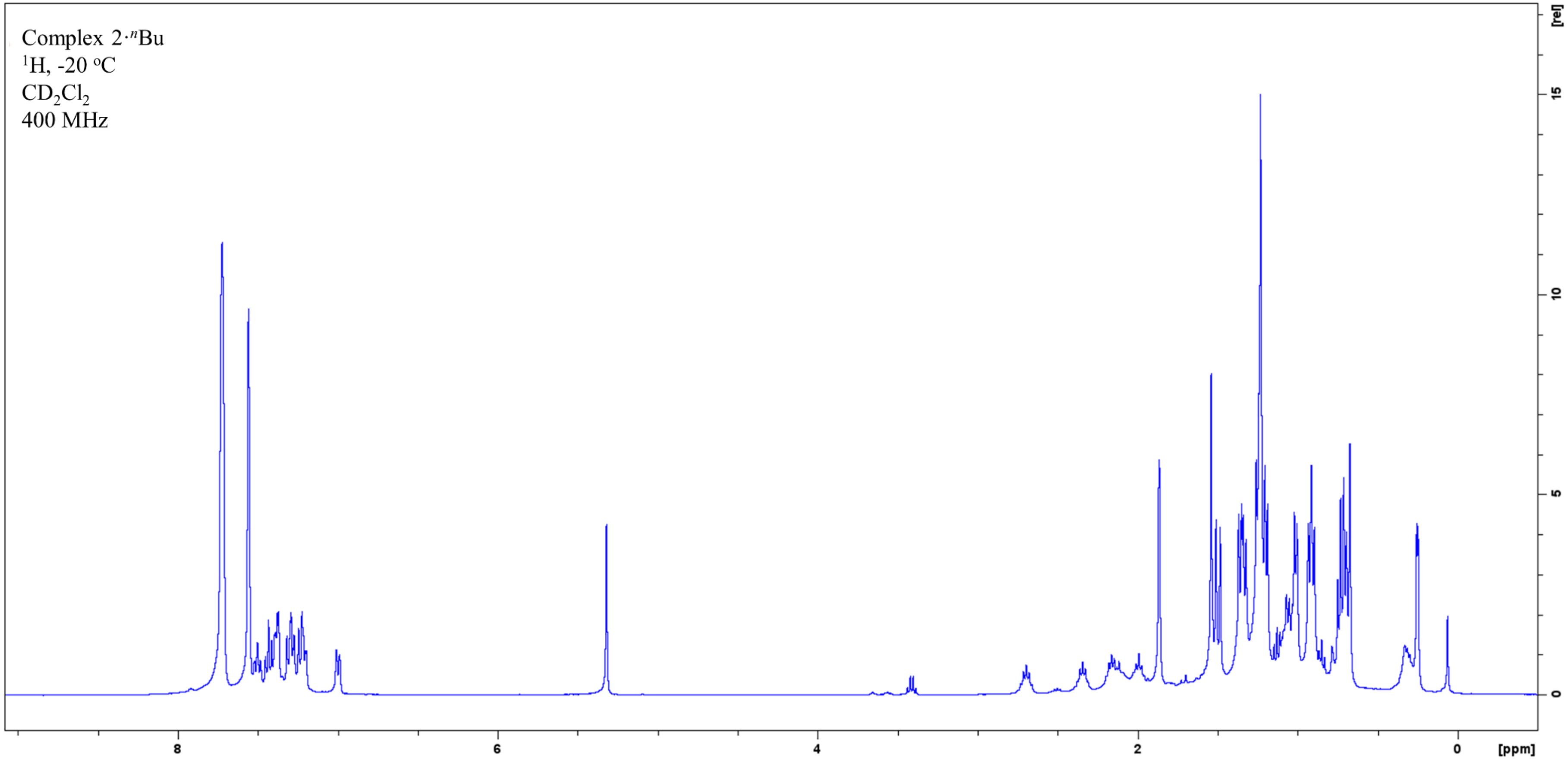

Figure S10. *^{1}H NMR (400 MHz, CD_2Cl_2, −20 °C). Complex* ***2·^{n}Bu***.

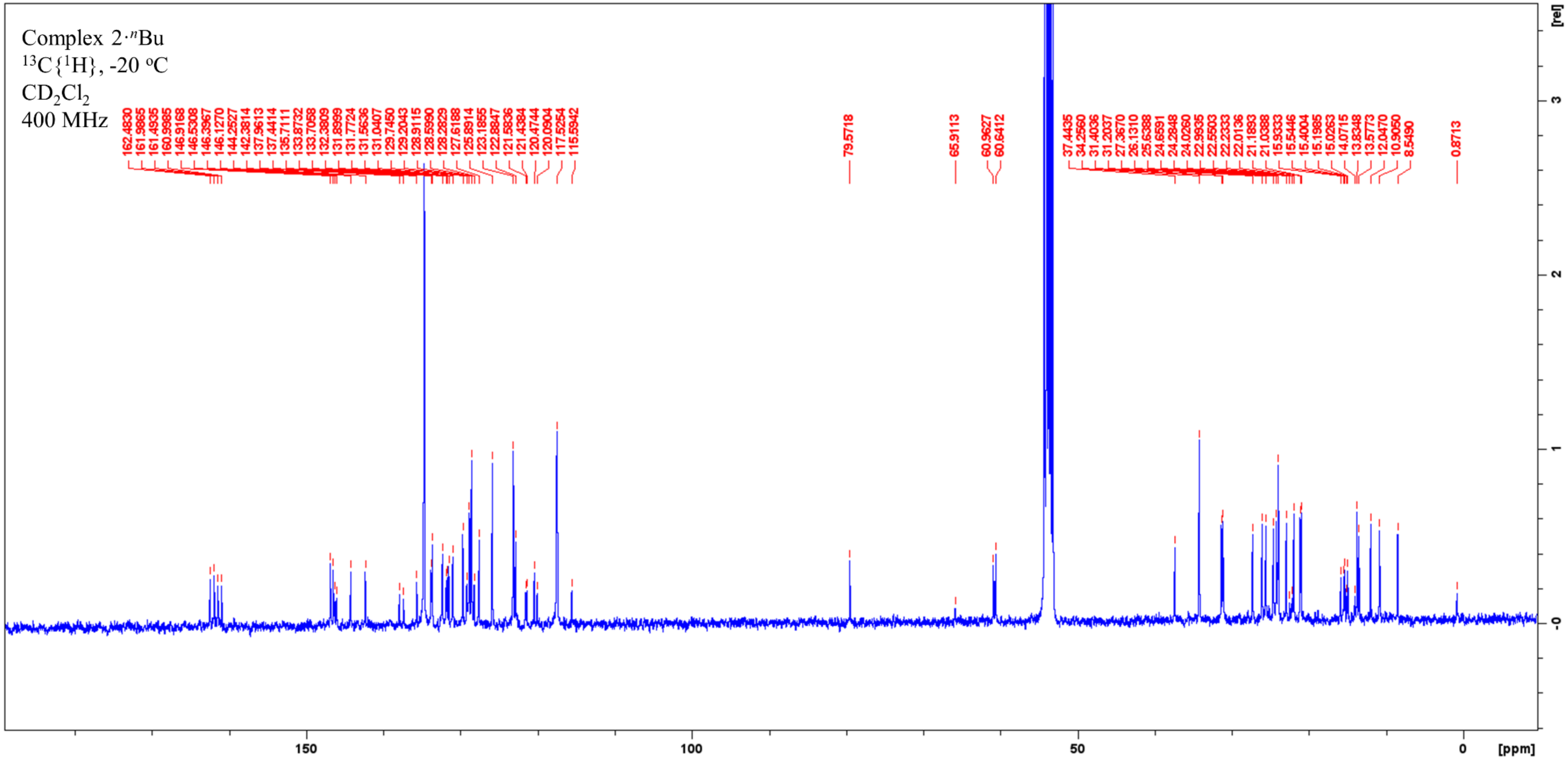

Figure S11. *$^{13}C\{^{1}H\}$ NMR (100 MHz, CD_2Cl_2, −20 °C). Complex* ***2·ⁿBu****.*

4

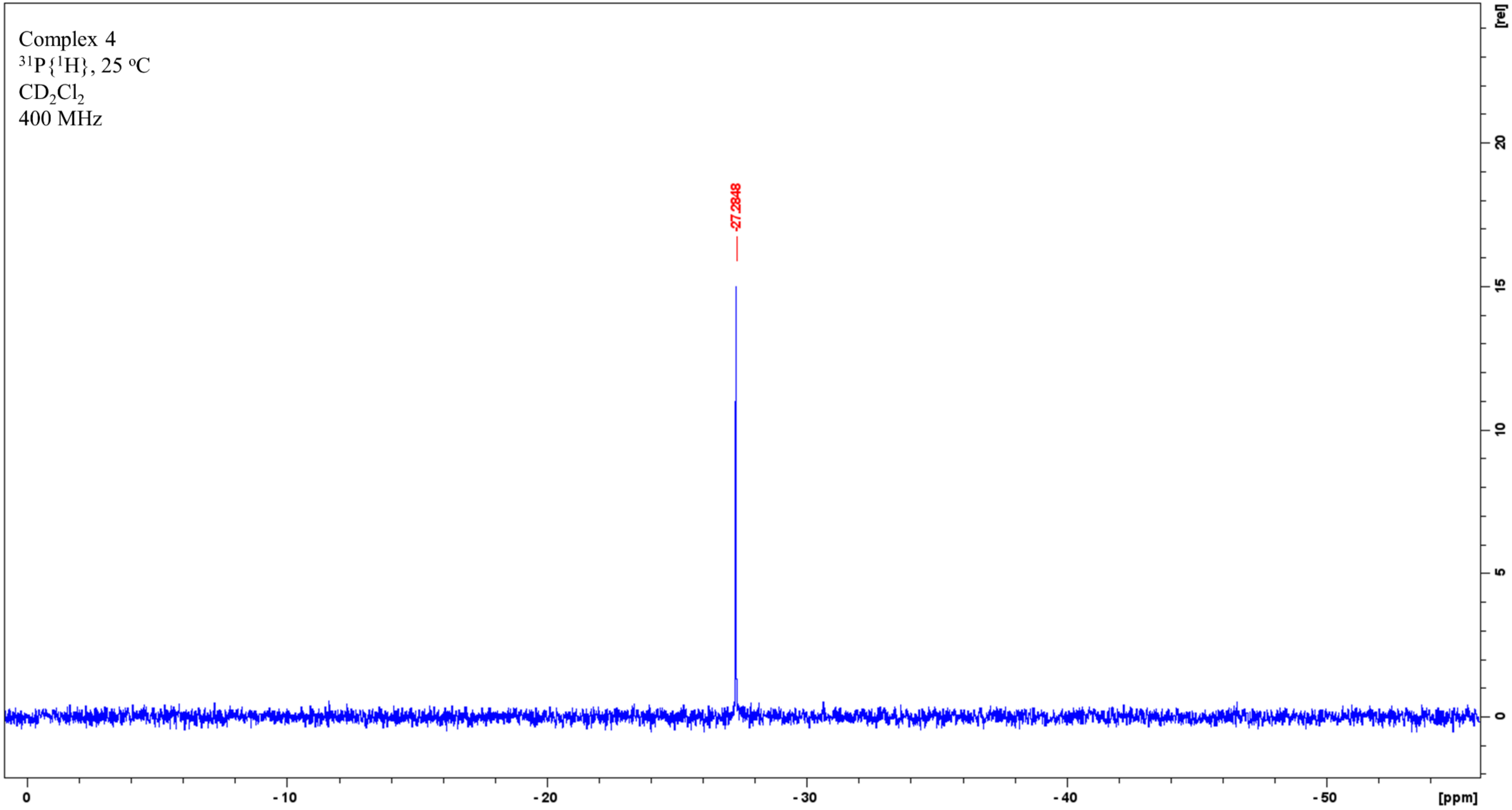

***Figure S12**. $^{31}P\{^{1}H\}$ NMR (162 MHz, CD_2Cl_2, −20 °C). Complex **4**.*

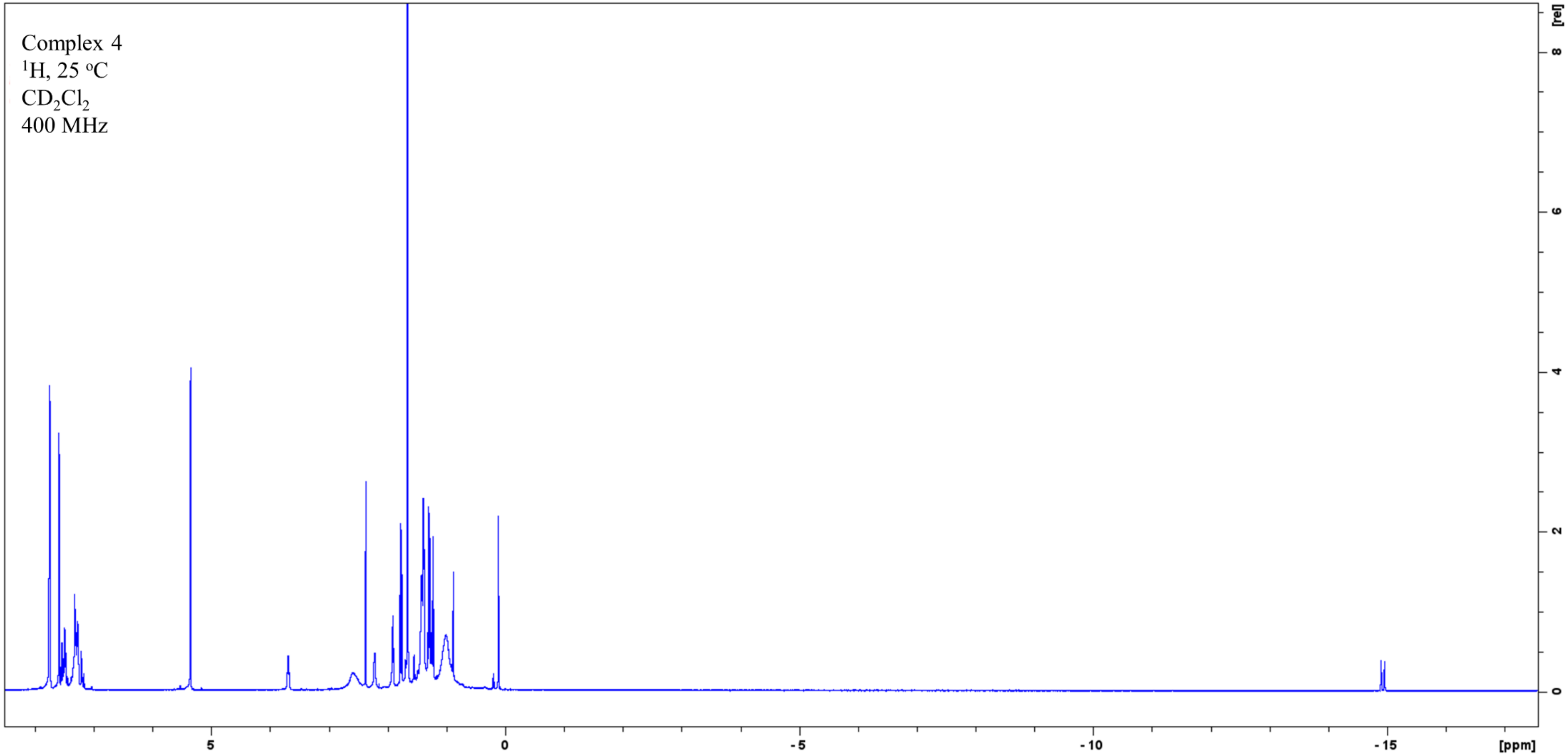

Figure S13. *^{1}H NMR (400 MHz, CD_2Cl_2, 25 °C). Complex* ***4***.

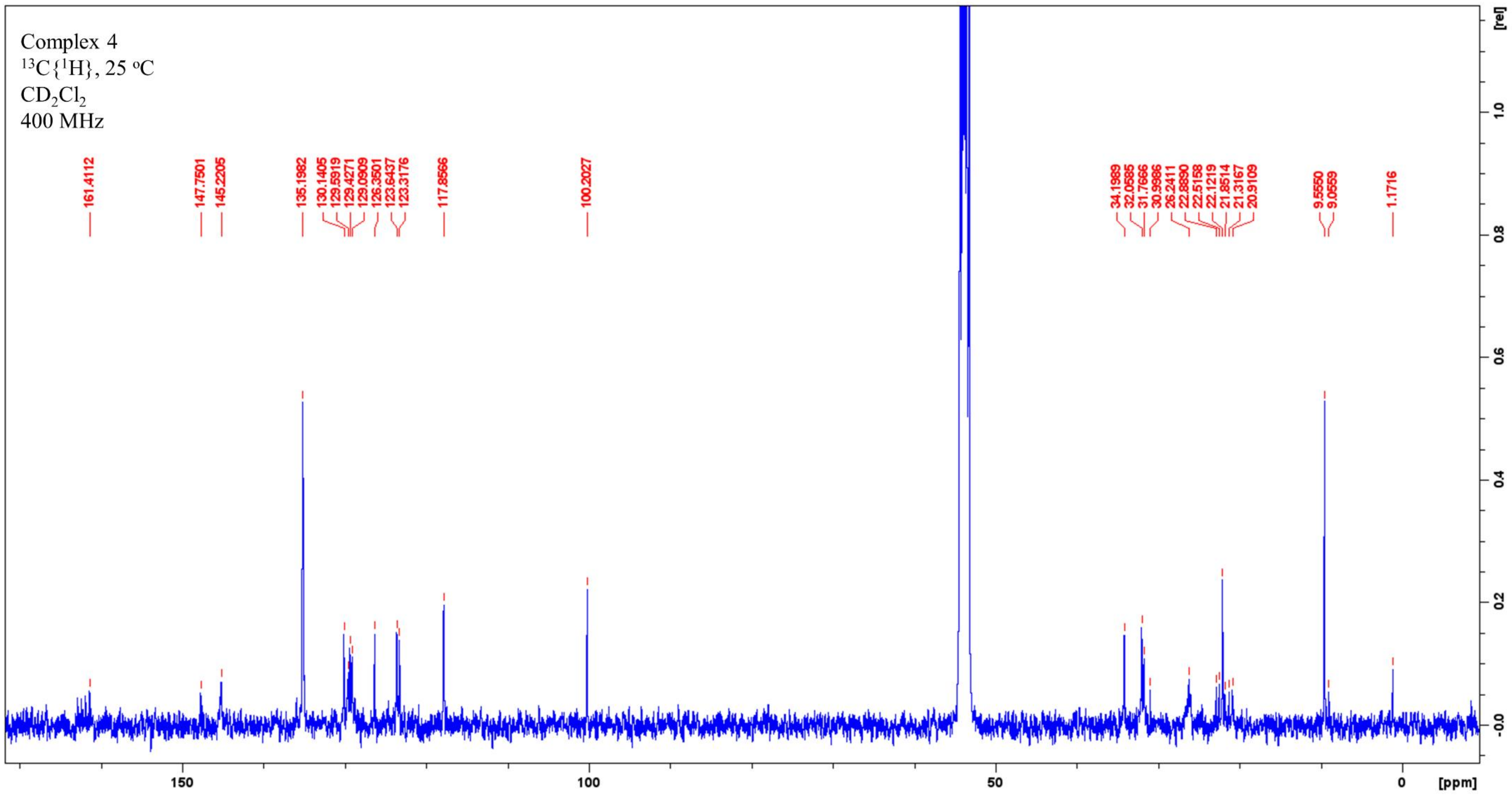

Figure S14. *$^{13}C\{^1H\}$ NMR (100 MHz, CD_2Cl_2, 25 °C). Complex **4**.*

5

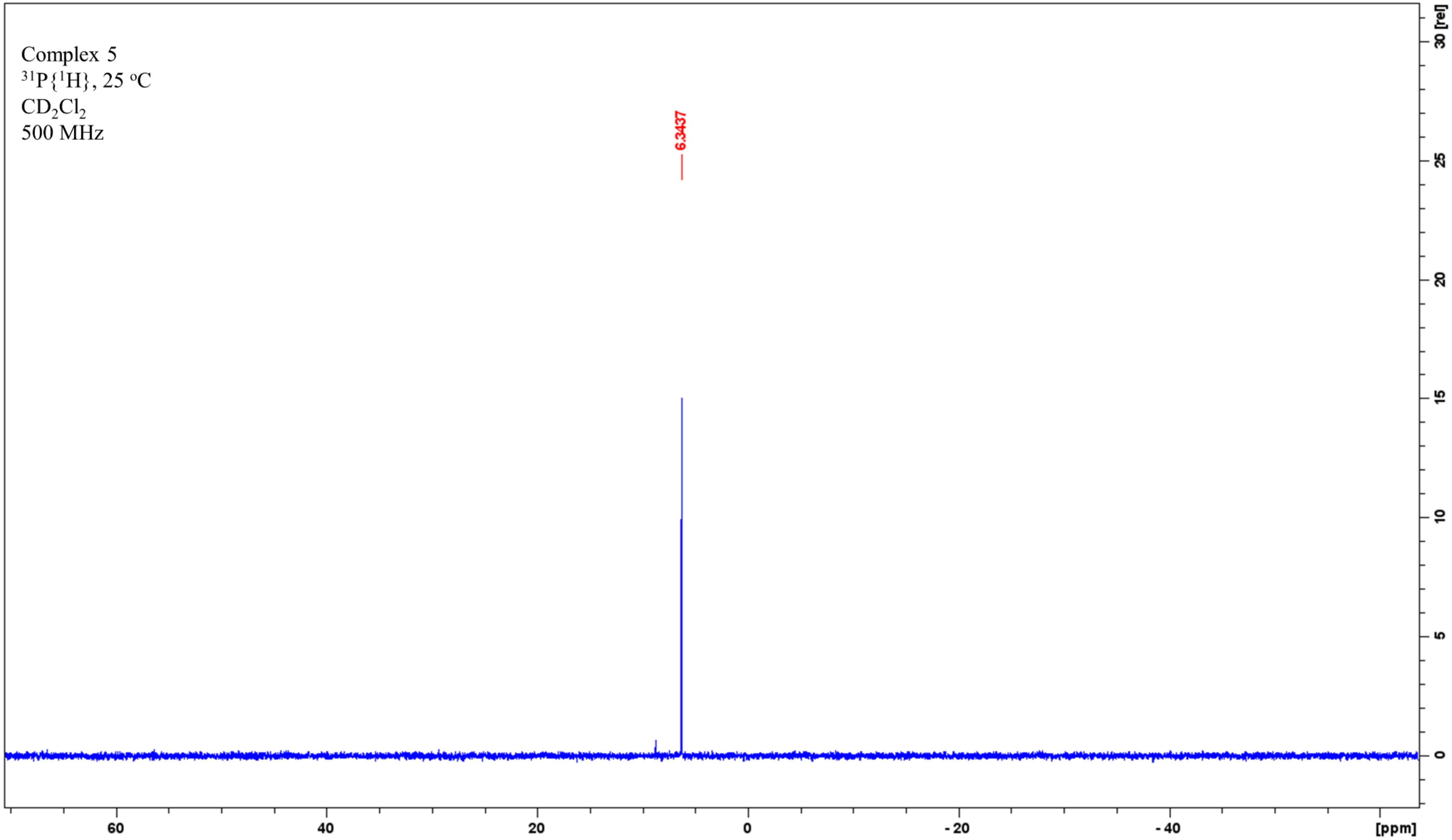

Figure S15. *$^{31}P\{^1H\}$ NMR (202 MHz, CD_2Cl_2, −20 °C). Complex* ***5***.

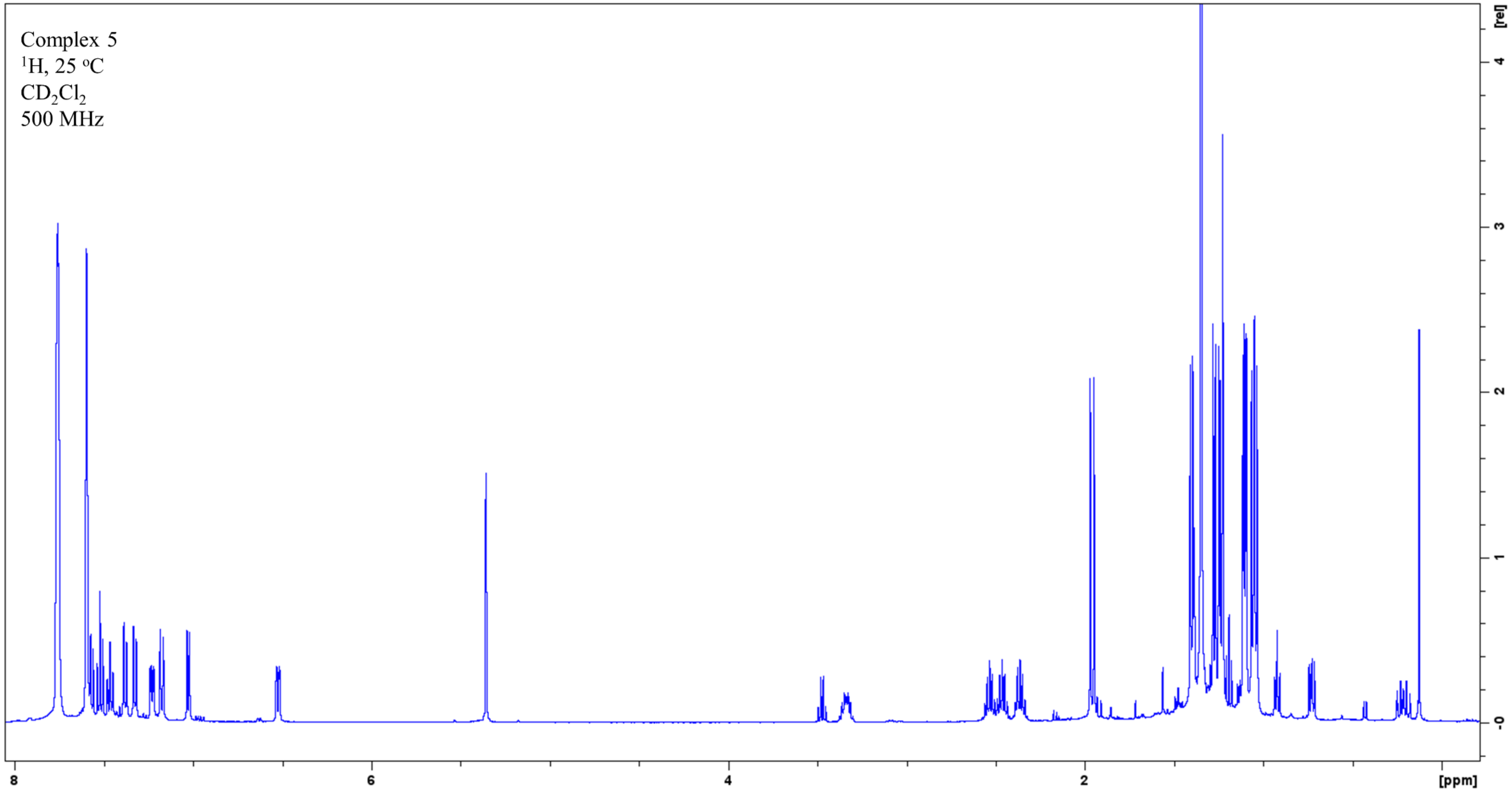

Figure S16. *^{1}H NMR (500 MHz, CD_2Cl_2, −20 °C). Complex* ***5***.

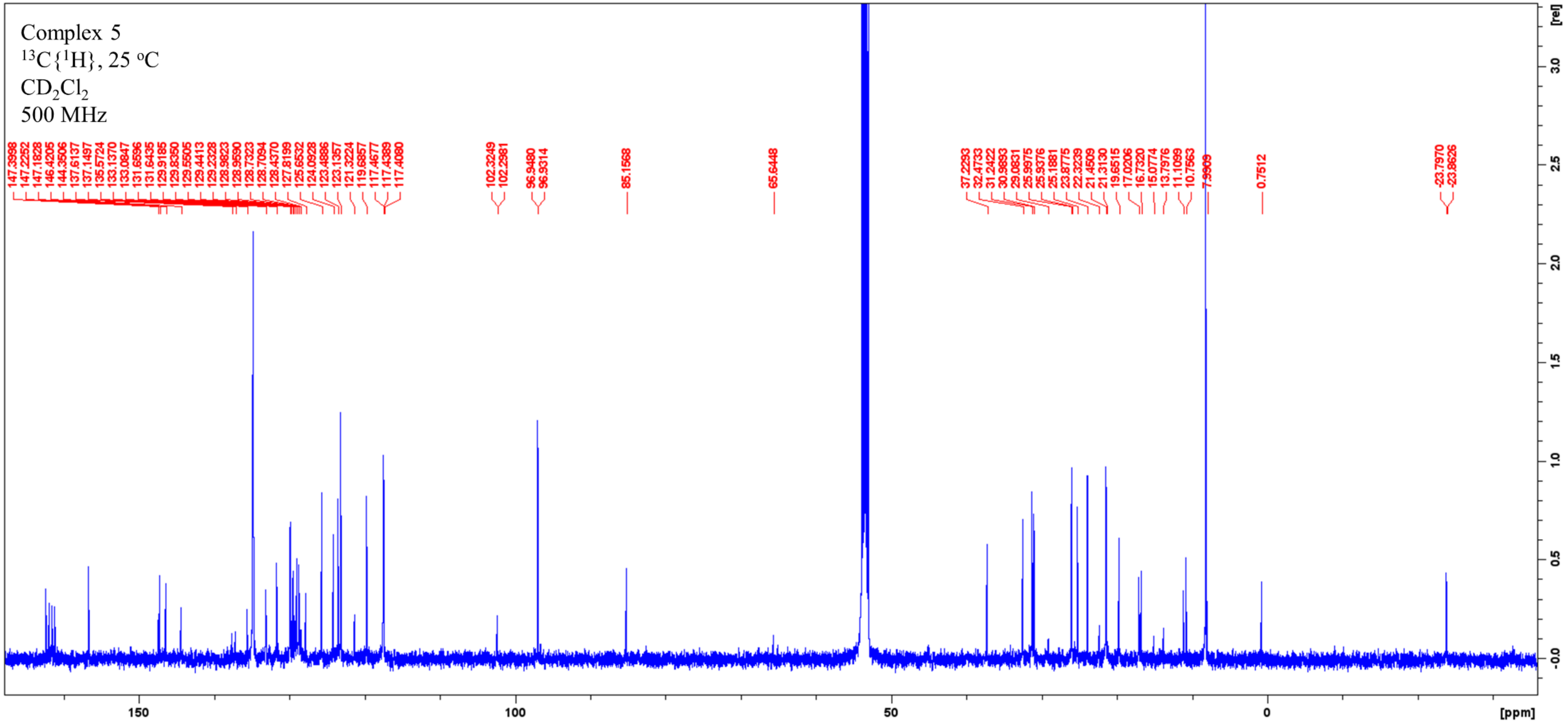

Figure S17. *$^{13}C\{^1H\}$ NMR (125 MHz, CD_2Cl_2, 25 °C). Complex **5**.*

5’

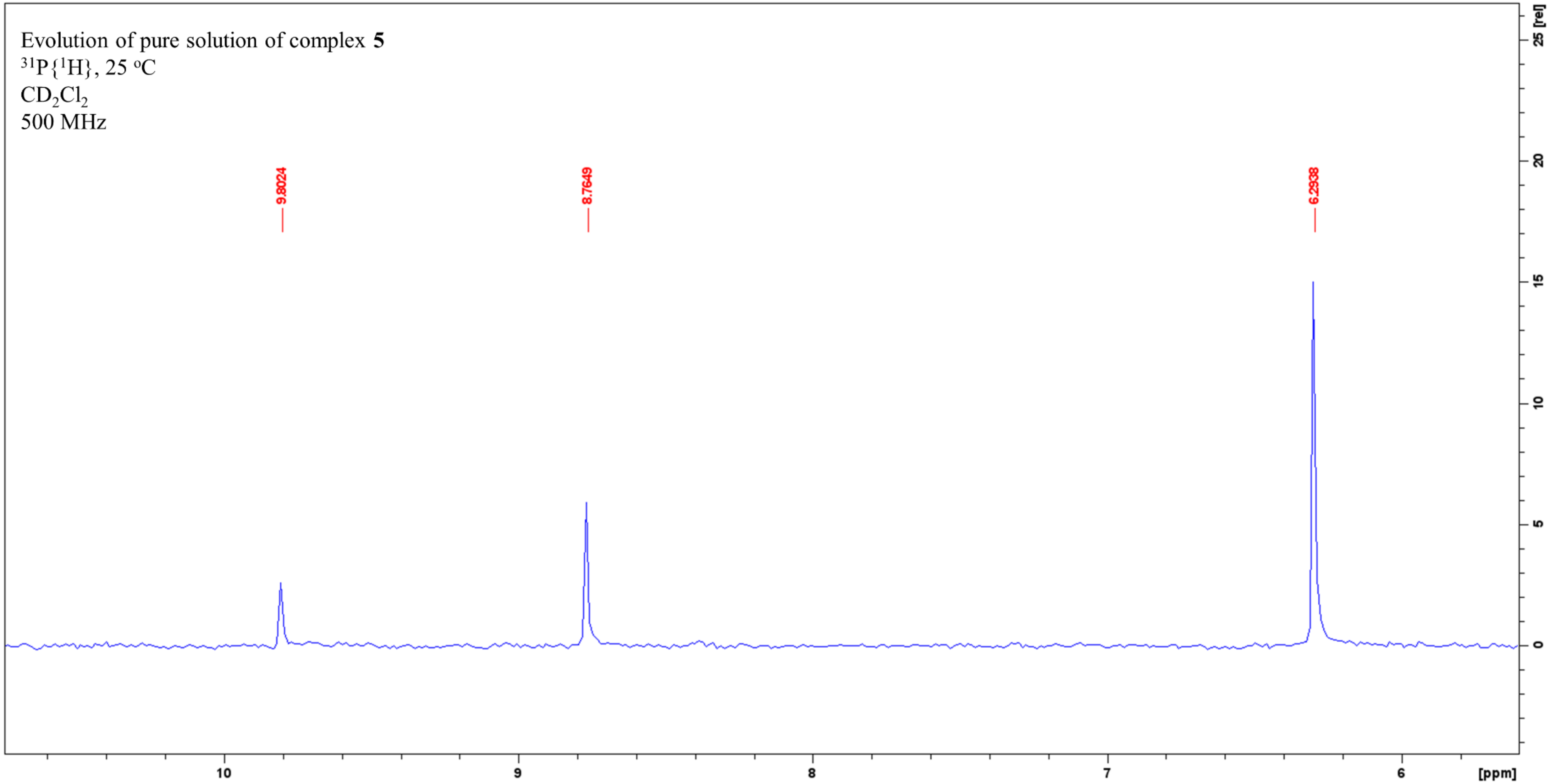

Figure S18. *$^{31}P\{^{1}H\}$ NMR (202 MHz, CD_2Cl_2, 25 °C) of a solution of complex* ***5*** *left at room temperature for a week.*

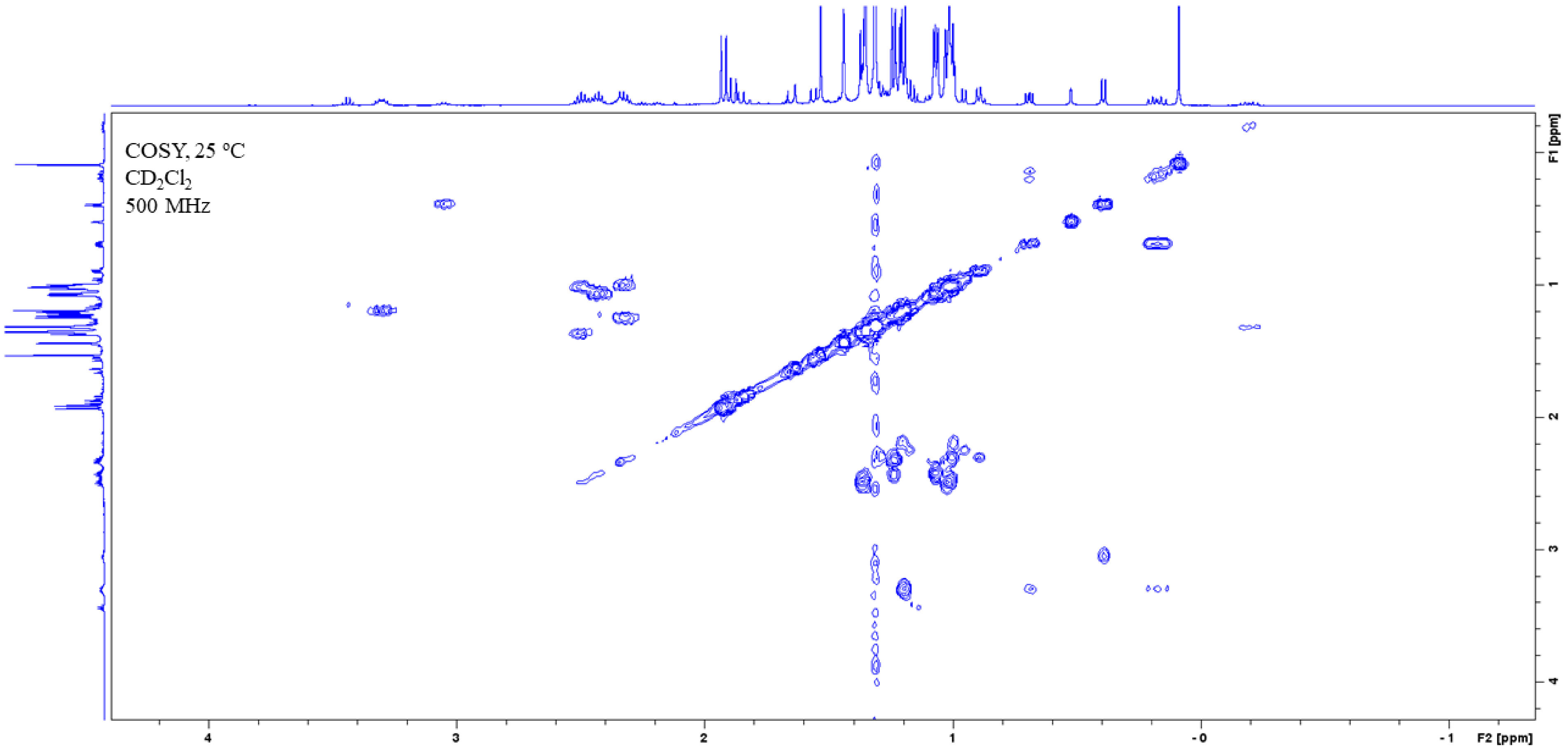

Figure S19. *COSY of the aliphatic region of a solution containing complexes* ***3***, ***5***, *and* ***5'***.

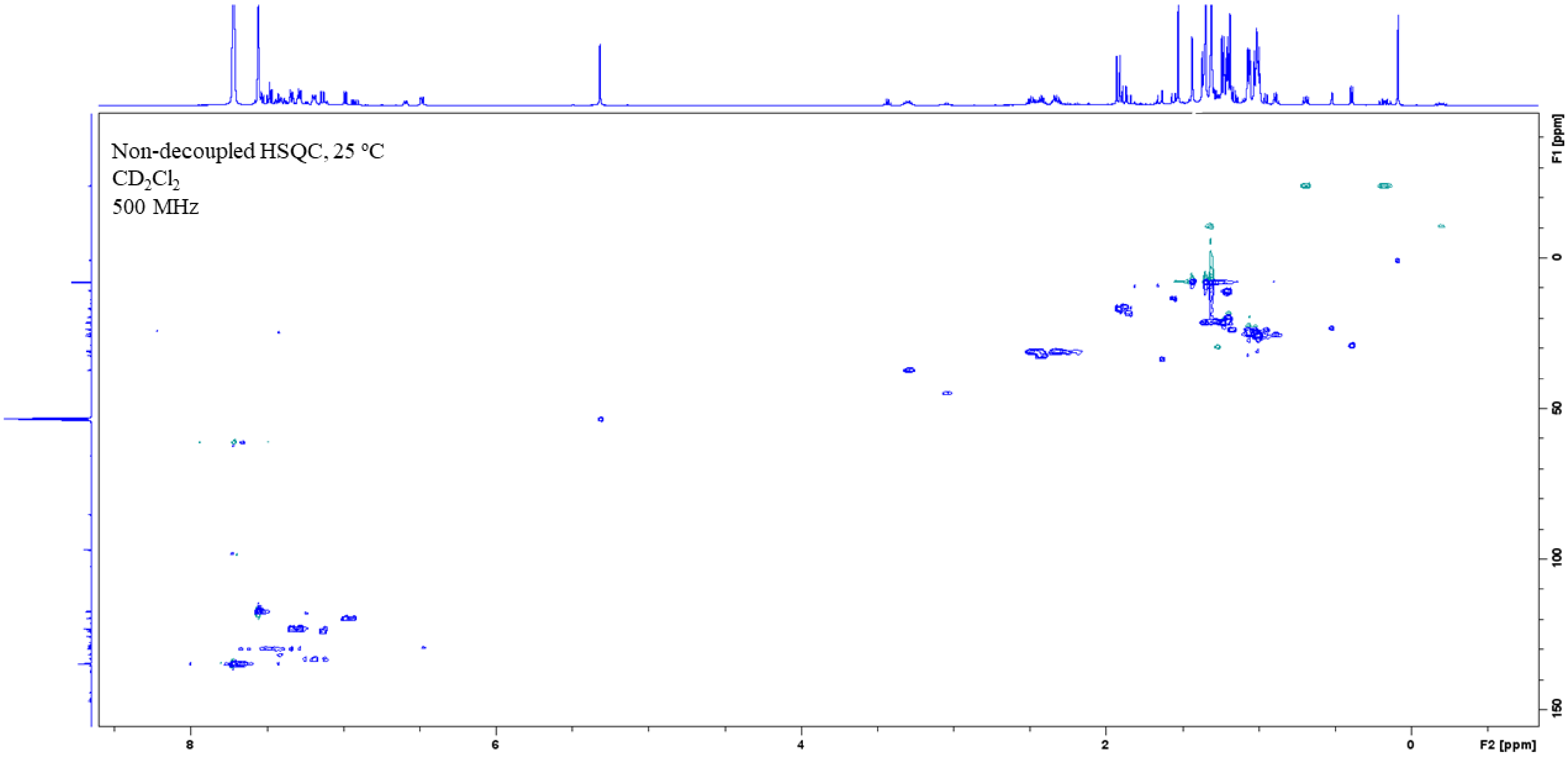

Figure S20. *Non-decoupled HSQC of a solution containing complexes* ***3***, ***5***, *and* ***5'***

4. X-Ray Structural Characterization of complexes

Crystallographic details. Crystals of compounds **2**, **4** and **5** were grown by slow diffusion of pentane into their CD_2Cl_2 solutions. Low-temperature diffraction data were collected on a Bruker APEX-II CCD diffractometer (**2·Me**) or a D8 Quest APEX-III single crystal diffractometer with a Photon III detector and a IμS 3.0 microfocus X-ray source (**2·Et**, **2·^{i}Pr**, **2·Et**, **4**, and **5)** at the Instituto de Investigaciones Químicas, Sevilla. Data were collected by means of ω and φ scans using monochromatic radiation λ(Mo Kα1) = 0.71073 Å. The diffraction images collected were processed and scaled using APEX-III software. Using Olex2[3–5], the structures were solved with SHELXT (**2·Bu** and **5**) or SHELXS (**2·Me**, **2·Et**, **2·^{i}Pr**, and **4**) and were refined against F^2 on all data by full-matrix least squares with SHELXL.[6] All non-hydrogen atoms were refined anisotropically. Hydrogen atoms were included in the model at geometrically calculated positions and refined using a riding model, except the hydride and the hydrogens of ethylene in compound **4** which have been determined from the Fourier map. The isotropic displacement parameters of all hydrogen atoms were fixed to 1.2 times the U value of the atoms to which they are linked (1.5 times for methyl groups).

A summary of the fundamental crystal and refinement data are given in **Table S1** and **Table S2**. Atomic coordinates, anisotropic displacement parameters and bond lengths and angles can be found in the cif files, which have been deposited in the Cambridge Crystallographic Data Centre with no. 2214486-2214490 and 2214512. These data can be obtained free of charge from The Cambridge Crystallographic Data Centre via www.ccdc.cam.ac.uk/data_request/cif.

Table S1. *Crystal data and structure refinement for compounds* ***2·Me***, ***2·Et***, *and* ***2·^{i}Pr***.

	**2·Me**	**2·Et**	**2·^{i}Pr**
formula	2($C_{43}H_{61}IrP$) + 2($C_{32}H_{12}BF_{24}$) + C_5H_{12}	$C_{44}H_{63}IrP$ + $C_{32}H_{12}BF_{24}$	$C_{45}H_{65}IrP$ + $C_{32}H_{12}BF_{24}$
Fw	3400.77	1678.34	1692.36
cryst.size, mm	0.29 × 0.15 × 0.10	0.16 × 0.14 × 0.10	0.18 × 0.15 × 0.12
crystal system	Monoclinic	Triclinic	Orthorhombic
space group	*C12/c1*	*P-1*	$P2_12_12_1$
a, Å	51.872(2)	12.6856(10)	17.7508(9)
b, Å	13.1846(6)	13.0984(9)	20.6070(13)
c, Å	25.9807(12)	22.5863(13)	21.6091(13)
α, deg	90	75.703(2)	90
β, deg	119.4780(10)	89.705(3)	90
γ, deg	90	89.201(3)	90
V, Å^3	15468.3(12)	3636.4(4)	7904.4(8)
T, K	100	100.0	100.0
Z	4	2	4
ρ_{calc}, g cm^{-3}	1.460	1.533	1.422
μ, mm^{-1} (MoKα)	1.846	1.962	1.806
F(000)	6856	1688	3408
absorption corrections	multi-scan, 0.53 – 0.75	multi-scan, 0.55 – 0.75	multi-scan, 0.59 – 0.75
θ range, deg	25.25 – 1.57	28.343 - 2.044	1.977 – 26.380
no. of rflns measd	131905	78500	61992
R_{int}	0.0314	0.0906	0.0551
no. of rflns unique	13995	18080	14218
no. of params / restraints	1007 / 132	957 / 0	993 / 0
R_1 ($I > 2\sigma(I)$) [a]	0.0321	0.0582	0.0340
R_1 (all data)	0.0379	0.0871	0.0460
wR_2 ($I > 2\sigma(I)$)	0.0873	0.1290	0.0778
wR_2 (all data)	0.0907	0.1153	0.0700
Diff.Fourier.peaks min/max, eÅ^{-3}	-0.817 / 1.337	- 3.435 / 2.725	- 0.946 / 1.739
CCDC number	2214486	2214490	2214489

Table S2. *Crystal data and structure refinement for compounds* ***2·^{n}Bu***, ***4*** *and* ***5***.

	**2·^{n}Bu**	**4**	**5**
5formula	$C_{46}H_{67}IrP + C_{32}H_{12}BF_{24}$	$C_{44}H_{63}IrP + C_{32}H_{12}BF_{24}$	$C_{42}H_{57}IrP + C_{32}H_{12}BF_{24}$
Fw	1706.39	1678.34	1648.27
cryst.size, mm	0.19 x 0.16 x 0.12	0.20 x 0.16 x 0.10	0.12 x 0.16 x 0.19
crystal system	Triclinic	Monoclinic	Triclinic
space group	*P-1*	*P 12^1/c1*	*P 12^1/c1*
a, Å	14.5756(12)	20.0588(14)	19.675(2)
b, Å	16.6568(13)	17.6632(12)	17.604(2)
c, Å	17.5949(13)	21.0499(15)	21.480(2)
α, deg	76.969(2)	90	90
β, deg	68.390(2)	105.593(3)	105.360(3)
γ, deg	76.611(3)	90	90
V, Å^3	3816.8(5)	7183.5(9)	7174.3(14)
T, K	193	100	193
Z	2	4	4
ρ_{calc}, g cm^{-3}	1.485	1.548	1.526
μ, mm^{-1} (MoKα)	1.871	1.986	1.987
F(000)	1720	3360	3304
absorption corrections	multi-scan, 0.58 – 0.75	multi-scan, 0.55 – 0.75	multi-scan, 0.59 – 0.75
θ range, deg	1.924 – 25.037	2.002 – 26.422	2.478 – 25.028
no. of rflns measd	113240	82739	212314
R_{int}	0.1231	0.1619	0.1096
no. of rflns unique	13467	14668	13625
no. of params / restraints	1051 / 9	974 / 1	929 / 0
R_1 ($I > 2\sigma(I)$) [a]	0.0416	0.0557	0.0607
R_1 (all data)	0.0682	0.1004	0.0857
wR_2 ($I > 2\sigma(I)$)	0.0750	0.0953	0.1272
wR_2 (all data)	0.0875	0.1099	0.1419
Diff.Fourier.peaks min/max, eÅ^{-3}	-0.963 / 1.144	- 1.051 / 0.814	- 0.936 / 1.051
CCDC number	2214512	2214488	2214487

5. Computational Details

Calculations were performed at the DFT level with the Gaussian 09 (Revision D.01) program.[7] The hybrid functional PBE0[8] was used throughout all computational studies. Dispersion effects were accounted for by using Grimme's D3 parameter set with Becke−Johnson (BJ) damping.[9] Geometry optimizations were carried out without geometry constraints, using the 6-31G(d,p)[10–12] basis set to represent the C, H, P, Cl, O, and Li atoms and the Stuttgart/Dresden Effective Core Potential and its associated basis set (SDD)[13] to describe the Ir atom. Bulk solvent effects (diethylether) were included at the optimization stage with the SMD continuum model.[14] The stationary points and their nature as minima or saddle points (TS) were characterized by vibrational analysis, which also produced enthalpy (H), entropy (S) and Gibbs energy (G) data at 298.15 K. The minima connected by a given transition state were determined by perturbing the transition states along the TS coordinate and optimizing to the nearest minimum. Free energies were corrected (ΔG^{qh}) to account for errors associated with the harmonic oscillator approximation. Thus, according to Truhlars's quasi-harmonic approximation for vibrational entropy, all vibrational frequencies below 100 cm^{-1} were set to this value.[15] These anharmonic and concentration corrections were calculated with the Goodvibes code.[16] QTAIM analyses were performed with the Multiwfn[17] software on wavefunctions generated with the Gaussian 09 program.

For the computational calculation of reaction pathways, the approach followed was that of previous publications choosing prototypical monomers of alkylithium[18] and alkylmagnesium[19–23] species and carefully evaluating their solvation.

5.1. β-elimination.

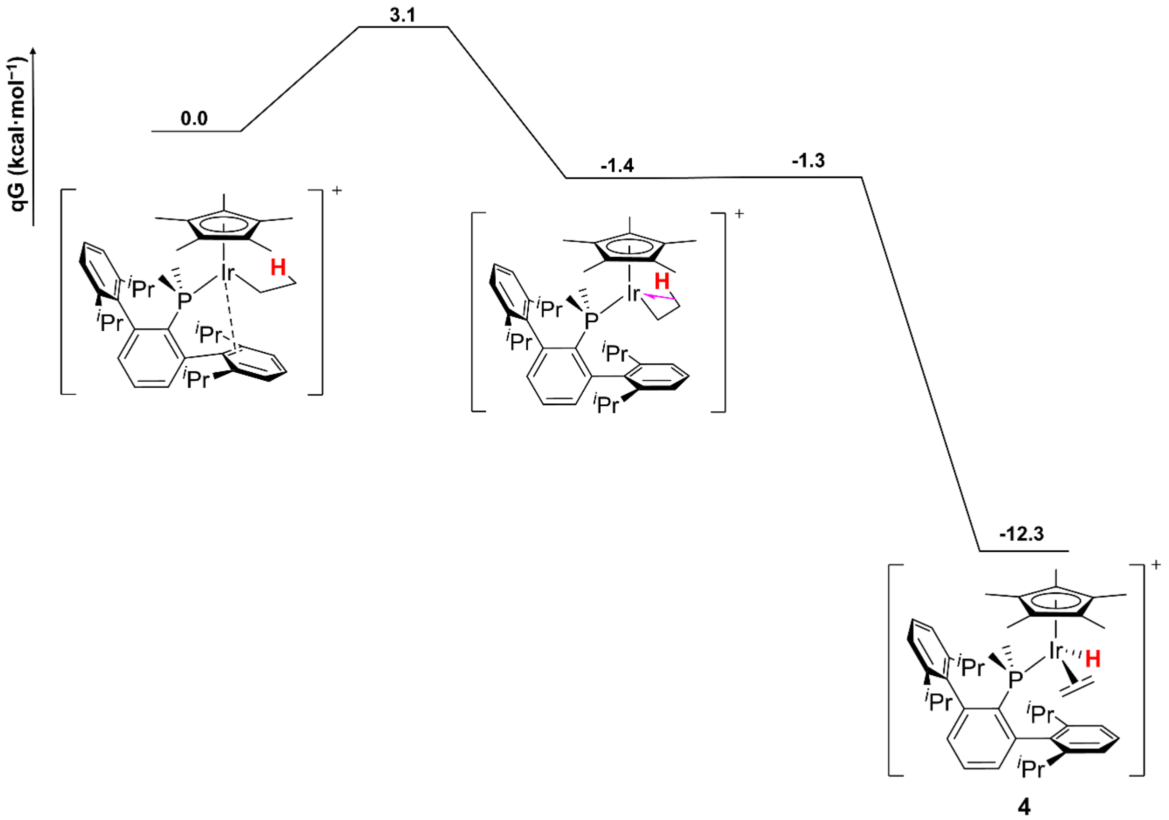

***Figure S21**. Free energy profile of the proposed β-hydride elimination leading to complex **4**.*

The proposed mechanism involves the formation of the Ir−Et complex (0.0 kcal/mol), which undergoes a rearrangement leading to the formation of an agostic interaction (−1.4 kcal/mol). Then, overcoming a barrier of 0.1 kcal/mol leads to complex **4** (−12.3 kcal/mol).

5.2. Comparison: LiPh as a base *versus* as a nucleophile

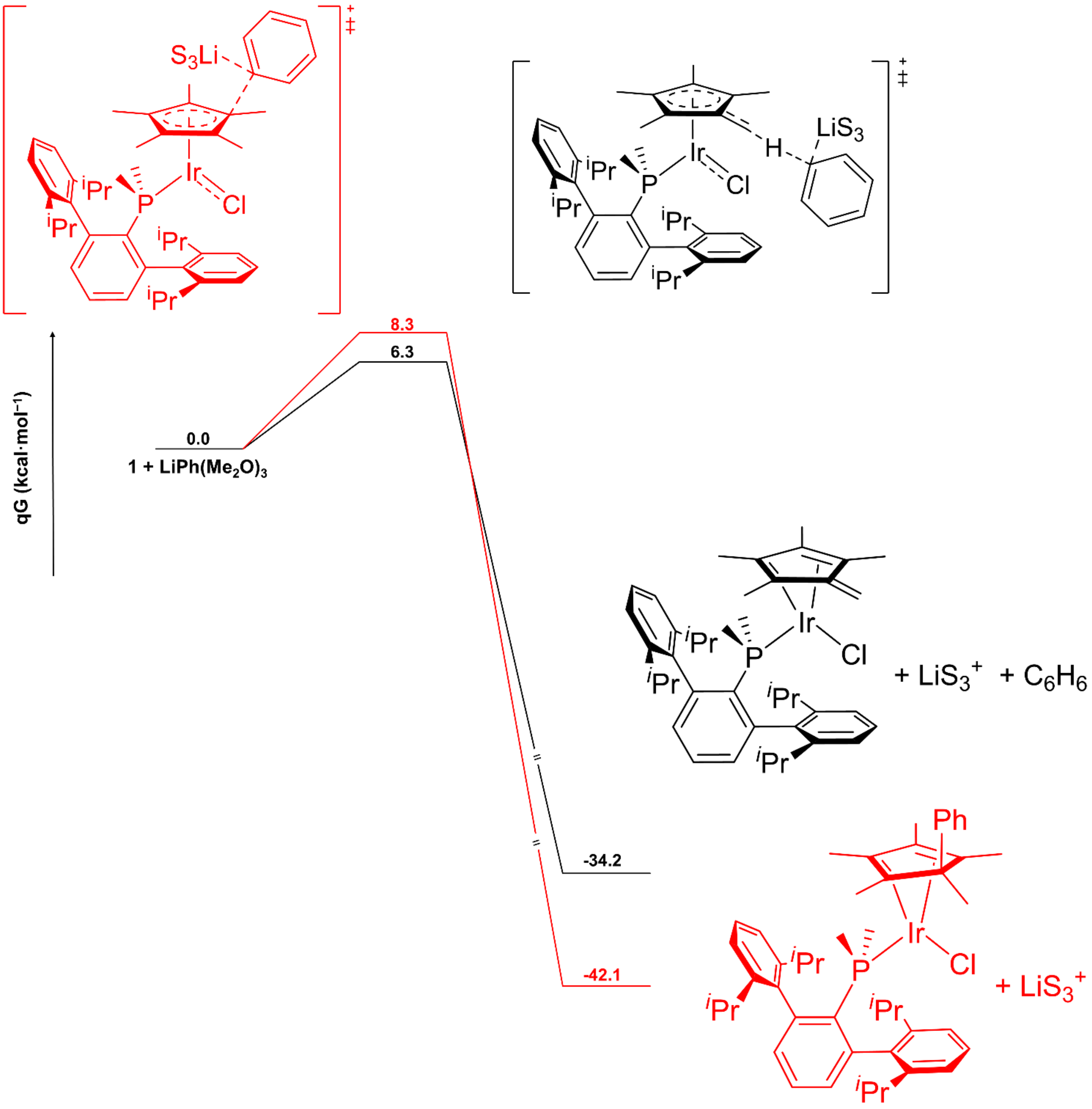

Figure S22. *Free energy profiles of LiPh acting as a base (black) or as a nucleophile (red). Explicit solvent molecules (Me_2O) responsible for stabilizing the Li atom included in the calculations are represented as S.*

5.3. Comparison. LiMe attacking the Ir v. attacking one of the internal C of Cp* v. acting as base

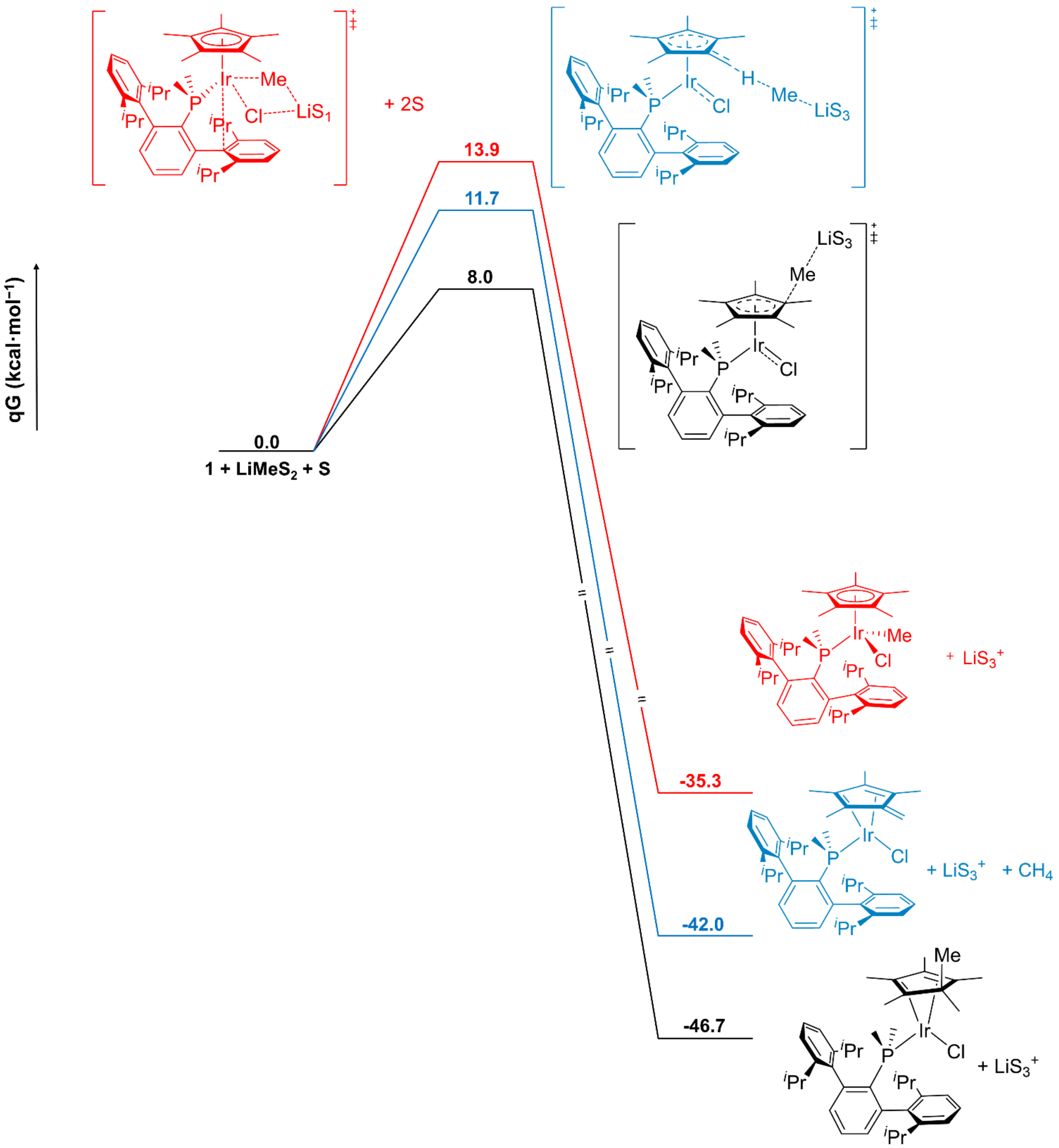

Figure S23. *Free energy profile comparison of LiMe attacking the Cp* (black), the metal centre (red), or acting as a base (blue). Explicit solvent molecules (Me$_2$O) responsible for stabilizing the Li atom included in the calculations are represented as S.*

5.4. Energy profile for the chloride release to give complex 2·Me.

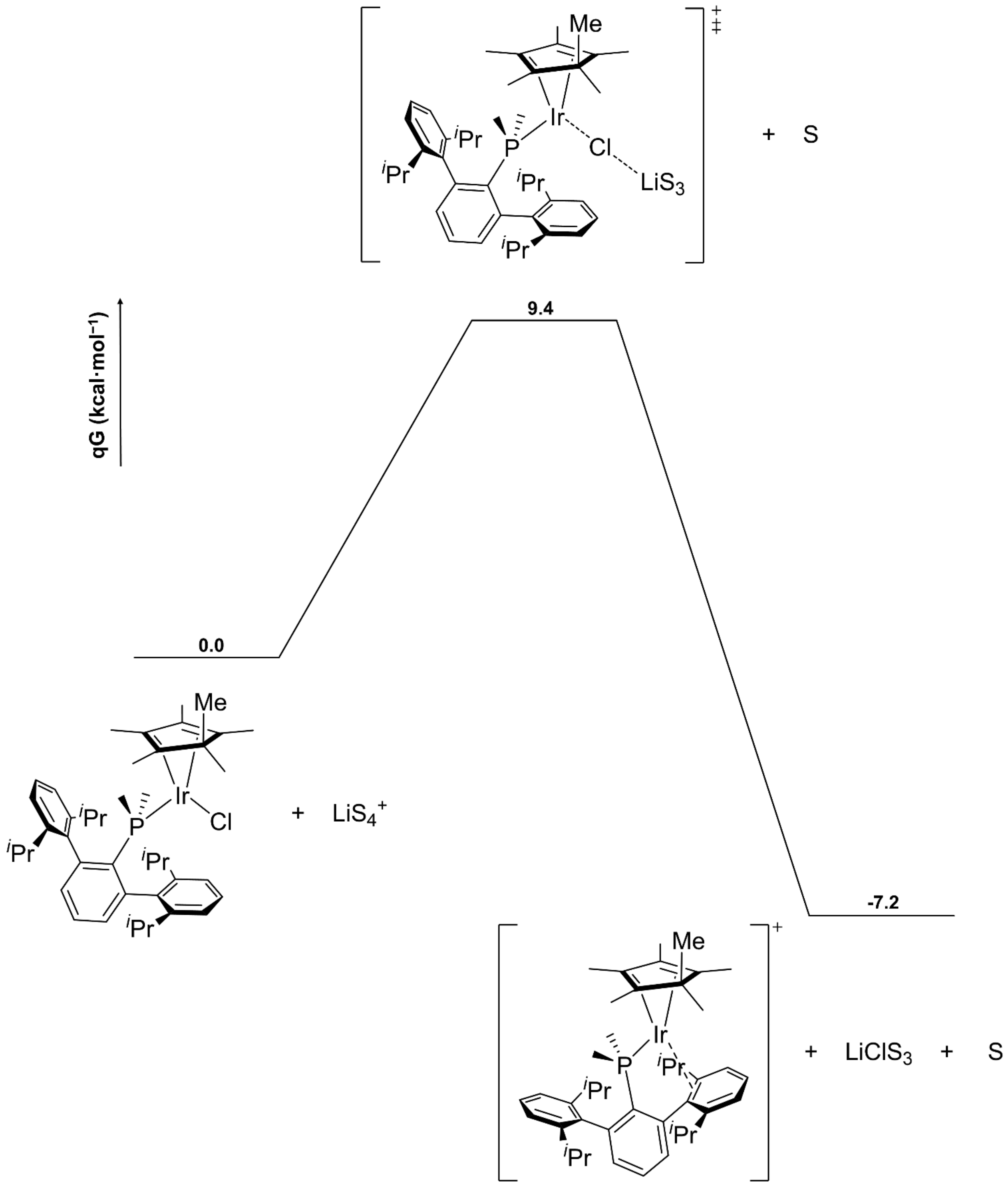

Figure S24. *Free energy profile for the assisted release of chloride. Explicit solvent molecules (Me_2O) responsible for stabilizing the Li atom included in the calculations are represented as S*

5.5. Energy profile for reductive coupling (route to obtain complex 2·Me)

The formation of complex **A** followed by a reductive coupling between Ir and the Cp* ligand was studied as a possibility to obtain complex **2**, but this mechanism was found to be inaccessible.

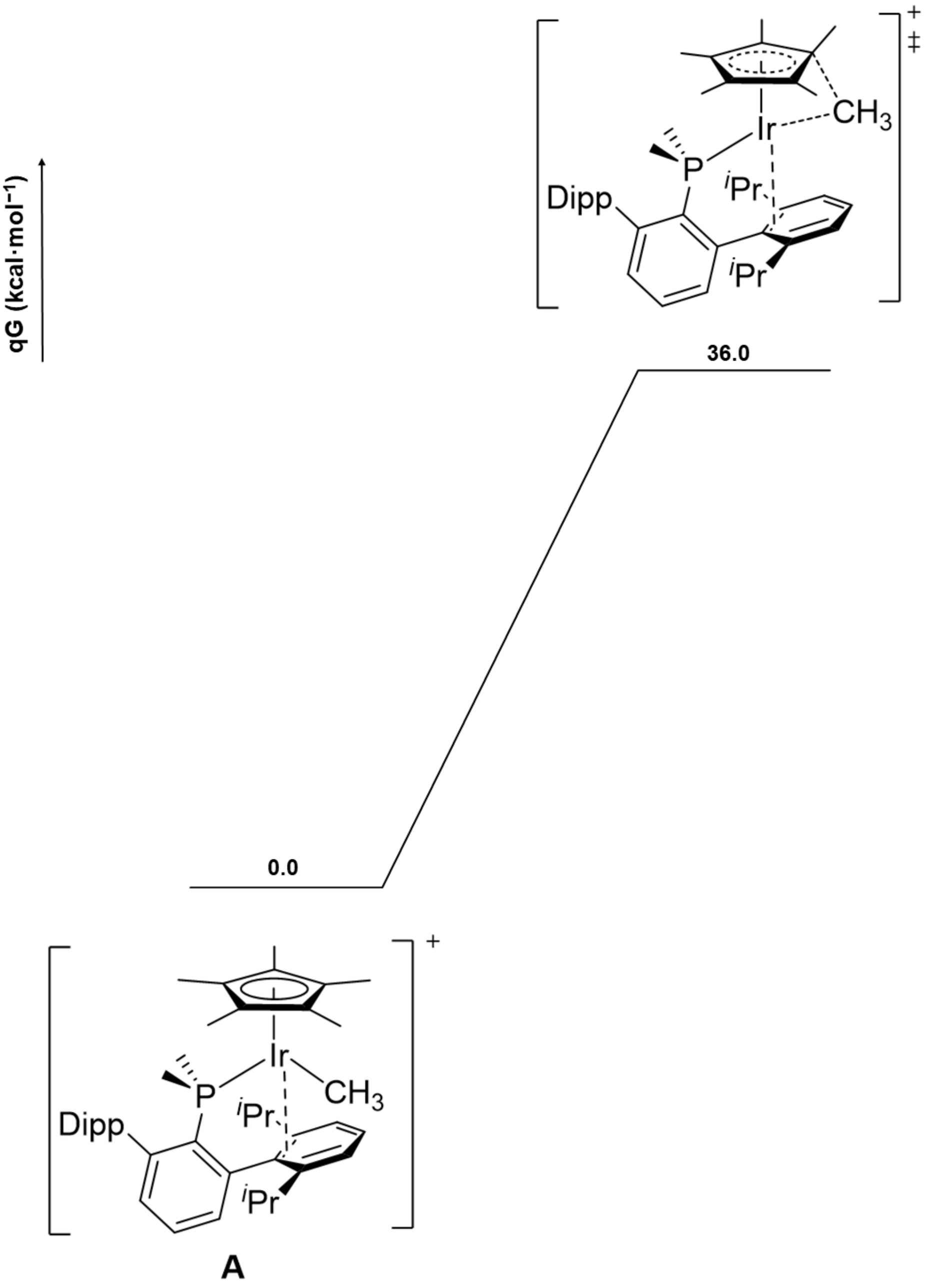

Figure S25. *Free energy profile for the reductive coupling en route to complex **2**.*

5.6. Reductive coupling in an associative process to obtain complex 2·Me

The addition of the methyl group to the Ir center, followed by the reductive coupling of the methyl and the Cp* ligands was studied as a possibility to obtain complex **2·Me**, but this mechanism was found to be inaccessible even assuming the formation of the neutral saturated complex is thermoneutral.

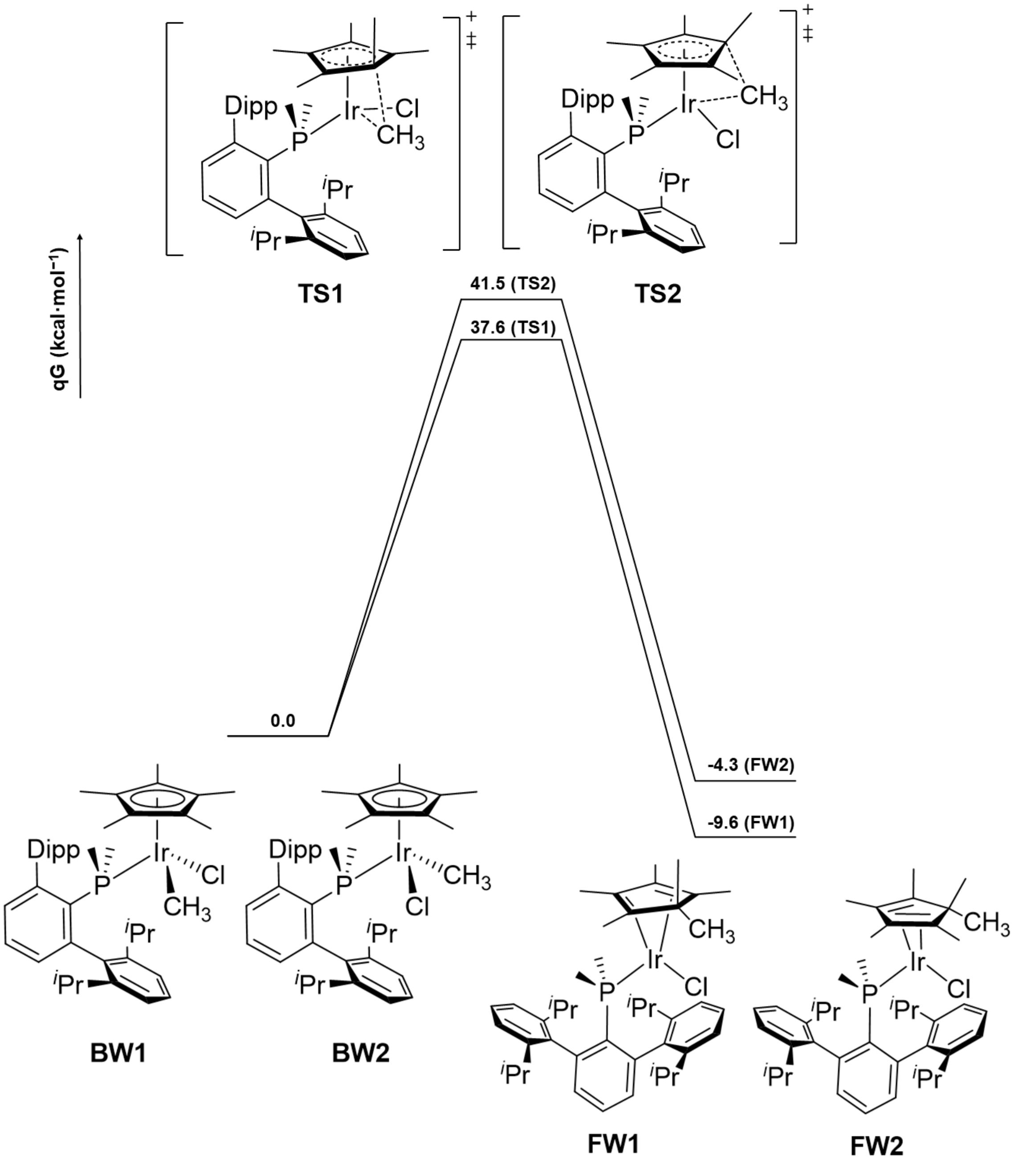

***Figure S26**. Free energy profile for the reductive coupling in route to complex **2·Me**, the two routes correspond to the two possible stereochemical configurations of iridium*

5.7. Comparison. MeMgCl attacking the Ir v. attacking one of the internal C of Cp*

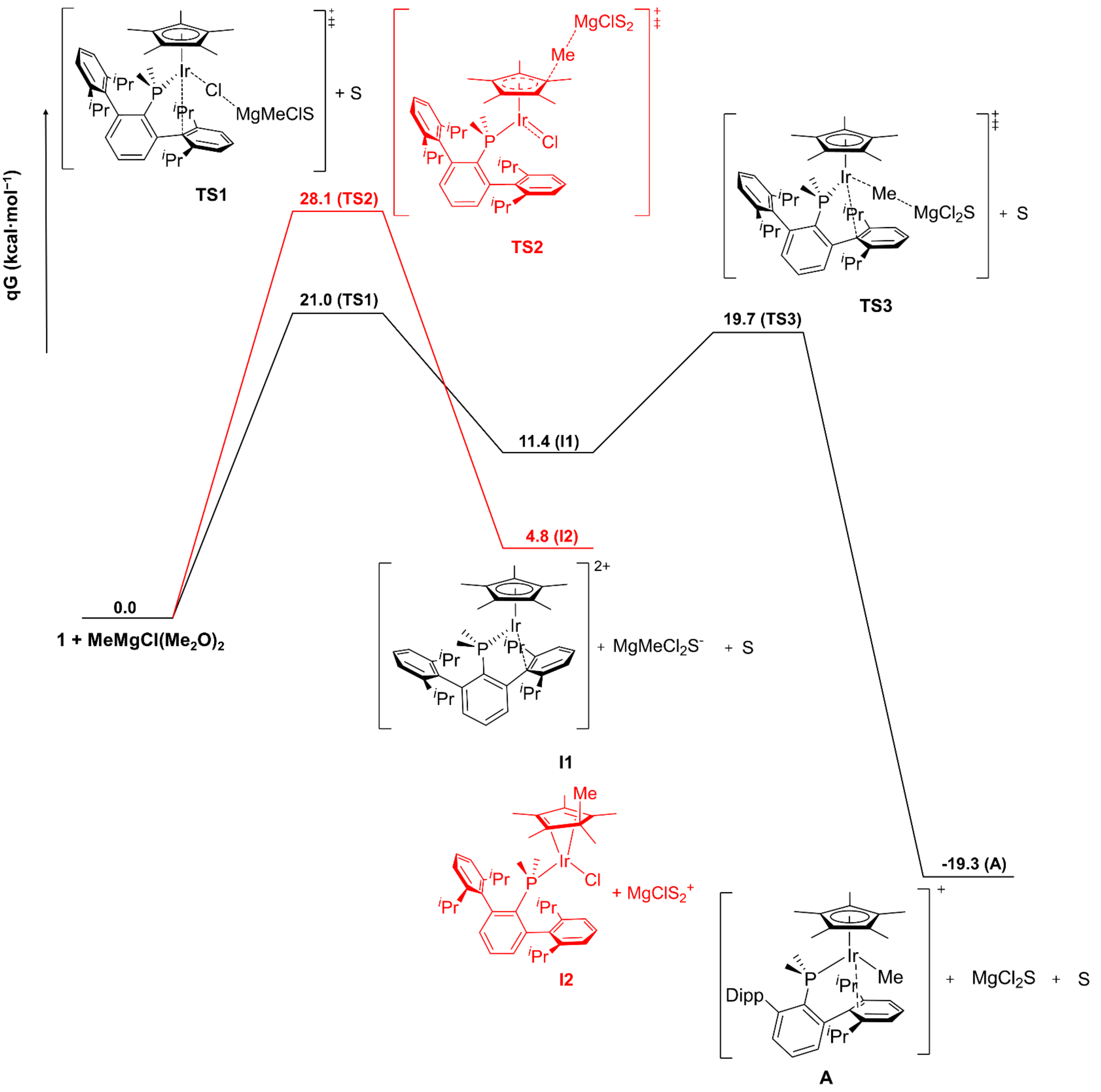

Figure S27. Free energy profile comparison of MeMgCl attacking the Cp (red) or the metal centre (black). The black pathway leads to proposed intermediate **A**. Explicit solvent molecules (Me_2O) responsible for stabilizing the Mg atom included in the calculations are represented as S.*

5.8. Alternative route to MeMgCl attacking the metal

The addition of the methyl group to the Ir center, followed by the release of the chloride ligand was also studied as a possibility to obtain **A**, but this route was found to be inaccessible.

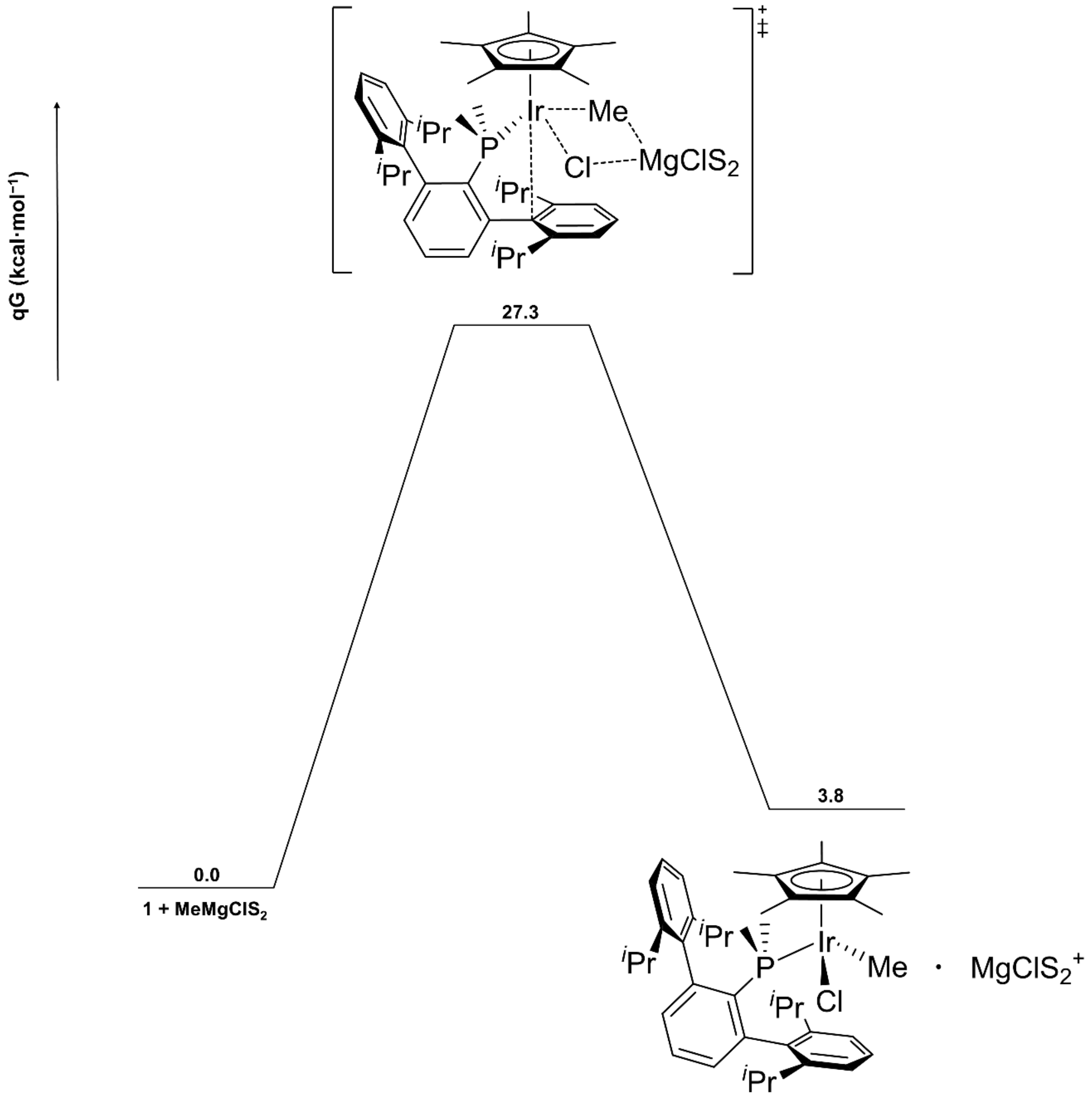

Figure S28. *Free energy profile of an inaccessible alternative route to obtain **A**.*

5.9. Alternatives to the pathway depicted in Fig. 6

Efforts to explore either the hydride formation via the abstraction of the agostic proton of the complex **I3** in Fig. 6 or to activate the other methyl group via sigma bond metathesis resulted in no saddle points.

5.10. Energy profile accounting for the solvation of LiMe

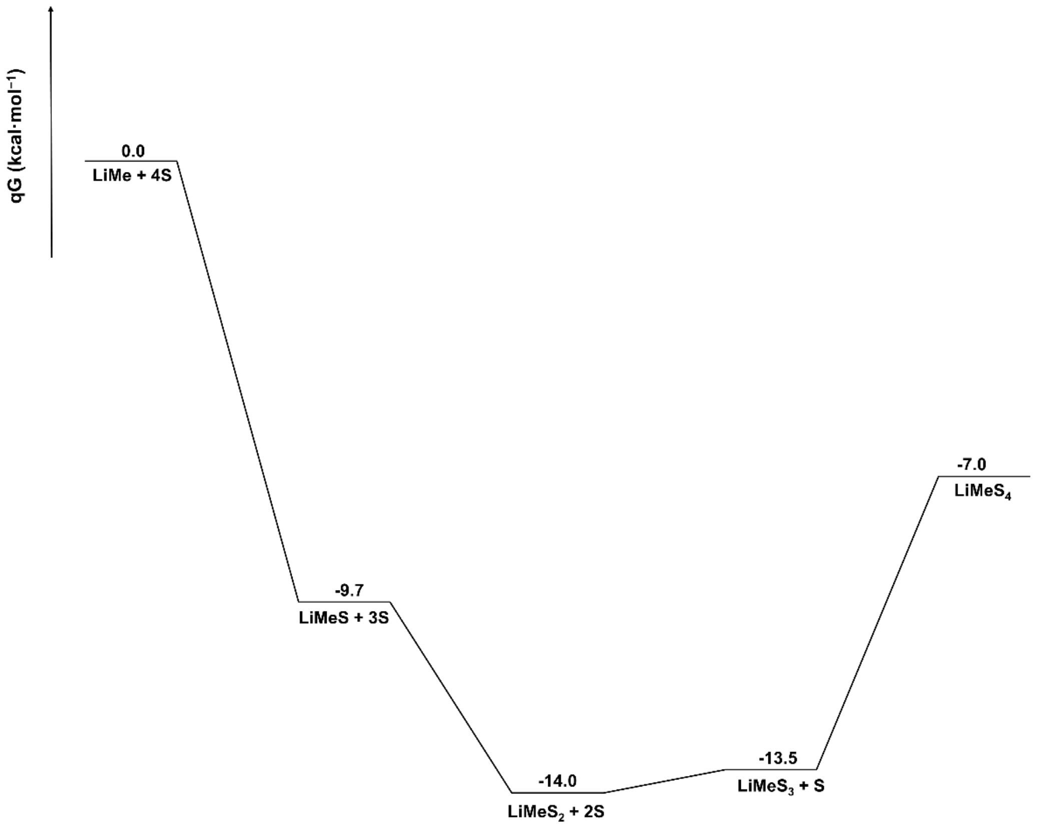

Figure S29. *Free energy profile showing that the most stable form of LiMe presents two explicit solvent molecules (S, Me_2O) coordinated to the Li atom.*

5.11. Energy profile accounting for the solvation of MeMgCl

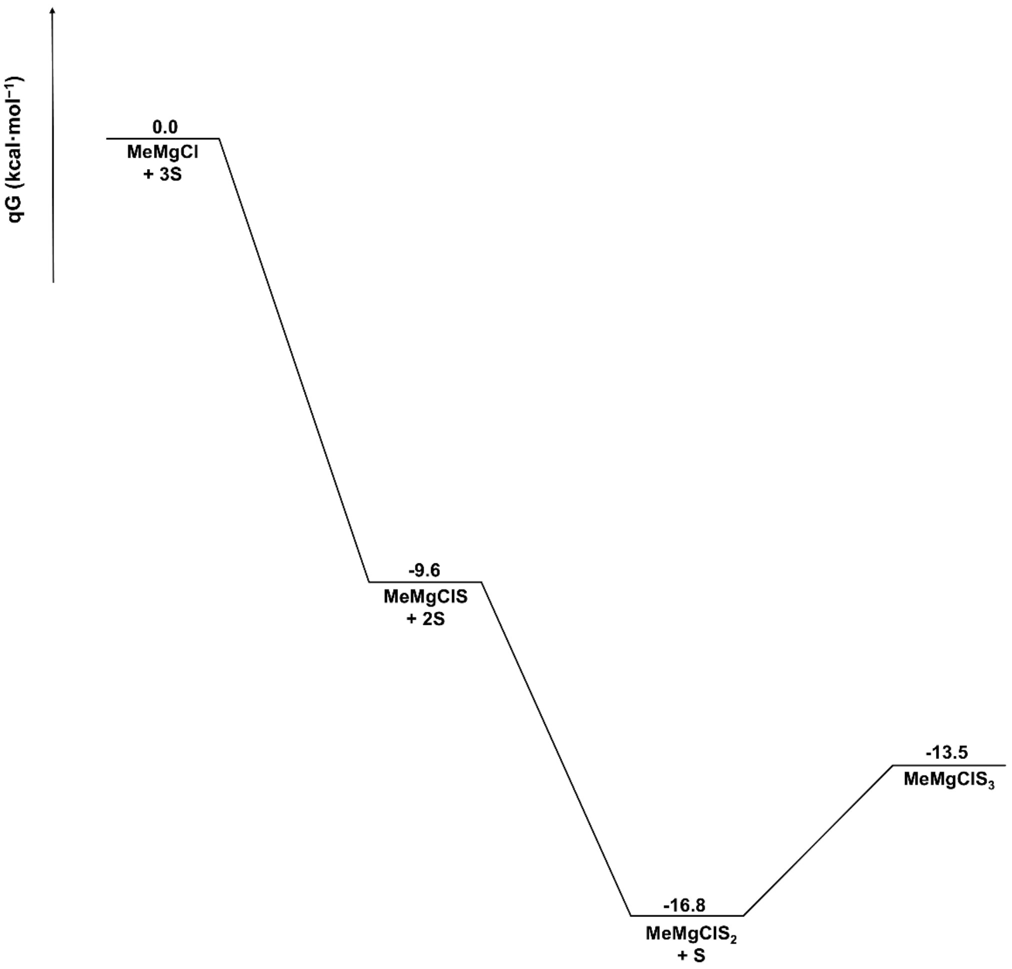

Figure S30. *Free energy profile showing that the most stable form of MeMgCl presents two explicit solvent molecules (S, Me_2O) coordinated to the Mg atom.*

5.12. Energy profile accounting for the solvation of Li^+

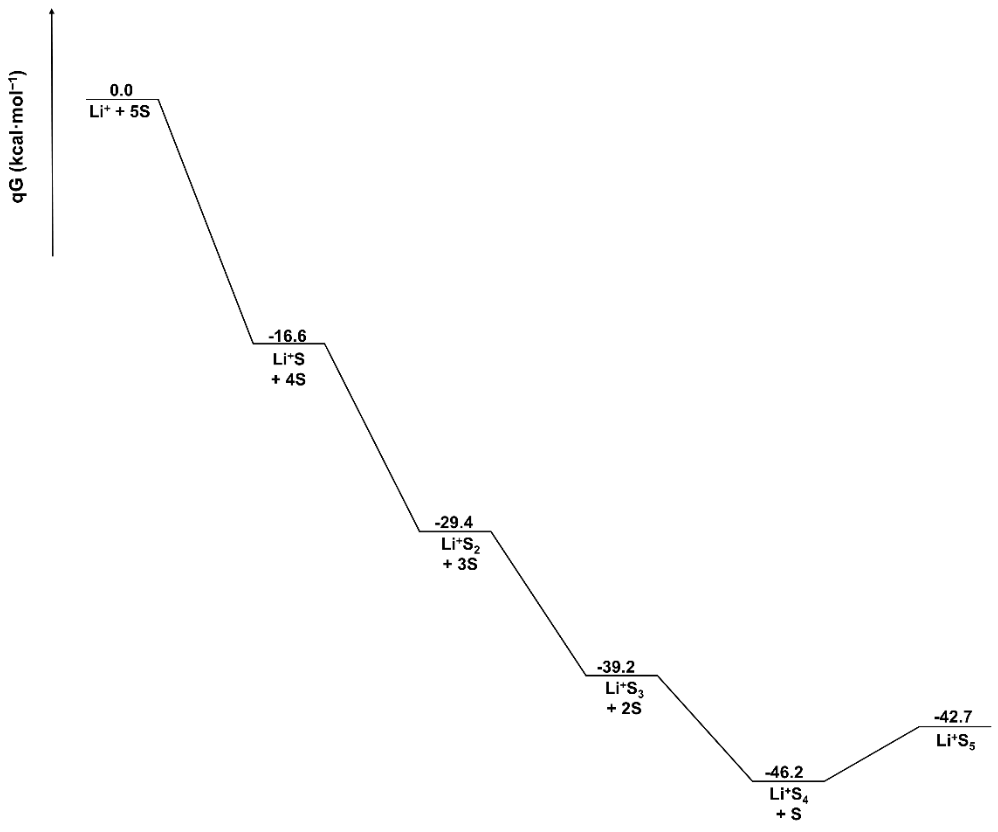

Figure S31. *Free energy profile showing that the most stable form of LPh presents four explicit solvent molecules (S, Me_2O) coordinated to the Li cation.*

5.13. Energy profile accounting for the solvation of LiPh

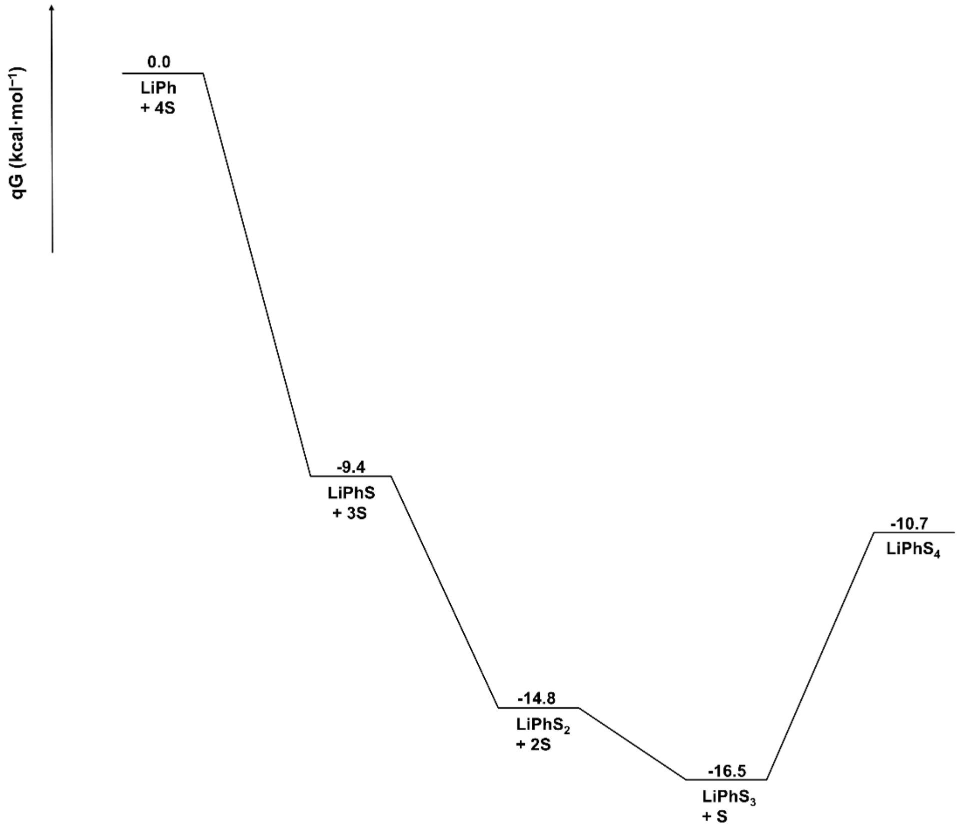

Figure S32. *Free energy profile showing that the most stable form of LiPh presents three explicit solvent molecules (S, Me_2O) coordinated to the Li atom.*

5.14. Energy profile accounting for the solvation of Li^{i}Pr

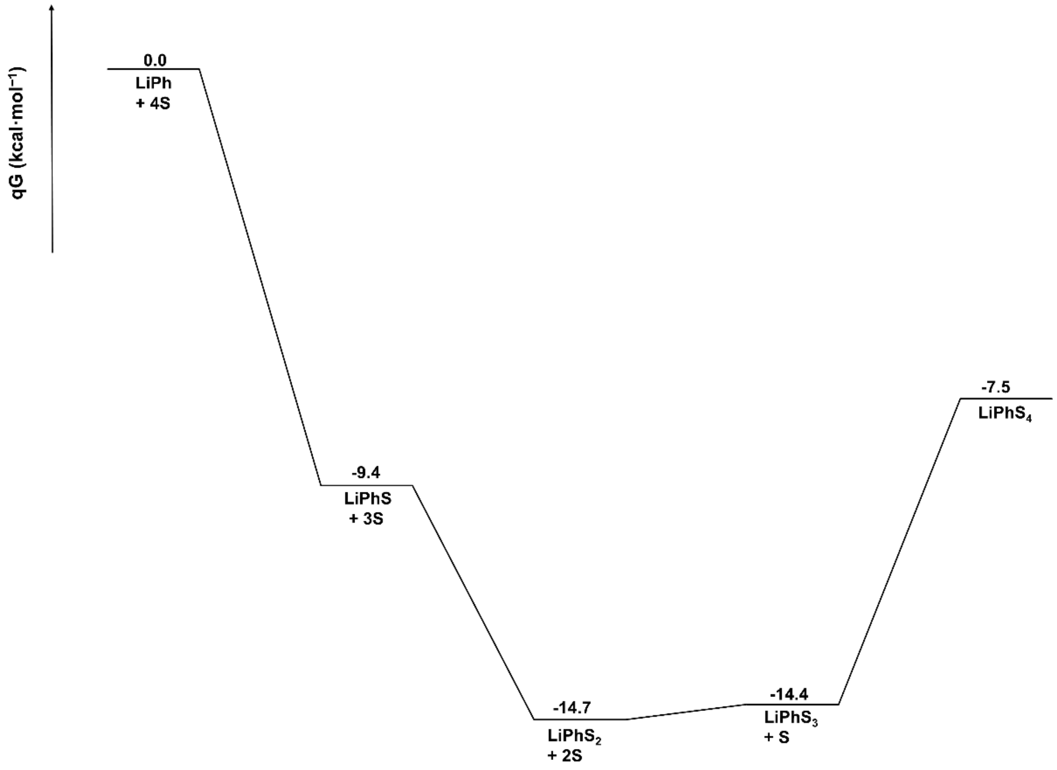

Figure S33. *Free energy profile showing that the most stable form of LiPh presents two explicit solvent molecules (S, Me_2O) coordinated to the Li atom*

5.15. Comparison. Li^{i}Pr attacking one of the internal C of Cp* v. acting as a base

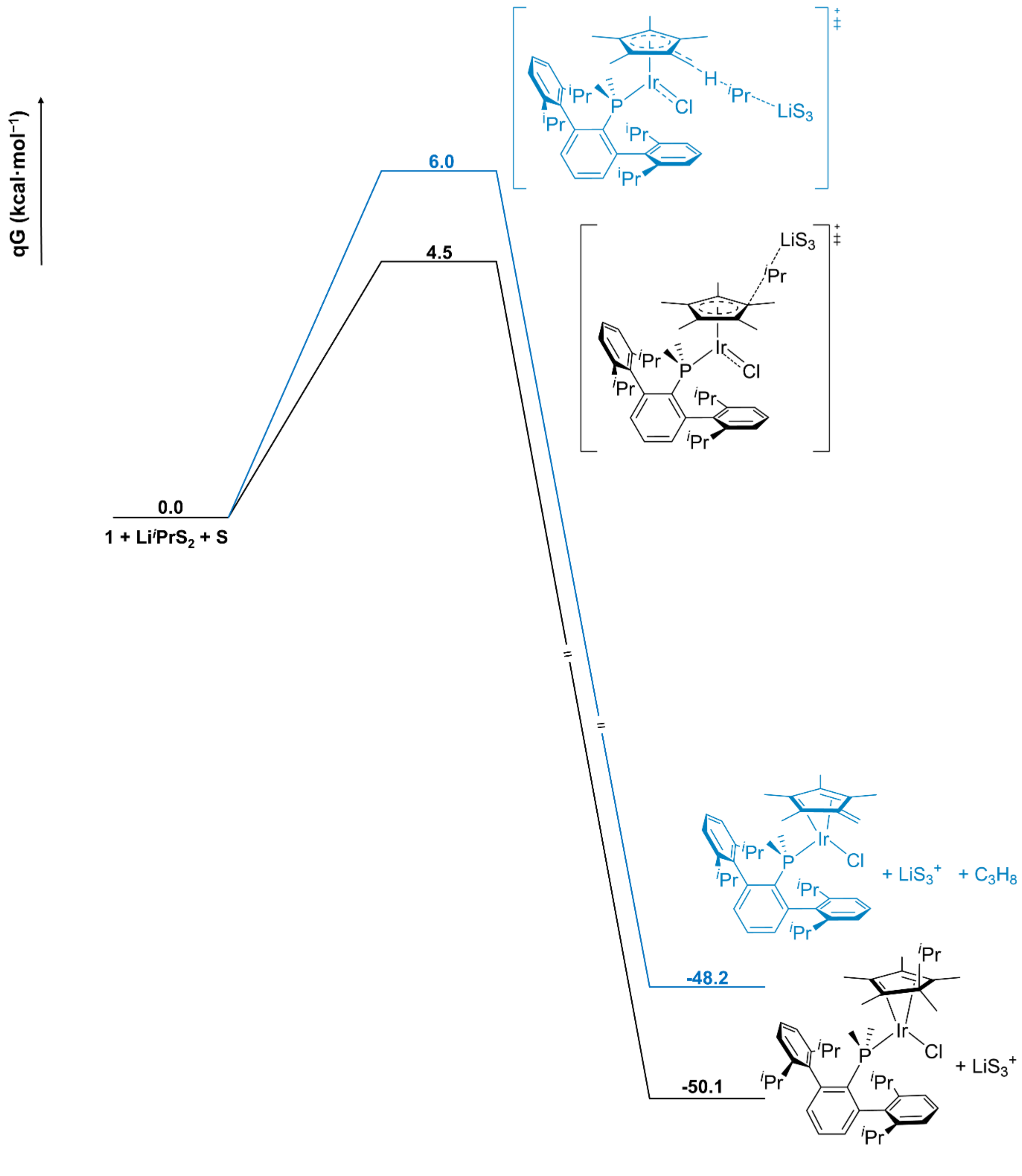

Figure S34. *Free energy profile comparison of Li^{i}Pr attacking the Cp* (black) or acting as a base (blue). Explicit solvent molecules (Me$_2$O) responsible for stabilizing the Li atom included in the calculations are represented as S.*

5.16. Topological Studies

The value of the electron density at a bond critical point (ρ) may be linked to the strength of such bond. The value of the laplacian of the electron density at a bcp, $\nabla^2\rho$, is negative in covalent interactions (indicative of accumulation of electron density charge) and positive in closed shell or ionic interactions (indicative of depletion of electron density charge). Moreover, the total energy density (H) tends to be positive for ionic interactions and negative for covalent interactions. The nature of an interaction can also be determined by the ratio between the local Potential (V) and Kinetic (G) energies. Covalent interactions feature a $|V|/G > 2$, while a ratio $|V|/G < 1$ tends to be found in ionic interactions. Intermediate cases (metal-metal and metal-ligand interactions) are instead associated to positive values of $\nabla^2\rho$, close to zero H, and $1 < |V|/G < 2$).[24–26]

5.16.1. Topological analysis of the TS3.2 of Fig. 6

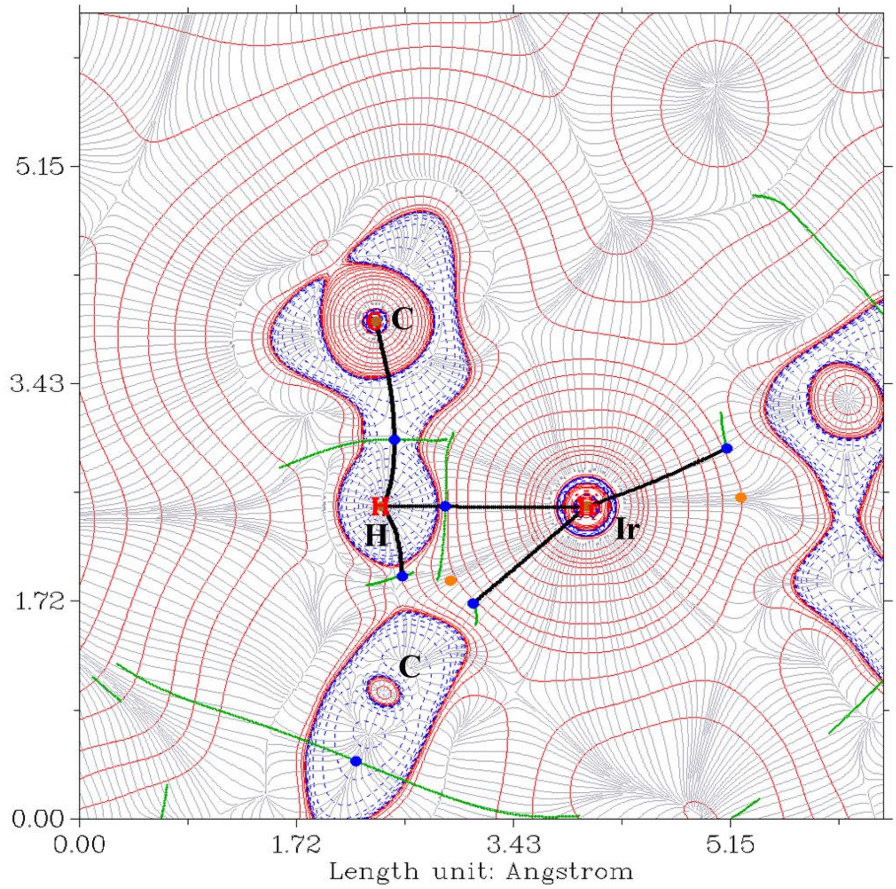

Figure S35. *Plot of the laplacian of the electron density,* $\nabla^2\rho$*, of the TS3.2 (Figure 6) in the C(methyl), H(agostic), Ir plane. The solid and dashed lines correspond to positive and negative values of* $\nabla^2\rho$ *respectively. Interatomic basins are represented in green. In plane bcps and bond paths of the electron density are superimposed. The C atom of the nascent* CH_2 *fragment is partially out of the plane, but the bcp and part of the bond paths connecting this atom with the H and Ir centres are shown.*

Table S3. *Selected properties of the electron density at relevant bcps shown in* ***Figure S35***

bond	ρ^b	G_a^c	V_a^c	H_a^c	$\lvert V_a \rvert /G_a$	$\nabla^2\rho^e$
**Me-H**	1.19E−01	4.28E−02	−9.96E−02	-5.68E-02	2.32E+00	−5.57E-02
**Ir-H**	1.32E−01	1.05E−01	−1.76E−01	-7.11E-02	1.68E+00	1.44E−01
**CH_2-H**	9.92E−02	3.98E−02	−7.78E−02	-3.81E-02	1.96E+00	6.85E−03
**CH_2-Ir**	9.39E−02	6.31E−02	−9.40E−02	-3.09E-02	1.49E+00	1.33E−01

[a] *average values,* [b] *e·bohr*$^{-3}$*,* [c] *Hartree,* [d] *e·bohr*$^{-5}$*, e = elementary charge. Main takeaway: the bcp between the H and the* CH_3 *has a covalent character at the TS.*

5.16.2. Topological analysis of complex 2·Me

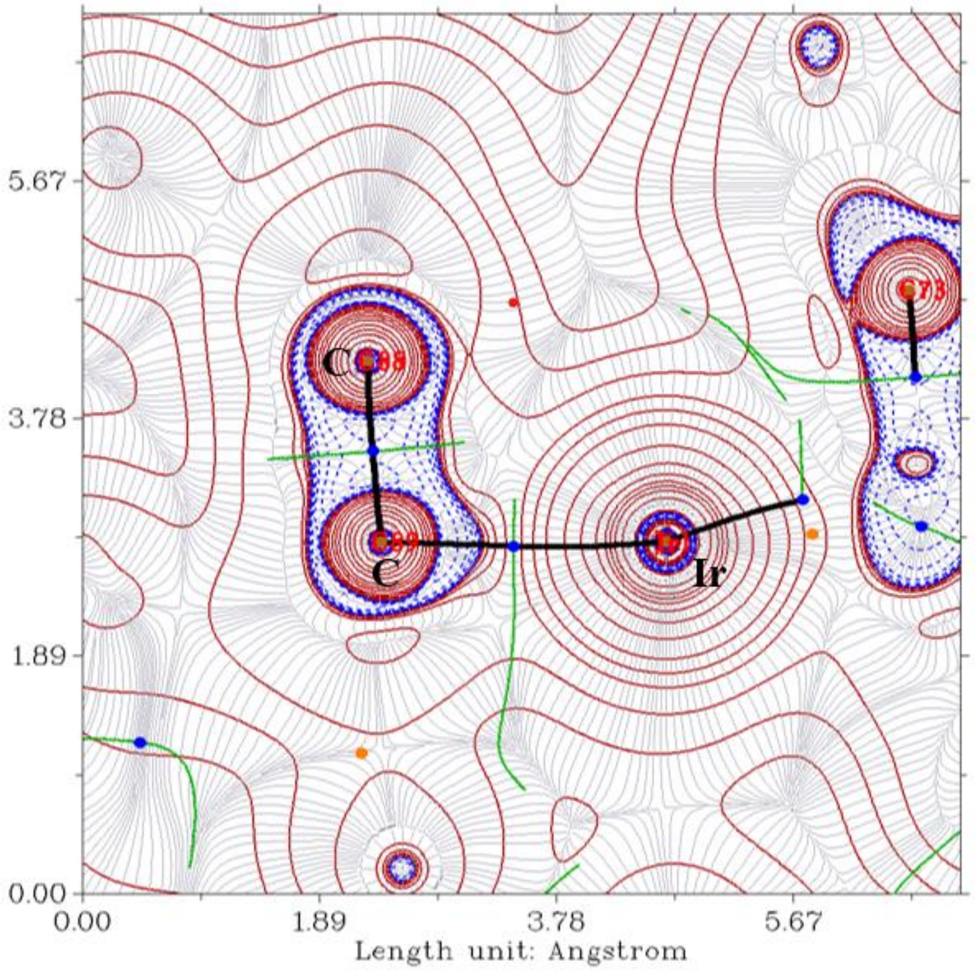

Figure S36. *Plot of the laplacian of the electron density,* $\nabla^2\rho$*, of complex* ***2·Me*** *in the plane containing the Ir,* C_{ipso}*, and one of the* C_{ortho} *atoms. Both C atoms belong to the Dipp which features an interaction with the metal centre. The solid and dashed lines correspond to positive and negative values of* $\nabla^2\rho$ *respectively. Interatomic basins are represented in green. In plane bcps and bond paths of the electron density are superimposed.*

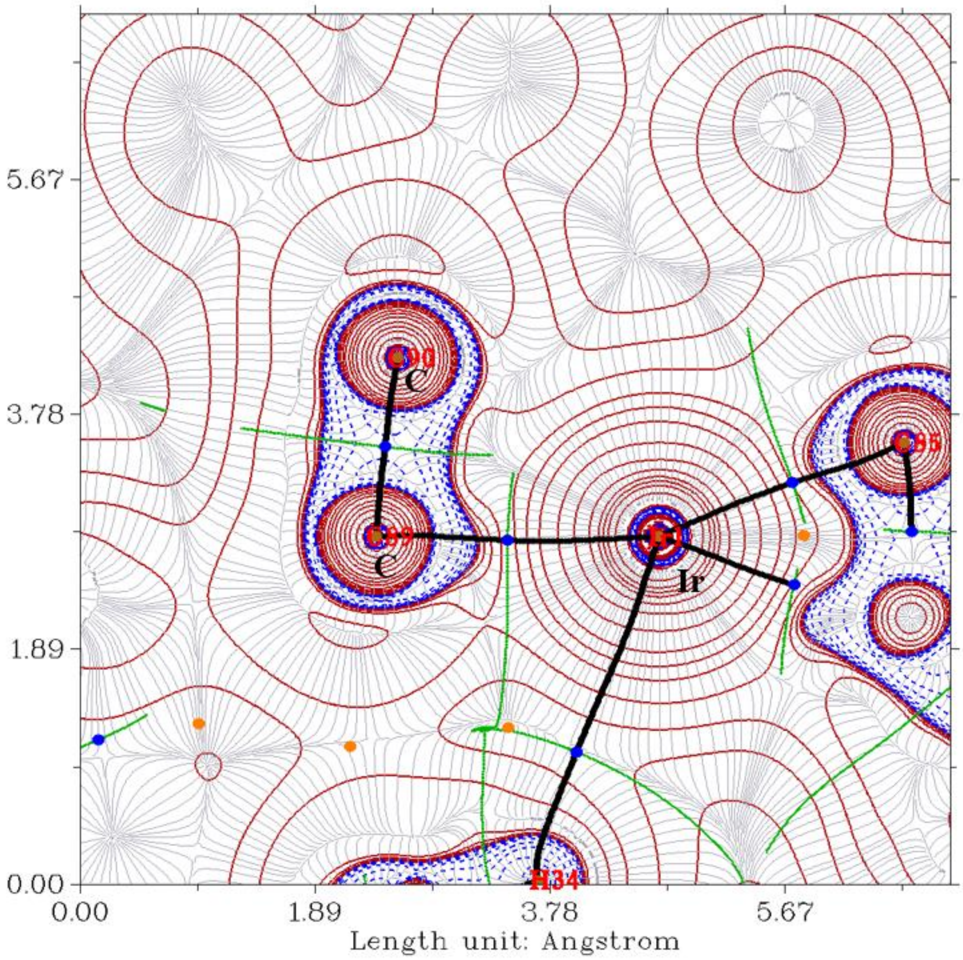

***Figure S37**. Plot of the laplacian of the electron density, $\nabla^2\rho$, of complex **2·Me** in the Ir, C_{ipso}, and one of the C_{ortho} (different from **Figure S36**). Both C atoms belong to the Dipp which features an interaction with the metal centre. The solid and dashed lines correspond to positive and negative values of $\nabla^2\rho$ respectively. Interatomic basins are represented in green. In plane bcps and bond paths of the electron density are superimposed.*

bond	ρ^b	$G_a{}^c$	$V_a{}^c$	$H_a{}^c$	$\lvert V_a \rvert / G_a$	$\nabla^2\rho^e$
**Ir-C$_{ipso}$**	7.93E−02	5.75E−02	−8.44E−02	−2.69E−02	1.47E+00	1.25E−01

^{a} *average values*, ^{b} *e·bohr^{-3}*, ^{c} *Hartree*, ^{d} *e·bohr^{-5}*, *e = elementary charge.*

5.17. EDA-NOCV

Energy values (in kcal/mol) were computed at the ZORA-BP86-D3/TZ2P//PCM(DCM)-BP86-D3/6-31G(d)&SDD(f) level.

5.17.1. EDA-NOCV of complex 2·Me: C_5Me_6Ir / $PMe_2Ar^{Dipp}{}_2$

$\Delta E_{int} = -151.94$ kcal/mol

$\Delta E_{Pauli} = 316.33$ kcal/mol

$\Delta E_{elstat} = -258.66$ kcal/mol

$\Delta E_{orb} = -172.40$kcal/mol

$\Delta E_{disp} = -36.83$ kcal/mol

The main orbitalic contribution are the following:

1. −62.61963 kcal/mol

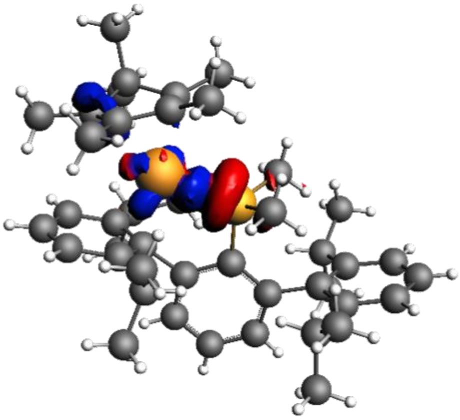

Figure S38. Sigma donation from the P atom to the Ir centre.

2. −33.51837 kcal/mol

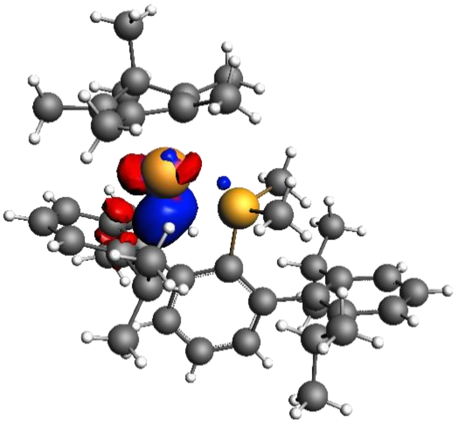

Figure S39*. Donation from the terphenyl phosphine to the [Ir-C_5Me_6]$^+$ fragment and backdonation of the latter.*

5.17.2. EDA-NOCV of complex 2·Me: C_5Me_6 / $IrPMe_2Ar^{Dipp}_2$

$$\Delta E_{int} = -118.57 \text{ kcal/mol}$$

$$\Delta E_{Pauli} = 290.64 \text{ kcal/mol}$$

$$\Delta E_{elstat} = -207.79 \text{ kcal/mol}$$

$$\Delta E_{orb} = -172.40 \text{kcal/mol}$$

$$\Delta E_{disp} = -29.03 \text{ kcal/mol}$$

Carbon atoms 19 and 20 (*trans* to P) are involved in the longer formal double bond while carbon atoms 22 and 27 (*trans* to the arene) are involved in the shorter formal double bond.

1. −56.11763 kcal/mol

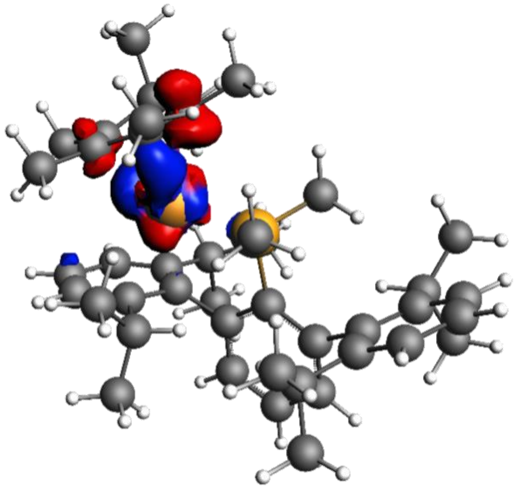

Figure S40. *Donation of electron density from the C_5Me_6 moiety to the Ir centre.*

Carbon atoms 19 and 20 contribute 44.34%

Carbon atoms 22 and 27 contribute 38.23%

2. −54.8719 kcal/mol

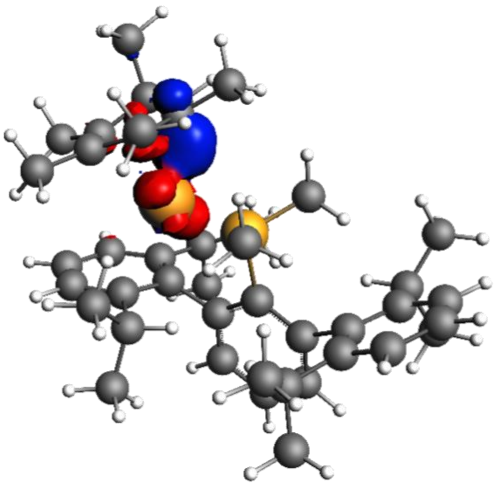

***Figure S41**. Backdonation from the Ir centre to the C_5Me_6 moiety.*

Carbon atoms 19 and 20 contribute 44.57%

Carbon atoms 22 and 27 contribute 33.83%

6. References

(1) Moreno, J. J.; Espada, M. F.; Campos, J.; López-Serrano, J.; Macgregor, S. A.; Carmona, E. Base-Promoted, Remote C-H Activation at a Cationic (η^5-C_5Me_5)Ir(III) Center Involving Reversible C-C Bond Formation of Bound C_5Me_5. *J. Am. Chem. Soc.* **2019**, 141, 2205-2210. https://doi.org/10.1021/jacs.8b11752.

(2) Ortega-Moreno, L.; Peloso, R.; Maya, C.; Suárez, A.; Carmona, E. Platinum(0) Olefin Complexes of a Bulky Terphenylphosphine Ligand. Synthetic, Structural and Reactivity Studies. *Chem. Commun.* **2015**, *51* (95), 17008–17011. https://doi.org/10.1039/c5cc07308a.

(3) Sheldrick, G. M. Crystal Structure Refinement with SHELXL. *Acta Crystallogr. Section C* **2015**, *71* (1), 3–8. https://doi.org/10.1107/S2053229614024218.

(4) Dolomanov, O. v; Bourhis, L. J.; Gildea, R. J.; Howard, J. A. K.; Puschmann, H. OLEX2: A Complete Structure Solution, Refinement and Analysis Program. *J. Appl. Crystallogr.* **2009**, *42* (2), 339–341. https://doi.org/10.1107/S0021889808042726.

(5) Bourhis, L. J.; Dolomanov, O. v; Gildea, R. J.; Howard, J. A. K.; Puschmann, H. The Anatomy of a Comprehensive Constrained, Restrained Refinement Program for the Modern Computing Environment – Olex2 Dissected. *Acta Crystallogr. Section A* **2015**, *71* (1), 59–75. https://doi.org/10.1107/S2053273314022207.

(6) Sheldrick, G. M. A Short History of SHELX. *Acta Crystallogr. Section A* **2008**, *64* (1), 112–122. https://doi.org/10.1107/S0108767307043930.

(7) M. J. Frisch, G. W. Trucks, H. B. Schlegel, G. E. Scuseria, M. A. Robb, J. R. Cheeseman, G. Scalmani, V. Barone, B. Mennucci, G. A. Petersson, H. Nakatsuji, M. Caricato, X. Li, H. P. Hratchian, A. F. Izmaylov, J. Bloino, G. Zheng, J. L. Sonnenberg, M. Hada, M. Ehara, K. Toyota, R. Fukuda, J. Hasegawa, M. Ishida, T. Nakajima, Y. Honda, O. Kitao, H. Nakai, T. Vreven, J. A. Montgomery, Jr., J. E. Peralta, F. Ogliaro, M. Bearpark, J. J. Heyd, E. Brothers, K. N. Kudin, V. N. Staroverov, R. Kobayashi, J. Normand, K. Raghavachari, A. Rendell, J. C. Burant, S. S. Iyengar, J. Tomasi, M. Cossi, N. Rega, J. M. Millam, M. Klene, J. E. Knox, J. B. Cross, V. Bakken, C. Adamo, J. Jaramillo, R. Gomperts, R. E. Stratmann, O. Yazyev, A. J. Austin, R. Cammi, C. Pomelli, J. W. Ochterski, R. L. Martin, K. Morokuma, V. G. Zakrzewski, G. A. Voth, P. Salvador, J. J. Dannenberg, S. Dapprich, A. D. Daniels, Ö. Farkas, J. B. Foresman, J. V. Ortiz, J. Cioslowski, and D. J. Fox, Gaussian 09 (Gaussian, Inc., Wallingford CT, 2009)

(8) Perdew, J. P.; Burke, K.; Ernzerhof, M. Generalized Gradient Approximation Made Simple. *Phys. Rev. Lett.* **1996**, *77* (18), 3865–3869.

(9) Grimme, S.; Antony, J.; Ehrlich, S.; Krieg, H. A Consistent and Accurate Ab Initio Parametrization of Density Functional Dispersion Correction (DFT-D) for the 94 Elements H-Pu. *J. Chem.Phys.* **2010**, *132* (15). https://doi.org/10.1063/1.3382344.

(10) Hariharan, P. C.; Pople, J. A. The Influence of Polarization Functions on Molecular Orbital Hydrogenation Energies. *Theor. Chim. Acta* **1973**, *28* (3), 213–222. https://doi.org/10.1007/BF00533485.

(11) Francl, M. M.; Pietro, W. J.; Hehre, W. J.; Binkley, J. S.; Gordon, M. S.; DeFrees, D. J.; Pople, J. A. Self-Consistent Molecular Orbital Methods. XXIII. A Polarization-Type Basis Set for Second-Row Elements. *J. Chem. Phys.* **1982**, *77* (7), 3654–3665. https://doi.org/10.1063/1.444267.

(12) Hehre, W. J.; Ditchfield, R.; Pople, J. A. Self—Consistent Molecular Orbital Methods. XII. Further Extensions of Gaussian—Type Basis Sets for Use in Molecular Orbital Studies of Organic Molecules. *J. Chem. Phys.* **1972**, *56* (5), 2257–2261. https://doi.org/10.1063/1.1677527.

(13) Andrae, D.; Häußermann, U.; Dolg, M.; Stoll, H.; Preuß, H. Energy-Adjusted *ab Initio* Pseudopotentials for the Second and Third Row Transition Elements. *Theor. Chim. Acta* **1990**, *77* (2), 123–141. https://doi.org/10.1007/BF01114537.

(14) Marenich, A. v.; Cramer, C. J.; Truhlar, D. G. Universal Solvation Model Based on Solute Electron Density and on a Continuum Model of the Solvent Defined by the Bulk Dielectric Constant and Atomic Surface Tensions. *J. Phys. Chem. B* **2009**, *113* (18), 6378–6396. https://doi.org/10.1021/jp810292n.

(15) Ribeiro, R. F.; Marenich, A. v.; Cramer, C. J.; Truhlar, D. G. Use of Solution-Phase Vibrational Frequencies in Continuum Models for the Free Energy of Solvation. *J. Phys. Chemistry B* **2011**, *115* (49), 14556–14562. https://doi.org/10.1021/jp205508z.

(16) Funes-Ardoiz, I.; Paton, R. S. GoodVibes: GoodVibes v2.0.2. p. https://doi.org/10.5281/zenodo.595246.

(17) Lu, T.; Chen, F. Multiwfn: A Multifunctional Wavefunction Analyzer. *J. Comput. Chem.* **2012**, *33* (5), 580–592. https://doi.org/10.1002/jcc.22885.

(18) Lebon, J.; Mortis, A.; Maichle-Mössmer, C.; Manßen, M.; Sirsch, P.; Anwander, R. Schlenk's Legacy—Methyllithium Put under Close Scrutiny. *Angew. Chem. Int. Ed.* ***2023***, 62, e202214599

(19) Sharma, A. K.; Sameera, W. M.; Jin, M.; Adak, L.; Okuzono, C.; Iwamoto, T.; Kato, M.; Nakamura, M.; Morokuma, K. DFT and AFIR Study on the Mechanism and the Origin of Enantioselectivity in Iron-Catalyzed Cross-Coupling Reactions. *J. Am. Chem. Soc.* **2017**, 139 (45), 16117–16125.

(20) Gutsev, L. G.; Gutsev, G. L.; Moore Tibbetts, K.; Jena, P. Homocoupling and Heterocoupling of Grignard Perfluorobenzene Reagents via Aryne Intermediates: A DFT Study. *J. Phys. Chem*. A **2019**, 123 (45), 9693–9700.

(21) Ikuma, N.; Nakagawa, K.; Kokubo, K.; Oshima, T. Regioselective addition of Grignard reagents to tosylazafulleroid and derivatization to 1,2-disubstituted [60]fullerene. *Org. Biomol. Chem*. **2016**, 14 (29), 7103–7108.

(22) Pedersen, M. J.; Born, S.; Neuenschwander, U.; Skovby, T.; Mealy, M. J.; Kiil, S.; Dam-Johansen, K.; Jensen, K. F. Optimization of Grignard Addition to Esters: Kinetic and Mechanistic Study of Model Phthalide Using Flow Chemistry. *Ind. Eng. Chem. Res.* **2018**, 57 (14), 4859–4866.

(23) Ren, Q.; Guan, S.; Jiang, F.; Fang, J. Density Functional Theory Study of the Mechanisms of Iron-Catalyzed Cross-Coupling Reactions of Alkyl Grignard Reagents. *J. Phys. Chem. A* **2013**, 117 (4), 756–764.

(24) Curado, N.; Carrasco, M.; Álvarez, E.; Maya, C.; Peloso, R.; Rodríguez, A.; López-Serrano, J.; Carmona, E. Lithium Di- and Trimethyl Dimolybdenum(II) Complexes with Mo-Mo Quadruple Bonds and Bridging Methyl Groups. *J. Am. Chem. Soc.* **2015**, *137* (38), 12378–12387. https://doi.org/10.1021/jacs.5b07899.

(25) Varadwaj, P. R.; Varadwaj, A.; Marques, H. M. DFT-B3LYP, NPA-, and QTAIM-Based Study of the Physical Properties of [M(II)(H_2O)$_2$(15-Crown-5)] (M = Mn, Fe, Co, Ni, Cu, Zn) Complexes. *J. Phys. Chem. A* **2011**, *115* (22), 5592–5601. https://doi.org/10.1021/jp2001157.

(26) Varadwaj, P. R.; Cukrowski, I.; Marques, H. M. DFT-UX3LYP Studies on the Coordination Chemistry of Ni2+. Part 1: Six Coordinate $[Ni(NH_3)_n(H_2O)_{6-n}]^{2+}$ Complexes. *J. Phys. Chem. A* **2008**, 112 (42), 10657–10666. https://doi.org/10.1021/jp803961s.
